# [2]Catenane Synthesis via Covalent Templating

**DOI:** 10.1002/chem.202004925

**Published:** 2021-01-14

**Authors:** Simone Pilon, Steen Ingemann Jørgensen, Jan H. van Maarseveen

**Affiliations:** ^1^ Van ‘t Hoff Institute for Molecular Sciences University of Amsterdam Science Park 904 1098XH Amsterdam The Netherlands

**Keywords:** catenanes, covalent template, mechanically interlocked molecules, planar chirality, template synthesis

## Abstract

After earlier unsuccessful attempts, this work reports the application of covalent templating for the synthesis of mechanically interlocked molecules (MiMs) bearing no supramolecular recognition sites. Two linear strands were covalently connected in a perpendicular fashion by a central ketal linkage. After subsequent attachment of the first strand to a template via temporary benzylic linkages, the second was linked to the template in a backfolding macrocyclization. The resulting pseudo[1]rotaxane structure was successfully converted to a [2]catenane via a second macrocyclization and cleavage of the ketal and temporary linkages.

## Graphical abstract

**A covalent template strategy** for the synthesis of mechanically interlocked molecules has been successfully employed to give a [2]catenane. This approach results in a [2]catenane devoid of supramolecular recognition sites. An inverted spiro intermediate is formed due to the action of a covalent template, which enforces an otherwise unfavorable macrocyclization. The trapped thread could be clipped into a second macrocycle giving a precatenane and, upon cleavage of the covalent templating linkages, the [2]catenane architecture.

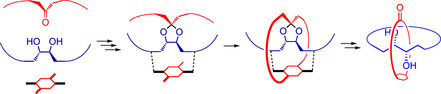

Over the last forty years, the field of mechanically interlocked molecule (MiM) synthesis has been dominated fully by non‐covalent templated approaches.[^1^, ^2^, ^3^] Taking advantage of specific supramolecular interactions, such as metal templating,[^4^, ^5^, ^6^] π–π stacking^[7]^ or hydrogen bonding,^[8]^ two or more molecular building blocks are preorganized into the desired topology, which is then fixed via covalent modifications. This results, in comparison to covalent approaches, in shorter and relatively higher yielding syntheses of MiMs, which explains the widespread application of these strategies in other fields, such as that of molecular machines.^[9]^ In addition, new non‐covalent approaches are being developed, such as radical and halogen bonding templation.[^10^, ^11^] However, the structural motifs required for supramolecular recognition tend to shape a large portion of the resulting MiMs, as well as their properties.

The development of covalent templated approaches, where reversible covalent bonds are employed to enforce the desired topology, can help widen the structural diversity of MiMs and open up new avenues.

Although less common, several covalent templates have recently seen application in MiMs synthesis. Drawing inspiration from passive metal templating, Godt et al. developed a carbonate template for the synthesis of [2]catenanes and polymeric catenanes.[^12^, ^13^, ^14^] Höger et al. successfully modified its terephthalic ester macrocyclization template to obtain MiMs, and our group later investigated this method as well.[^15^, ^16^, ^17^, ^18^] Other functional groups used for templating include esters[^19^, ^20^, ^21^, ^22^] and imines.[^23^, ^24^]

For this work however, we were specially inspired by Schill and colleagues, who, by the synthesis of a catenane, prepared a MiM for the first time and pioneered the field of covalently templated MiM synthesis.[^25^, ^26^, ^27^] In one study (Scheme 1, route **A**), they took advantage of a directing ketal group to join a macrocycle (in red) to a properly functionalized linear thread (in blue) in a perpendicular fashion.^[28]^ The resulting intermediate can adopt two conformations, a prerotaxane‐like one **1**, with the thread positioned within the ring, and a trivial one **1’**, which are in an equilibrium lying far to the left. Upon alkylation with bulky stopper groups, this equilibrium was frozen, and the two distinct compounds could be separated.

**Scheme 1:**
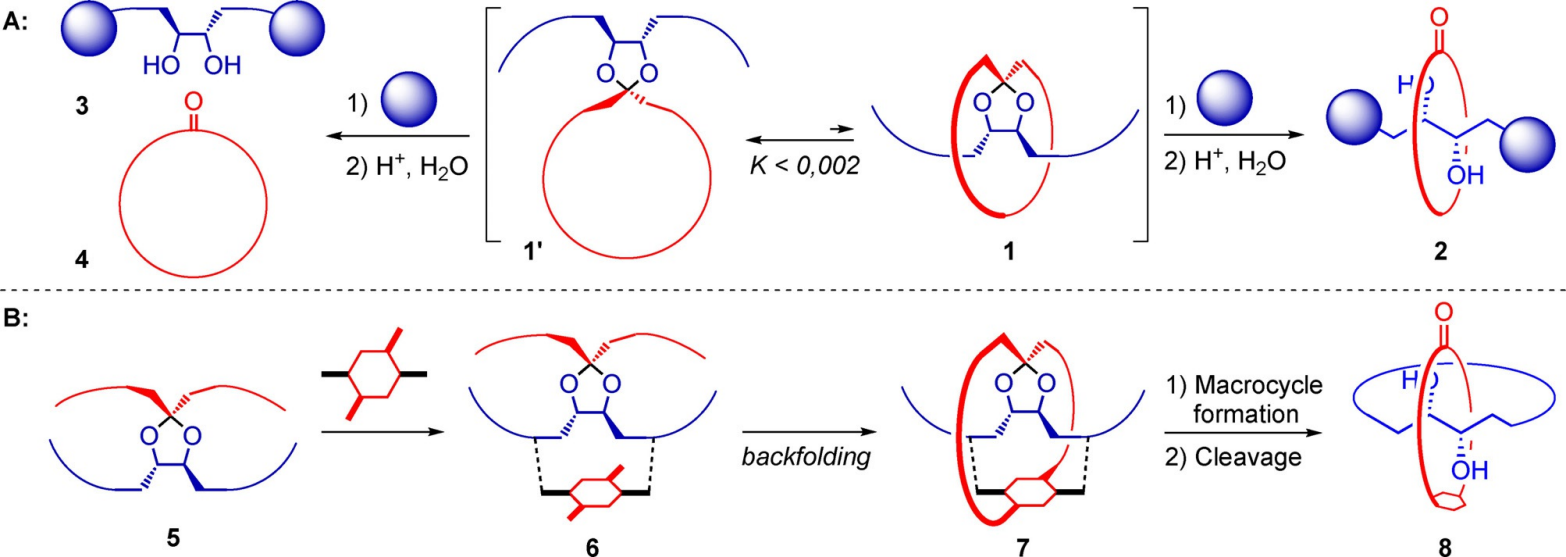
Comparison of Schill's statistical [2]rotaxane synthesis (**A**) with our covalent template backfolding strategy (**B**).

Acidic hydrolysis of the ketal moiety then gave a mixture of the separate ring and axle for the major intermediate and a [2]rotaxane for the very minor one (0.08–0.12 % yield). Despite the poor yields, it was not only proven that the prerotaxane conformation is possible, but also that it can in principle lead to an interlocked species.

We recently devised a somewhat similar approach to MiMs termed “templated backfolding” (Scheme 1, route **B**). Starting from two linear strands joined together at the center by a ketal, a suitable template is connected via temporary linkages (dashed lines) to give **6**. Next the first macrocycle is formed by linking the template to its opposing strand (in red) in a “backfolding” fashion. Thanks to the covalent temporary linkages, the unfavored pseudorotaxane conformation of **1** is the only one available to **7**.

From **7**, a second macrocycle (in blue) is formed, and final cleavage of the temporary linkages and the ketal selectively results in a [2]catenane. The efficacy of the backfolding approach was proven by the synthesis of both a quasi[1]rotaxane and a quasi[1]catenane, featuring irreversible bonds between the axle and ring fragments.^[29]^ The next step was to make the bond to the central quaternary spiro‐carbon reversible, by introducing a ketal group. With this purpose, target [2]catenane **10 a** (Scheme 2) was addressed.

**Scheme 2:**
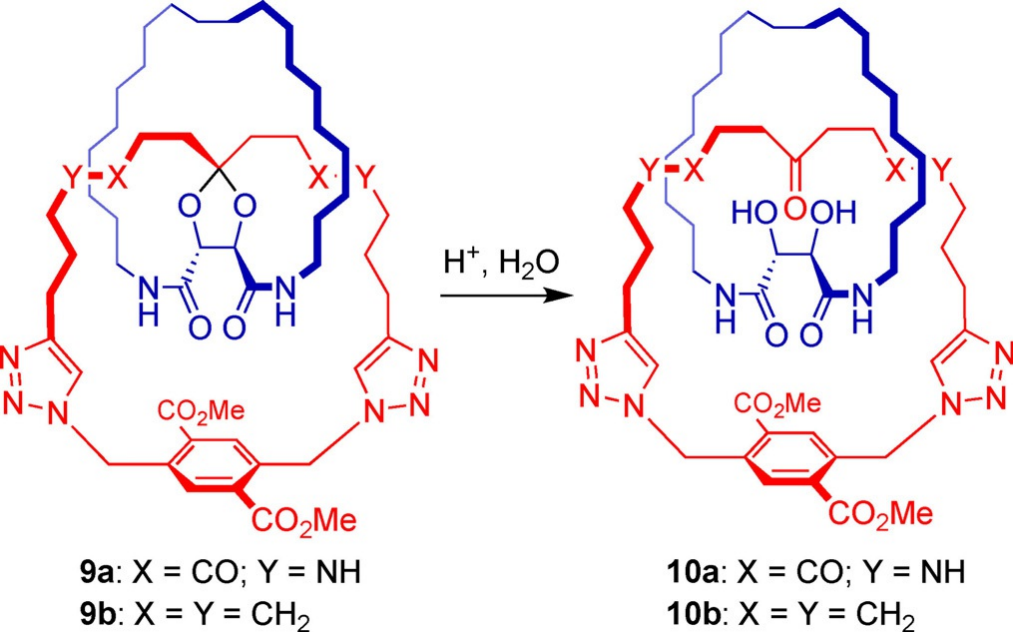
Planned ketal hydrolysis from precatenanes **9 a** and **9 b**.

By following the backfolding templated strategy as outlined in Scheme 1 B and using the powerful Cu^I^‐catalyzed azide‐alkyne cycloaddition (CuAAC) and ring‐closing metathesis (RCM) as the key macrocyclization steps, precatenane **9 a** was successfully obtained. However, to our surprise but even more disappointment, all attempts at hydrolyzing the acid labile ketal group failed.^[30]^ Initially this was attributed to steric shielding within the very congested precatenane architecture. In order to gather experimental evidence of this, model compound **11** was synthesized, which closely matches the electronic environment of precatenane **9 a** (Scheme 3). Treatment of ketal **11** with concentrated aqueous HCl in MeOH at room temperature showed even after 28 h only trace amounts of the hydrolysis products. Hydrolysis of ketal **11** could only be accomplished after stirring in concentrated HCl and MeOH at 50 °C for several hours. This indicates that besides the catenane effect that clearly plays a role in the remarkable stability of ketal **9 a**, other factors play a role. In contrast, ketal **12**, an early intermediate in synthesis of **9 a**, could be hydrolyzed at room temperature in only 4 h under otherwise identical conditions. The positive influence of amide groups on the stability of nearby ketals has been reported before^[31]^ and this observation inspired us to pursue the synthesis of **10 b**, an analog of catenane **10 a** in which the amides have been replaced by ethylidene groups.

**Scheme 3:**
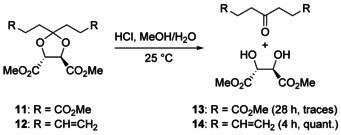
Model study to assess reactivity of the central ketal linkage.

For this, ketone **17** was prepared first from known alcohol **15** ^[32]^ in three steps (Scheme 4). The alkene groups were converted to alkynes via a bromination‐elimination protocol, which proceeds in good selectivity under anhydrous conditions.

**Scheme 4:**
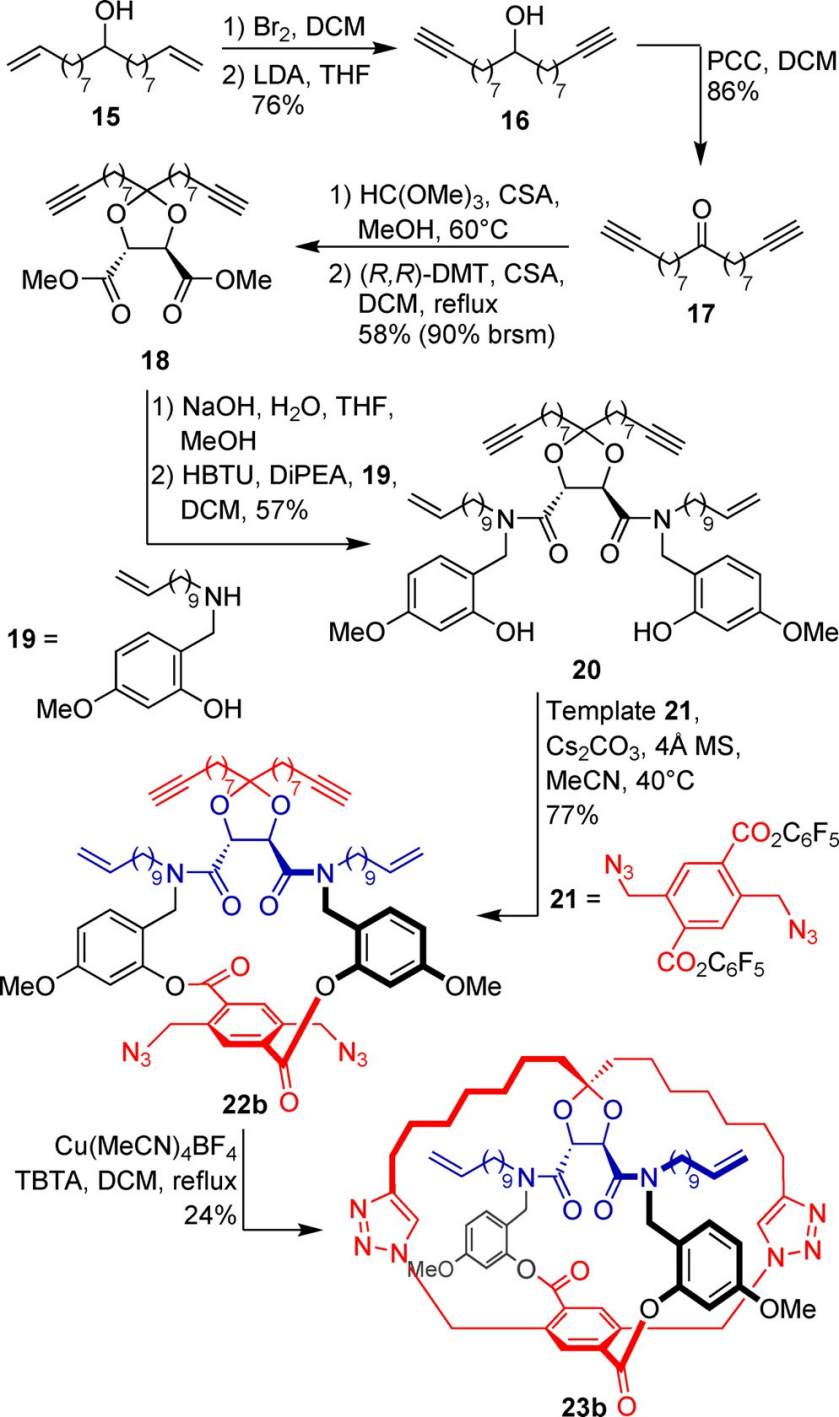
Synthesis of precatenane **23 b**, the common [2]catenane and [2]rotaxane precursor.

This was followed by oxidation of the alcohol group by PCC. As already found in our previous studies, direct coupling of ketones such as **17** and (+)‐dimethyl tartrate proceeds sluggishly. This could be overcome by transformation of the ketone to the dimethoxy ketal, followed by transketalization with (+)‐dimethyl tartrate to give functionalized ketal **18** in 58 % yield. Although more viable, this procedure suffers from incomplete conversion, likely due to the difficulties in completely removing water from the reactants, but nonetheless allows for recovery of ketone **17** in high yield (90 % brsm). Assembly of the second macrocycle follows, by saponification of the methyl esters and coupling of the resulting diacid with amine **19**, which also includes the temporary linkages. At this stage, transesterification of template **21** with the two phenolic groups in **20** affords the backfolding macrocyclization precursor **22 b**. To initiate this key step using the CuAAC reaction, **22 b** was stirred at high dilution with Cu(MeCN)_4_BF_4_ as catalyst and TBTA as ligand. Refluxing in CH_2_Cl_2_ for 3 days gave cage compound **23 b** in 24 % yield. Compared to **23 a**,^[30]^ **23 b** is formed in lower yield and showed a much simpler ^1^H‐NMR spectrum, with only minor splitting of the signals corresponding to chemically equivalent protons. These surprising differences suggest the presence of substantial interactions between the macrocycle amides and the tartrate core in **23 a**, resulting in rigidification of the macrocycle (in red) and slowing down conformational exchange.

With the ketal and two macrocycles in place, the two terminal alkenes are positioned such as to allow the second macrocyclization to give the precatenane skeleton. This is done via RCM, performed in CH_2_Cl_2_ at 40 °C using Grubbs’ 2^nd^ generation catalyst (Scheme 5). The resulting product, obtained in 57 % yield as an inseparable mixture of E and Z isomers, is then converted to a single compound **24 b‐H_2_** after saturation of the double bonds by catalytic hydrogenation. To liberate the [2]catenane, first the temporary linkages were broken via solvolytic transesterification of the lactone groups, followed by protolytic cleavage of the benzylic tertiary amides using TFA in the presence of Et_3_SiH as cation scavenger. Finally, the ketal core was hydrolyzed under strongly acidic conditions, liberating [2]catenane **10 b** in 60 % yield.

**Scheme 5:**
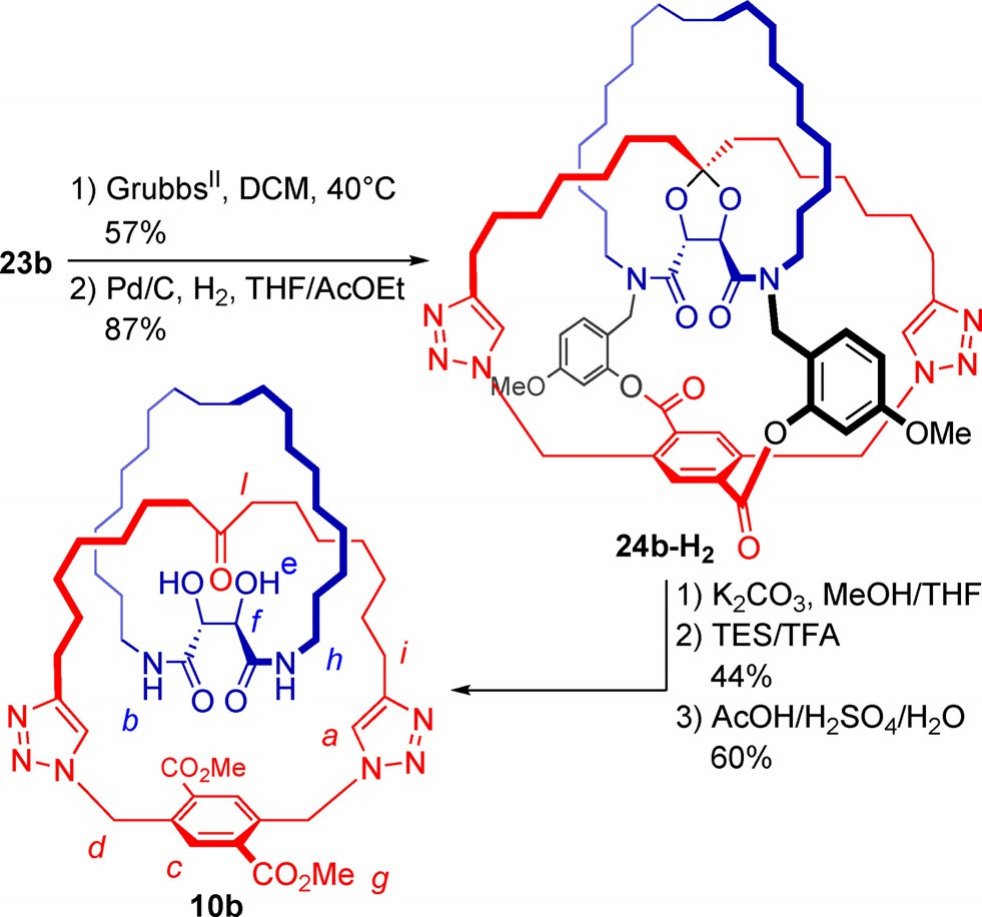
Synthesis of [2]catenane **10 b**.

To further assess the influence of the catenane effect on the stability of the ketal group in precatenanes **9 a** and **9 b**, we set out to synthesize their respective regular spiro topoisomers and subjected them to ketal hydrolysis conditions (Scheme 6).

**Scheme 6:**
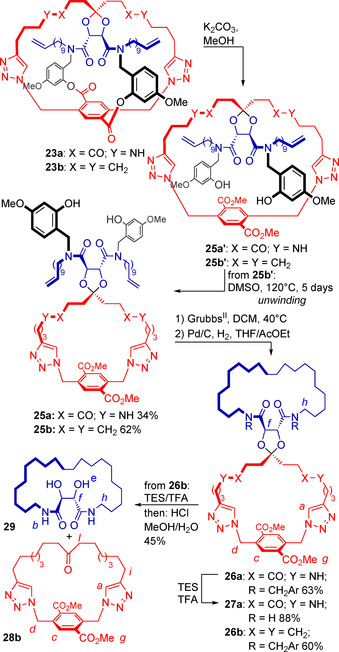
Formation of trivial rings **29** and **28 b**.

This was done by simply altering the order of the final steps that were used for the syntheses of precatenanes **9 a** and **9 b**. Thus starting from prerotaxane **23 a**, instead of first carrying out the RCM macrocyclization reaction, the sequence of reactions started by solvolysis of the temporary ester linkages in **23 a** to give macrocycle **25 a’** as an intermediate, followed by a concomitant spontaneous unwinding process, yielding **25 a**. Subsequent subjection of **25 a** to RCM, catalytic hydrogenation conditions and final protolytic removal of the benzylic appendages gave a compound with different spectral properties from precatenane **9 a**, supporting the proposed regular spiro topology of **27 a** thus obtained.

As shown in Figure 1, a comparison of the ^1^H‐NMR spectra of **27 a** and **9 a** shows marked differences for several key peaks. These were assigned for **27 a** based on integral and multiplicity, and their identity was confirmed by COSY and HSQC NMR (see supporting information). Most striking, is the splitting of the diastereotopic benzylic protons (*d*), which is significantly increased in precatenane **9 a**. Similarly, the aliphatic signals between 1.5 and 3 ppm as well as the amide N*H* signals display much more complex patterns in **9 a**. This is likely the result of slower conformational movements in the sterically more congested precatenane macrocycles.^[33]^ In addition, the aromatic template signal (*c*) is shifted downfield by 0.34 ppm, indicating additional shielding in **27 a** due to ring‐current effects arising from the triazole moieties.^[34]^ This interaction is reduced in **9 a** as the intra‐annular fragment limits the ability of the ketone macrocycle (in red) to fold. These findings are consistent with observations made on similar inverted spiro architectures previously synthesized via our backfolding approach.[^29^, ^35^]

**Figure 1:**
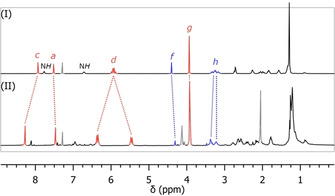
Comparison of ^1^H‐NMR spectra of trivial spiro bicycle **27 a** (I) and precatenane **9 a** ^[30]^ (II) in CDCl_3_. For proton assignments, see Scheme 6.

Remarkably, solvolysis of the ester linkages in **23 b** gave intermediate **25 b’** as a stable compound at room temperature, which required extensive heating to unwind to the thermodynamically favored conformer **25 b**. Molecular mechanics modeling of structures **25 b’** and **25 a’** suggest that the benzylic groups (in black) could hamper this process, as they barely fit in the macrocycle cavity according to space filling representations (see supporting information). However, so far we have no sound explanation for the exceptional differences in the kinetics of this step between **25 a’** and **25 b’**. From unwound macrocycle **25 b**, the trivial spiro bismacrocycle **26 b** was obtained after RCM and catalytic hydrogenation.

Similarly to what was observed for precatenanes **9 a** and **9 b**, the final hydrolysis of the linking ketals in the regular spiro compounds **26 b** and **27 a** gave a completely different outcome (Scheme 6). Starting from **26 a**, the benzylic groups could be selectively cleaved in TFA in the presence of Et_3_SiH, giving **27 a**, which failed to hydrolyze to the desired separate macrocycles even under forcing conditions (not shown). In stark contrast, **26 b** partially split into the separate macrocycles **29** and **28 b** already by TFA addition. This process was brought to completion by treatment with HCl in MeOH/H_2_O at room temperature. The relatively mild conditions required underscore that the high stability of the ketal in precatenanes **9 a**,**b** emerges to a great extent from the catenane effect. Due to the presence of the additional ketal stabilizing amide groups in **9 a**, in this case ketal cleavage is completely blocked.

Analysis by ^1^H‐NMR revealed marked differences between catenane **10 b** and a 1:1 mixture of its separate and non‐interlocked components **28 b** and **29**, as shown in Figure 2. The benzylic protons (*d*) appear equivalent in **28 b**, and become diastereotopic in **10 b**. In addition, the overall chemical shift of the aliphatic proton signals is significantly reduced. This points to increased shielding and is common for methylene chains entrapped within macrocycles bearing aromatic rings.[^33^, ^36^, ^37^] However, the most striking difference is the downfield shift of 0.56 and 0.11 ppm, respectively, of the template aromatic (*c*) and methyl ester (*g*) protons. As was observed for precatenane **9 a** and trivial bicycle **27 a**, this is probably due to the rigid conformation of the interlocked rings in [2]catenane **10 b** preventing the macrocycles from collapsing. Furthermore, the NMR spectrum of catenane **10 b** shows that the signals of both the terephthalate protons (*c*) and triazole protons (*a*) appear as double singlets (Figure 3). This points to the presence of two diastereomeric forms of catenane **10 b**, likely resulting from a combination of central chirality, emerging from the tartrate moiety, and planar chirality of the red macrocycle. The AB system displayed by the benzylic protons (*d*) suggests in fact that rotation of the *p*‐cyclophane‐type terephthalate moiety is hindered, resulting in two different conformations of the macrocycle, which are mirror images of each other (Figure 3, III).

**Figure 2:**
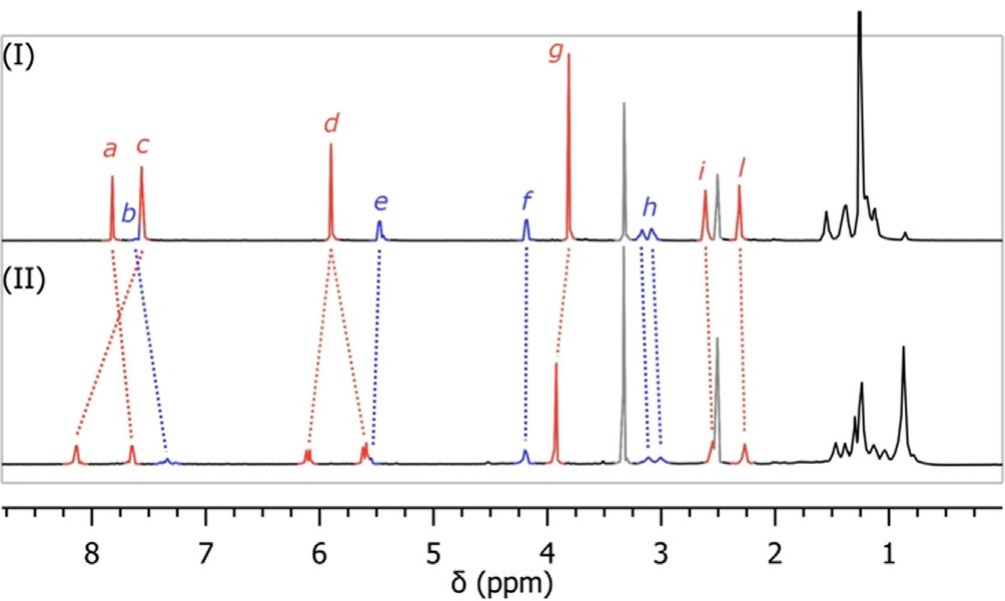
Comparison of ^1^H‐NMR spectra of catenane **10 b** (II) with an equimolar mixture of trivial rings **28 b** and **29** (I) in [D_6_]DMSO. For proton assignments, see Schemes 5 and 6.

**Figure 3:**
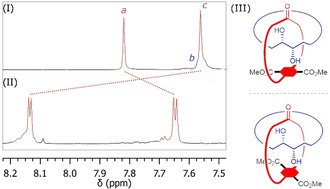
Detail of the ^1^H‐NMR spectra of catenane **10 b** (II) and an equimolar mixture of trivial rings **28 b** and **29** (I) in [D_6_]DMSO showing splitting of the aromatic peaks. The proposed structure of the two diastereoisomers of **10 b** is shown (III).

Conversely, the non‐interlocked macrocycle **28 b**, which lacks the steric constraints of the mechanical bond, displays a sharp singlet signal for the benzylic (*d*) protons, indicating that the terephthalic moiety can rotate freely. So far, all attempts to separate the eventual diastereomers of **10 b** by several symmetric and asymmetric HPLC methods failed.

In conclusion, despite the strong stabilizing catenane effect experienced by the pivotal ketal link, small structural changes allowed its hydrolysis liberating the mechanically interlocked [2]catenane. As a consequence, the covalent template backfolding approach was, for the first time, successfully employed in the synthesis of a mechanically interlocked molecule. Currently, we are focusing on reducing the footprint of the template and further improving the versatility of the temporary linkages to expand the structural diversity within the class of mechanically interlocked molecules.

## Conflict of interest

The authors declare no conflict of interest.

## Supporting information

As a service to our authors and readers, this journal provides supporting information supplied by the authors. Such materials are peer reviewed and may be re‐organized for online delivery, but are not copy‐edited or typeset. Technical support issues arising from supporting information (other than missing files) should be addressed to the authors.

Supplementary
