# Supplementary material for: [2]Catenane Synthesis via Covalent Templating
Source: Chemistry. 2021 Jan 14;27(7):2310–4. doi: 10.1002/chem.202004925 (PMC7898614; doi:10.1002/chem.202004925)
Supplement: Supplementary file 1 — Supplementary [file CHEM-27-2310-s001.pdf]

Chemistry–A European Journal

Supporting Information

[2]Catenane Synthesis via Covalent Templating

Simone Pilon, Steen Ingemann Jørgensen, and Jan H. van Maarseveen*[a]

Table of Contents

Experimental Procedures 1
NMR Spectra 11
Mass Spectra 42
Molecular mechanics 45
References 50

Experimental Procedures

General methods and materials

Reactions were carried out under air and without additional measures such as drying unless stated otherwise. Heating and stirring was performed using oil baths and standard thermostatized stirring plates and Teflon stirring beans. Thin layer chromatography (TLC) was performed on Merck TLC plates (0.25 mm) precoated with silica gel 60 F_{254}. Flash column chromatography was performed using Macherey-Nagel Silica 60 (particle size 0.04–0.063 mm) under compressed air flow or a Buchi C-850 automatic column machine with FlashPure silica cartridges. Where PE is noted as an eluent, 40-60°C petroleum ether was used. Starting materials and reagents were used as supplied by commercial vendors. Anhydrous CH_2Cl_2, MeCN and THF were obtained from pre-dried materials via an MBRAUN SPS-800 machine and stored under N_2 atmosphere, anhydrous MeOH was dried over 3Å molecular sieves (MS) for 20 hours and stored over fresh 3Å MS under a N_2 atmosphere. MS were dried at 250°C *in vacuo* for 8 hours before use and stored under N_2 atmosphere. Bruker DRX-300, 400 and 500 MHz instruments were used to record NMR spectra. Chemical shifts (δ) are reported in ppm relative to residual undeuterated solvent peaks. Data of the recorded ^{1}H NMR spectra are described as follows: chemical shift (multiplicity, coupling constant when applicable, number of H). The following abbreviations are used to report the multiplicities: s (singlet), d (doublet), t (triplet), q (quartet), quint (quintet), dd (doublet of doublet), m (multiplet). High-resolution mass spectra (HRMS) were recorded on an AccuTOF GC v 4g, JMST100GCV mass spectrometer (JEOL, Japan) and HR-ToF Bruker Daltonik GmbH (Bremen, Germany) Impact II, an ESI-ToF MS capable of resolution of at least 40,000 FWHM. The FD/FI probe was equipped with an FD Emitter, Carbotec, FD = 10 µm. Current rate = 51.2 mA/min over 1.2 min using field desorption (FD) as an ionization method. Melting points were recorded on a Buchi M-565 melting point apparatus. IR spectra were recorded on a Bruker Alpha FTIR apparatus.

Ketal (12):

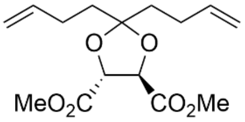

Nona-1,8-dien-5-one[1] (518 mg, 3.75 mmol, 1.5 equiv), pTsOH (95 mg, 0.50 mmol, 0.2 equiv) and trimethyl orthoformate (2.1 mL, 18.7 mmol, 7.5 equiv) were dissolved in 15 mL of MeOH and stirred overnight at room temperature. The reaction mixture was quenched with $NaHCO_3$ (500 mg) and stirred for 10 minutes, then concentrated *in vacuo*. The residue was partitioned between 10 mL of H_2O and 10 mL of EtOAc, the aqueous phase was extracted 2 × 10 mL EtOAc. The reunited organic phases were washed with brine, dried over $MgSO_4$ and concentrated *in vacuo*.
The residue was dissolved in 15 mL of anhydrous CH_2Cl_2 and pTsOH (95 mg, 0.50 mmol, 0.2 equiv) and (+)-dimethyl L-tartrate (445 mg, 2.5 mmol, 1 equiv) were added. The reaction mixture was heated at reflux overnight, then washed with 10 mL of saturated aqueous $NaHCO_3$. The aqueous phase was extracted 2 × 10 mL CH_2Cl_2 and the reunited organic phases were dried over $MgSO_4$ and dry loaded onto silica. Purification by column chromatography (PE/EtOAc 9:1 → 8:2) afforded ketal **12** (438 mg, 59%).^{1}H NMR data matched those reported in literature.[1]

Diacid (30):

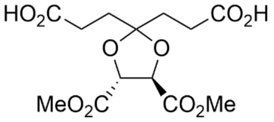

Ketal **12** (300 mg, 1 mmol, 1 equiv) was dissolved in 20 mL of anhydrous CH_2Cl_2 and the solution was cooled to -78 °C and O_3 was bubbled until the solution turned bright blue (ca. 20 minutes), then O_2 was bubbled until the solution turned back to colorless and PPh_3 (577 mg, 2.2 mmol, 2.2 equiv) was added and the mixture was allowed to warm to room temperature and stirred overnight. The mixture was then concentrated *in vacuo* and dissolved in 20 mL of tBuOH. A 0.5 M solution of NaH_2PO_4 in water (7 mL) and 2-methyl-2-butene (2.1 mL, 20 mmol, 20 equiv) were added to the mixture, which was then cooled to 0 °C before adding $NaClO_2$ (723 mg, 8 mmol, 8 equiv) in 1 mL H_2O dropwise. The resulting reaction mixture was stirred for 4 hours at 0 °C, then 20 mL of 5% K_2CO_3 in water was added and washed 2 × 20 mL EtOAc. The organic phases were extracted 2 × 20 mL 5% K_2CO_3 and the combined aqueous phases were acidified to pH ~3 with 2 M HCl and extracted 3 × 30 mL EtOAc. The reunited organic phases were dried over $MgSO_4$ and concentrated *in vacuo* to give diacid **30** (191 mg, 60%).^{1}H NMR data matched those reported in literature.[1]

Tetra methyl ester (11):

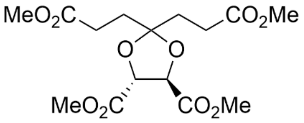

Diacid **30** (171 mg, 0.534 mmol, 1 equiv) was dissolved in 10 mL of anhydrous THF and DBU (319 µL, 2.10 mmol, 4 equiv) and MeI (166 µL, 2.70 mmol, 5 equiv) were added. The reaction mixture was stirred at room temperature for 5 hours, then poured into 50 mL of saturated aqueous NH_4Cl and extracted 3 × 40 mL of EtOAc. The reunited organic phases were washed with brine, dried over $MgSO_4$ and concentrated *in vacuo*. The crude product was dry loaded onto silica and purified by column chromatography (heptane/EtOAc 1:1) to give tetra methyl ester **11** (28.8 mg, 15%) as a colorless oil. R_f=0.53 (PE/EtOAc 1:1); ^{1}H NMR (400 MHz, $CDCl_3$): δ=4.73 (s, 2H), 3.83 (s, 6H), 3.67 (s, 6H), 2.47 (t, J= 8 Hz, 4H), 2.07 (m, 4H); ^{13}C NMR (100 MHz,

$CDCl_3$): δ=173.4, 169.3, 115.1, 77.2, 52.9, 51.7, 32.0, 28.3.; IR (neat): $\tilde{\nu}$=2955, 1732, 1438, 1199, 1174, 1129, 1072, 988, 890, 750 cm^{-1}; Low resolution MS (FD$^+$) *m/z* calcd for $C_{15}H_{23}O_{10}^+$ [M+H]$^+$ 363.12, found 363.12.

Hydrolysis of model ketals 11 and 12:

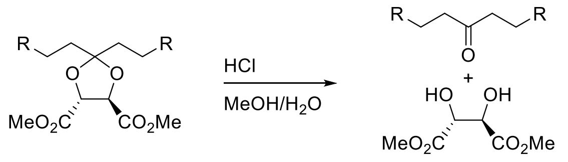

11: n = 0, R = CO_2Me **13**: n=0, R = CO_2Me
12: n = 0, R = CH=CH_2 **14**: n=0, R = CH=CH_2

The starting material (6 mg) was dissolved in 0.5 mL of MeOH and 0.5 mL of 32% HCl was added. The reaction mixture was stirred at the required temperature and the progress was monitored via TLC (PE/EtOAc 8:2 for **12** and PE/EtOAc 1:1 for **11**). Once complete, 2 mL of H_2O were added to the reaction mixture and extracted 3 × 20 mL of CH_2Cl_2, the reunited organic phases were washed with 20 mL of saturated aqueous $NaHCO_3$, dried over $MgSO_4$ and concentrated *in vacuo*. In the case of **11**, concentration was performed at room temperature and at a minimum of 100 mBar of pressure to prevent loss of the volatile ketone product. Crude ^{1}H-NMR was measured to confirm the conversion of the starting material to the expected products.

Table 1. Conditions for the hydrolysis of model substrates **11** and **12**.

Starting material	Temperature [°C]	Time [hours]	Conversion
**12**	25	4	Complete
**11**	25	28	Traces
**11**	50	4	Complete

Nonadeca-1,18-dien-10-ol (15):

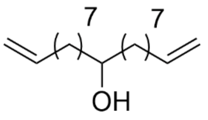

Mg turnings (695 mg, 28.6 mmol, 2.36 equiv) were placed in an oven-dried 3-necked flask with a few crystals of Iodine under a N_2 atmosphere. To this, a solution of 9-bromo-1-nonene (4.96 g, 24.2 mmol, 2 equiv) in 30 mL of anhydrous THF was added at a rate sufficient to maintain reflux. Once the addition was complete, the mixture was heated to reflux for 4.5 hours, then gradually cooled to 0°C prior to adding ethyl formate (896 mg, 12.1 mmol, 1 equiv) in 10 mL of anhydrous THF. The reaction mixture was then heated to reflux for 16 hours, cooled to room temperature and poured into 50 mL of saturated aqueous NH_4Cl and extracted with 3 × 40 mL of EtOAc, the reunited organic phases were washed with brine, dried over $MgSO_4$ and concentrated. The crude mixture was dry-loaded onto silica and purified by column chromatography (PE/EtOAc 40:1 → 40:2 → 40:3) to give nonadeca-1,18-dien-10-ol (**15**) (2.26 g, 67%) as a white solid. ^{1}H NMR data matched those reported in literature. [2]

R$_f$=0.37 (PE/EtOAc 9:1); m.p. 43.0–44.0°C; ^{1}H NMR (400 MHz, $CDCl_3$): δ=5.89–5.75 (m, 2H), 5.03 (dd, *J*=17, 2 Hz, 2H), 4.94 (dd, *J*=10, 2 Hz, 2H), 3.60 (m, 1H), 2.06 (q, *J*=7 Hz, 4H), 1.52–1.22 (m, 24H); ^{13}C NMR (100 MHz, $CDCl_3$): δ=139.2, 114.1, 72.0, 37.5, 33.8, 29.7, 29.5, 29.1, 28.9, 25.6; IR (neat): $\tilde{\nu}$=3360, 3077, 2977, 2925, 2854, 1640, 1464, 1415, 1370, 993, 906, 731, 648, 555 cm^{-1};

Nonadeca-1,18-diyn-10-ol (16):

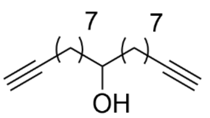

Nonadeca-1,18-dien-10-ol (**15**) (1.96 g, 7.00 mmol, 1 equiv) was dissolved in 28 mL of anhydrous CH_2Cl_2 and cooled to 0°C under a N_2 atmosphere. To this, a solution of Br_2 (790 µL, 15.4 mmol, 2.2 equiv) in 12 mL of anhydrous CH_2Cl_2 was added dropwise over 10 minutes, and the resulting solution was stirred at room temperature for 2 hours. The solvent and excess Br_2 were then distilled off under reduced pressure, taking care to maintain the N_2 atmosphere. The resulting oil was dissolved in 20 mL of anhydrous THF and the resulting solution was added dropwise to LDA in 40 mL of THF at -78°C, which was prepared from iPr_2NH (8.9 mL, 63 mmol, 9 equiv) and 2.5 M nBuLi in hexanes (22.4 mL, 56 mmol, 8 equiv). After 1 hour the mixture was allowed to warm to room temperature for 3.5 hours after which time it was poured into 60 mL of aqueous saturated NH_4Cl and extracted with 60 mL Et_2O and 2 × 30 mL Et_2O. The reunited organic phases were then washed with 60 mL aqueous saturated NH_4Cl, brine, dried over $MgSO_4$ and concentrated *in vacuo*. The crude product was dry-loaded onto silica and purified by column chromatography (PE/EtOAc 19:1 → 9:1) to give nonadeca-1,18-diyn-10-ol (**16**) (1.48 g, 76%) as a white solid.

R$_f$=0.38 (PE/EtOAc 8:2); m.p. 51.6–54.4°C; ^{1}H NMR (400 MHz, $CDCl_3$): δ=3.56 (m, 1H), 2.17 (td, *J*=7, 3 Hz, 4H), 1.93 (t, *J*=3 Hz, 2H), 1.52 (p, *J*=7 Hz, H4), 1.47–1.23 (m, 20H); ^{13}C NMR (100 MHz, $CDCl_3$): δ=84.7, 71.9, 68.1, 37.4, 29.5, 29.1, 28.7, 28.4, 25.6, 18.4; IR (neat): $\tilde{\nu}$=3284, 2922, 2849, 1465, 1427, 1283, 1145, 1121, 1067, 1050, 1007, 926, 866, 723, 645, 535, 522 cm^{-1}; HRMS (FD$^+$): *m/z* calcd for $C_{19}H_{33}O_1$ [M+H]$^+$ 277.2526; found: 277.2546;

Nonadeca-1,18-diyn-10-one (17):

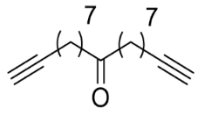

Nonadeca-1,18-diyn-10-ol (**16**) (1.40 g, 5.06 mmol, 1 equiv) was dissolved in 50 mL of CH_2Cl_2, followed by addition of silica (1.75 g) and PCC (1.75 g, 8.10 mmol, 1.6 equiv). The resulting mixture was stirred at room temperature for 22 hours, filtered over celite and dry-loaded onto silica to be purified by column chromatography (PE/EtOAc 40:1 → 20:1) which gave nonadeca-1,18-diyn-10-one (**17**) (1.20 g, 86%) as a white solid.
R_f=0.41 (PE/EtOAc 9:1); m.p. 36.7–37.4°C; ^{1}H NMR (400 MHz, $CDCl_3$): *δ*=2.39 (t, *J*=7 Hz, 4H), 2.18 (td, *J*=7, 3 Hz, 4H), 1.94 (t, *J*=3 Hz, 2H), 1.62–1.47 (m, 8H), 1.45–1.23 (m, 12H); ^{13}C NMR (100 MHz, $CDCl_3$): *δ*=211.4, 84.6, 68.1, 42.7, 29.1, 28.9, 28.5, 28.4, 23.8, 18.4; IR (neat): $\tilde{v}$=3293, 3285, 2930, 2917, 2850, 1702, 1696, 1468, 1443, 1419, 1380, 1355, 1332, 1251, 1113, 1059, 992, 975, 721, 680, 642, 533, 411 cm^{-1}; HRMS (FI$^+$): *m/z* calcd for $C_{19}H_{30}O_1^+$ ($M^{\cdot+}$) 274.2291; found: 274.2280;

Ketal (18):

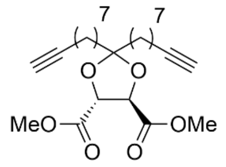

Nonadeca-1,18-diyn-10-one (**17**) (2.13 g, 7.77 mmol, 1 equiv), camphorsulfonic acid (361 mg, 1.55 mmol, 0.2 equiv) and trimethyl orthoformate (4.25 mL, 38.9 mmol, 5 equiv) were dissolved in 50 mL of MeOH and stirred under a N_2 atmosphere at 60°C for 19 hours. Then the reaction mixture was cooled to room temperature and $NaHCO_3$ (780 mg, 9.29 mmol, 1.2 equiv) was added. After 10 minutes of vigorous stirring, the mixture was concentrated *in vacuo*, diluted in 50 mL of Et_2O and washed with 50 mL of aqueous saturated $NaHCO_3$, brine, dried over $MgSO_4$ and concentrated *in vacuo*. The resulting pale yellow oil was dissolved in 40 mL anhydrous CH_2Cl_2, to which (+)-dimethyl L-tartrate (2.77 g, 15.5 mmol, 2 equiv) and 4Å MS (2.5 g) were added. After stirring for 2 hours, the suspension was filtered over celite with 30 mL of anhydrous CH_2Cl_2 and transferred to a flame dried reflux setup containing camphorsulfonic acid (361 mg, 1.55 mmol, 0.2 equiv) where the mixture was heated to reflux for 19 hours. The mixture was then allowed to cool to room temperature and was washed with 50 mL of aqueous saturated $NaHCO_3$, brine, dried over $MgSO_4$ and concentrated *in vacuo*. The crude product was dry-loaded onto silica and purified by column chromatography (PE/EtOAc 40:1 (recover SM) → 20:1 → 9:1) to give ketal **18** (1.97 g, 58%) as a faint yellow oil and nonadeca-1,18-diyn-10-one (**17**) (687 mg, 32%) as a faint yellow solid.
R_f=0.34 (PE/EtOAc 8:2); ^{1}H NMR (400 MHz, $CDCl_3$): *δ*=4.71 (s, 2H), 3.81 (s, 6H), 2.16 (td, *J*=7, 3 Hz, 4H), 1.93 (t, *J*=3 Hz, 2H), 1.66 (m, 4H), 1.50 (p, *J*=7 Hz, 4H), 1.44–1.22 (m, 16H); ^{13}C NMR (100 MHz, $CDCl_3$): *δ*=169.8, 117.2, 84.6, 77.0, 68.1, 52.7, 37.0, 29.5, 28.9, 28.6, 28.4, 23.5, 18.3; IR (neat): $\tilde{v}$=3291, 2931, 2856, 1746, 1437, 1372, 1280, 1206, 1123, 1105, 1017, 942, 877, 807, 750, 725, 627, 556, 527 cm^{-1}; HRMS (FI$^+$): *m/z* calcd for $C_{25}H_{39}O_6^+$ $[M+H]^+$ 435.2741; found: 435.2746;

Diamide (20):

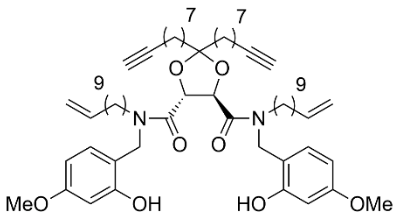

Diester **18** (1.97 g, 4.54 mmol, 1 equiv) was dissolved in 36 mL of THF and 12 mL of MeOH and 24 mL of H_2O were added. Finally, 1 M aqueous NaOH (45 mL, 45 mmol, 10 equiv) was added and the resulting suspension was stirred for 17 hours at room temperature and then quickly concentrated *in vacuo* to remove most of the THF and MeOH. The resulting mixture was cooled to 0°C and acidified to pH ~3 with 1 M aqueous HCl, then extracted 2 × 50 mL of EtOAc. The aqueous phase was further acidified to pH ~2 and extracted 2 × 50 mL of EtOAc. Then the reunited organic phases were dried over $MgSO_4$ and concentrated *in vacuo*. The resulting solid was dissolved in anhydrous CH_2Cl_2 and cooled to 0°C. Then, iPr_2EtN (6.0 mL, 36 mmol, 8 equiv) and PyBOP (6.14 g, 11.8 mmol, 2.6 equiv) were added, followed after 5 minutes by amine **19**[3] (3.05 g, 9.99 mmol, 2.2 equiv). The resulting mixture was stirred at room temperature for 3 hours and then at 40°C for 15 hours, after which it was washed with 2 × 60 mL of 1 M aqueous HCl, brine, dried over $MgSO_4$ and concentrated *in vacuo*. The crude product was dry-loaded onto silica and purified by column chromatography (PE/EtOAc 19:1 → 9:1 → 8:2) to give diamide **20** (2.54 g, 57%) as a yellow oil.
R_f=0.58 (PE/EtOAc 7:3); ^{1}H NMR (400 MHz, $CDCl_3$): *δ*=9.40 (s, 2H), 7.00 (d, *J*=8 Hz, 2H), 6.48 (d, *J*=3 Hz, 2H), 6.40 (dd, *J*=8, 3 Hz, 2H), 5.84 (m, 2H), 5.20 (s, 2H), 5.08–4.92 (m, 4H), 4.47–4.31 (m, 4H), 3.78 (s, 6H), 3.45 (dd, *J*=10, 6 Hz, 4H), 2.19 (td, *J*=7, 3 Hz, 4H), 2.08 (q, *J*=7 Hz, 4H), 1.95 (t, *J*=3 Hz, 2H), 1.74 (m, 4H), 1.62 (m, 4H), 1.53 (p, *J*=7 Hz), 1.45–1.23 (m, 44H); ^{13}C NMR (100 MHz, $CDCl_3$): *δ*=170.0, 161.5, 157.5, 139.1, 132.1, 116.3, 114.3, 114.2, 105.8, 102.6, 84.7, 75.5, 68.1, 55.2, 47.4, 46.2, 37.1, 33.8, 29.6, 29.5, 29.4, 29.3, 29.1, 29.0, 28.9, 28.7, 28.5, 28.3, 26.9, 23.8, 18.4; IR (neat): $\tilde{v}$=3309, 3076, 2925, 2853, 1615, 1506, 1463, 1440, 1367, 1324, 1288, 1260, 1201, 1159, 1115, 1099, 1036, 994, 965, 909, 839, 785, 723, 690, 631, 577, 490, 460, 438 cm^{-1}; HRMS (FD$^+$): *m/z* calcd for $C_{61}H_{92}N_2O_8^+$ ($M^{\cdot+}$) 980.6848; found: 980.6842;

Diester (22b):

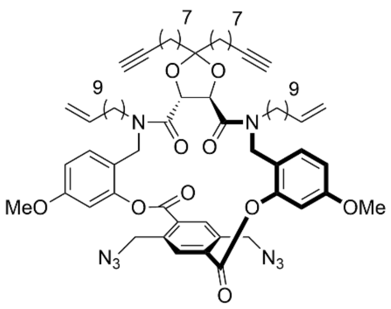

Diphenol **20** (981 mg, 1.00 mmol, 1 equiv) was added to a suspension of Cs_2CO_3 (3.26 g, 10.0 mmol, 10 equiv) and 4Å MS (10 g) in 500 mL of anhydrous MeCN, followed by addition of template **21**[3] (639 mg, 1.05 mmol, 1.05 equiv). The resulting suspension was stirred for 15 hours at 40°C under a N_2 atmosphere and then filtered over celite and concentrated *in vacuo*. The crude product was dry-loaded onto silica and purified by column chromatography (PE/EtOAc 19:1 → 9:1) to give diester **22b** (936 mg, 77%) as a colorless oil.

R_f=0.47 (PE/EtOAc 8:2); ^{1}H NMR (500 MHz, $CDCl_3$): complex, see figure S13; ^{13}C NMR (125 MHz, $CDCl_3$): δ=169.1, 168.4, 167.8, 167.4, 163.5, 163.3, 160.8, 160.6, 160.5, 150.3, 150.2, 139.2, 139.1, 138.6, 137.6, 134.5, 132.5, 132.4, 131.4, 130.9, 130.1, 120.1, 119.8, 119.7, 115.3, 115.2, 115.1, 114.2, 112.5, 112.3, 112.2, 109.6, 109.0, 108.9, 84.6, 75.8, 68.2, 68.1, 55.7, 55.7, 52.9, 52.8, 52.6, 52.0, 44.9, 44.6, 44.1, 37.6, 37.1, 37.0, 33.8, 29.7, 29.6, 29.6, 29.5, 29.5, 29.4, 29.4, 29.3, 29.2, 29.1, 29.1, 29.0, 28.9, 28.7, 28.7, 28.5, 28.4, 27.7, 27.6, 27.4, 26.9, 26.7, 24.3, 24.0, 23.8, 18.4; IR (neat): $\tilde{v}$=3308, 3075, 2926, 2854, 2105, 1741, 1637, 1620, 1584, 1534, 1508, 1438, 1349, 1257, 1236, 1188, 1154, 1111, 1099, 1063, 1034, 997, 950, 911, 830, 769, 744, 723, 687, 632, 557, 501, 467; HRMS (FD$^+$): *m/z* calcd for $C_{71}H_{96}N_8O_{10}^+$ ($M^{\cdot+}$) 1220.7244; found: 1220.7266;

Macrocycle (23b):

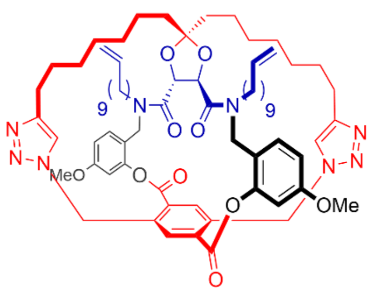

Azidoalkyne **22b** (936 mg, 766 µmol, 1 equiv) and TBTA (122 mg, 230 µmol, 0.3 equiv) were dissolved in 770 mL of CH_2Cl_2 under a N_2 atmosphere. The solution was bubbled with N_2 under vigorous stirring for 2 hours, then $Cu(MeCN)_4BF_4$ (72.3 mg, 230 µmol, 0.3 equiv) was added and the mixture was heated under reflux for 3 days. Conversion was monitored by IR. Once the reaction was complete, the crude mixture was cooled to room temperature, concentrated *in vacuo* and dry-loaded onto silica. The product was purified by column chromatography (PE/EtOAc 7:3 → 6:4) to give macrocycle **23b** (222 mg, 24%) as a colorless glass.

R_f=0.39 (PE/EtOAc 1:1); ^{1}H NMR (400 MHz, $CDCl_3$): δ=8.37 (s, 2H), 8.01 (s, 2H), 7.19 (d, *J*=8 Hz, 2H), 6.85 (dd, *J*=8, 3 Hz, 2H), 6.77 (d, *J*=3 Hz, 2H), 6.39 (d, *J*=16 Hz, 2H), 5.82 (m, 2H), 5.65 (d, *J*=13 Hz, 2H), 5.62 (d, *J*=15 Hz, 2H), 4.54 (s, 2H), 3.89 (s, 6H), 3.59 (d, *J*=15 Hz, 2H), 3.20 (m, 2H), 2.89–2.65 (m, 4H), 2.55 (m, 2H), 2.05 (q, *J*=7 Hz), 4H), 1.76–0.98 (m, 52H); ^{13}C NMR (100 MHz, $CDCl_3$): δ=168.0, 163.6, 160.7, 150.3, 148.5, 139.1, 138.1, 132.2, 131.4, 130.0, 123.6, 119.3, 116.0, 114.2, 113.1, 108.5, 76.1, 55.7, 49.6, 44.5, 36.1, 33.8, 31.9, 31.4, 30.2, 29.7, 29.4, 29.4, 29.3, 29.3, 29.2, 29.1, 28.9, 28.3, 28.3, 28.2, 27.7, 27.1, 26.7, 25.8, 22.7, 21.5, 14.1; IR (neat): $\tilde{v}$=2924, 2853, 1735, 1621, 1583, 1507, 1456, 1438, 1358, 1296, 1242, 1188, 1154, 1111, 1099, 1067, 1049, 1030, 952, 908, 890, 806, 762, 729, 695, 645, 627, 463; HRMS (ESI$^+$): m/z calcd for $C_{71}H_{97}N_8O_{10}^+$ $[M+H]^+$ 1221.7322; found: 1221.7172;

Double macrocycle (24b):

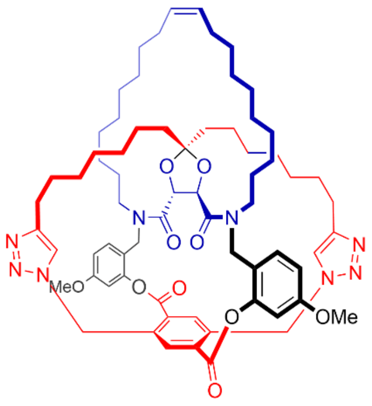

Dialkene **23b** (110 mg, 90.1 µmol, 1 equiv) was dissolved in 90 mL of CH_2Cl_2 under a N_2 atmosphere. The solution was bubbled with N_2 under vigorous stirring for 1 hour, then Grubbs 2nd generation catalyst (15.3 mg, 18.0 µmol, 0.2 equiv) was added and bubbling was continued for 10 minutes before warming the mixture at 40°C for 16 hours. Conversion was monitored by ^{1}H-NMR. Once the reaction was complete, the crude mixture was cooled to room temperature, concentrated *in vacuo* and dry-loaded onto silica. The product was purified by column chromatography using an automatic column machine (CH_2Cl_2/EtOAc 9:1 → 7:3 gradient) to give double macrocycle **24b** (61.5 mg, 57%) as an E/Z mixture, as a slightly pink crystalline solid.

R_f=0.26 (CH_2Cl_2/EtOAc 7:3); ^{1}H NMR (400 MHz, $CDCl_3$): δ=8.34 (s, 2H), 8.00 (s, 1.5H), 7.88 (s, 0.5H), 7.20 (d, *J*=8 Hz, 2H), 6.86 (dd, *J*=8, 3 Hz, 2H), 6.78 (d, *J*=3 Hz, 2H), 6.40 (d, *J*=16 Hz, 2H), 5.65 (d, *J*=16 Hz, 2H), 5.40–5.27 (m, 2H), 4.70–4.53 (m, 2H), 3.89 (s, 6H), 3.60 (d, *J*=15 Hz, 2H), 3.26 (m, 2H), 2.80 (m, 4H), 2.52 (m, 2H), 1.95 (m, 4H), 1.73–1.53 (m, 8H), 1.53–1.02 (m, 52H); ^{13}C NMR (100 MHz, $CDCl_3$): δ=168.0, 167.9, 167.7, 167.6, 164.0, 163.8, 163.7, 163.6, 160.8, 160.7, 150.5, 150.3, 150.3, 148.4, 138.1, 132.4, 132.2, 131.5, 130.4, 130.1, 129.9, 129.6, 123.6, 119.5, 119.4, 119.4, 119.3, 116.0, 115.9, 115.8, 114.2, 113.1, 108.6, 76.5, 55.7, 49.7, 44.5, 44.0, 43.8, 37.6, 36.9, 36.7, 33.8, 33.7, 33.0, 32.6, 31.9, 30.3, 30.1, 30.0, 29.7, 29.7, 29.7, 29.5, 29.4, 29.4, 29.1, 29.1, 28.9, 28.9, 28.6, 28.6, 28.3, 28.2, 28.2, 27.9, 27.8, 27.1, 26.9, 26.7, 25.9, 25.8, 25.4, 24.8, 22.7, 21.5, 21.4, 14.1; IR (neat): $\tilde{v}$=2924, 2853, 1737, 1623, 1583, 1508, 1457, 1440, 1359, 1297, 1244, 1188, 1155, 1112, 1101, 1068, 1049, 1032, 967, 952, 919, 890, 826, 763, 732; HRMS (ESI$^+$): *m/z* calcd for $C_{69}H_{93}N_8O_{10}^+$ $[M+H]^+$ 1193.7009; found: 1193.6945;

Double macrocycle (24b-H_2):

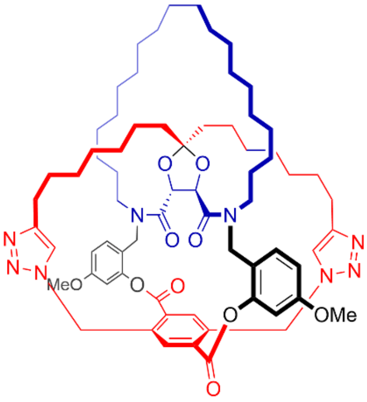

Alkene **24b** (61.5 mg, 51.5 µmol, 1 equiv) was dissolved in 12.5 mL of EtOAc/MeOH/THF 2:2:1 under a N_2 atmosphere. After addition of 10%w/w Pd/C (11.0 mg, 10.3 µmol, 0.2 equiv), the mixture was bubbled with H_2 for 15 minutes and then stirred under a H_2 atmosphere at 45°C for 19 hours. The crude mixture was then cooled to room temperature, filtered over celite with EtOAc and concentrated *in vacuo*. The crude product was dry-loaded onto silica and purified by column chromatography (CH_2Cl_2/EtOAc 9:1 → 8:2 → 7:3 → 4:6) to give alkane **24b-H₂** (53.3 mg, 87%) as a white powder.
R_f=0.33 (CH_2Cl_2/EtOAc 7:3); ^{1}H NMR (400 MHz, $CDCl_3$): δ=8.38–8.29 (m, 2H), 8.02–7.95 (m, 2H), 7.20 (d, *J*=8 Hz, 2H), 6.86 (dd, *J*=8, 3 Hz, 2H), 6.77 (d, *J*=3 Hz, 2H), 6.39 (d, *J*=16 Hz, 2H), 5.65 (d, *J*=16 Hz, 2H), 5.64 (d, *J*=15 Hz, 2H), 4.67–4.53 (m, 2H), 3.89 (s, 6H), 3.59 (d, *J*=15 Hz, 2H), 3.26 (m, 2H), 2.81 (m, 4H), 2.51 (m, 2H), 1.74–1.54 (m, 8H), 1.50–1.14 (m, 60H); ^{13}C NMR (100 MHz, $CDCl_3$): δ=168.0, 167.7, 164.0, 163.7, 163.6, 160.8, 160.7, 150.4, 150.3, 148.3, 138.1, 132.4, 132.2, 131.6, 131.5, 130.1, 129.9, 129.6, 123.6, 123.5, 119.4, 119.3, 119.3, 115.9, 115.8, 113.1, 113.0, 108.6, 76.5, 55.7, 49.9, 49.7, 44.5, 44.0, 43.8, 37.6, 36.9, 33.0, 31.9, 30.0, 29.8, 29.7, 29.5, 29.4, 29.4, 29.3, 29.2, 29.2, 29.2, 29.1, 28.8, 28.6, 28.2, 28.1, 27.8, 27.1, 25.9, 25.8, 22.7, 21.6, 21.4, 21.1, 14.2; IR (neat): $\tilde{\nu}$=2923, 2853, 1737, 1623, 1583, 1553, 1508, 1457, 1439, 1358, 1298, 1188, 1155, 1112, 1101, 1068, 1049, 1032, 951, 918, 890, 826, 762, 732, 645, 626, 580, 544, 462, 427; HRMS (FD$^+$): *m/z* calcd for $C_{69}H_{94}N_8O_{10}^+$ ($M^{\cdot+}$) 1194.7087; found: 1194.7108;

Precatenane (9b):

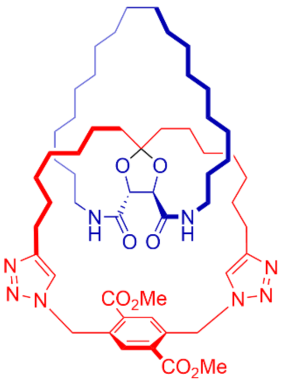

Bislactone **24b-H₂** (53.3 mg, 44.6 µmol, 1 equiv) was dissolved in 9 mL of anhydrous THF/MeOH 1:1 and K_2CO_3 (1.54 g, 11.1 mmol, 250 equiv) was added. The suspension was vigorously stirred under a N_2 atmosphere for 6 hours, then the solution was neutralized with several drops of AcOH and quickly concentrated *in vacuo* without going to dryness. The slurry was diluted with 20 mL of EtOAc and 20 mL of H_2O was added. The aqueous phase was extracted 2 × 20 mL EtOAc and the reunited organic phases were dried over $MgSO_4$ and concentrated *in vacuo*. The oil obtained was dissolved in a solution of triethylsilane (71.2 µL, 446 µmol, 10 equiv) in 2.1 mL of TFA and stirred for 16 hours at room temperature. The reaction mixture was then diluted and concentrated 2 × 6 mL of toluene. The crude product was purified by column chromatography using an automatic column machine (MeOH in EtOAc 1% → 15% gradient) to give precatenane **9b** (19.3 mg, 44%) as a colorless glass.
R_f=0.39 (MeOH in EtOAc 10%); ^{1}H NMR (400 MHz, $CDCl_3$): δ=8.32 (s, 1H), 8.30 (s, 1H), 7.59 (s, 1H), 7.56 (s, 1H), 6.55–6.50 (m, 2H), 6.18 (d, *J*=14 Hz, 1H), 6.16 (d, *J*=14 Hz, 1H), 5.55 (d, *J*=14 Hz, 2H), 5.02–4.85 (m, 2H), 3.99 (s, 3H), 3.98 (s, 3H), 3.54 (m, 2H), 2.94 (m, 2H), 2.79–2.54 (m, 4H), 1.72–1.50 (m, 8H), 1.49–1.01 (m, 34H), 1.03–0.72 (m, 16H), 0.72–0.50 (m, 2H); ^{13}C NMR (100 MHz, $CDCl_3$): δ= 170.2, 170.1, 166.0, 165.9, 148.5, 148.2, 136.9, 136.8, 134.7, 134.5, 132.8, 132.7, 121.3, 116.2, 76.2, 53.0, 50.1, 50.0, 38.9, 38.8, 37.6, 37.6, 33.8, 31.9, 30.0, 29.9, 29.8, 29.8, 29.7, 29.7, 29.6, 29.6, 29.6, 29.5, 29.5, 29.4, 29.4, 29.4, 29.3, 29.2, 29.2, 28.9, 28.9, 28.8, 28.7, 28.7, 28.6, 28.5, 28.5, 28.4, 28.2, 27.1, 27.0, 27.0, 25.7, 25.6, 25.5, 24.8, 24.8, 22.7, 14.1; IR (neat): $\tilde{\nu}$=2926, 2854, 1721, 1682, 1528, 1461, 1436, 1353, 1294, 1265, 1204, 1106, 1047, 968, 929, 808, 761, 727; HRMS (FD$^+$): *m/z* calcd for $C_{55}H_{86}N_8O_8Na^+$ $[M+Na]^+$ 1009.6461; found: 1009.6598;

[2]catenane (10b):

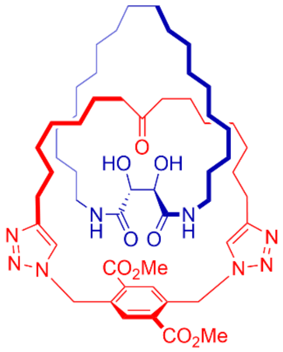

Precatenane **9b** (10.2 mg, 10.3 µmol, 1 equiv) was dissolved in 5 mL of 33% v/v H_2SO_4 in AcOH. After adding 0.7 mL of H_2O the mixture was warmed to 30°C and stirred for 3 days. The resulting mixture was cooled to 0°C and diluted in 50 mL of H_2O and 20 mL of CH_2Cl_2. The aqueous layer was extracted 2 × 20 mL CH_2Cl_2 and the reunited organic phases were washed with 50 mL of saturated aqueous $NaHCO_3$, dried over $MgSO_4$ and concentrated *in vacuo*. The crude product was dry loaded onto silica and purified by column chromatography (EtOAc/MeOH 19:1 → 18:1 → 17:1) to give [2]catenane **10b** (6.2 mg, 60%) as a colorless glass.
R_f=0.23 (EtOAc/MeOH 9:1); ^{1}H NMR (500 MHz, $(CD_3)_2SO$): δ=8.13 (m, 2H), 7.72–7.62 (m, 2H), 7.42–7.24 (m, 2H), 6.10 (d, *J*=14 Hz, 2H), 5.60 (d, *J*=14 Hz, 2H), 5.52–5.59 (m, 2H), 4.19 (m, 2H), 3.92 (s, 6H), 3.18–2.93 (m, 4H), 2.55 (m, 4H), 2.27 (m, 4H), 1.50–1.35 (m, 8H), 1.34–1.19 (m, 16H), 1.17–1.09 (m, 4H), 1.07–0.99 (m, 4H), 0.96–0.81 (m, 24H); ^{13}C NMR (125 MHz, $(CD_3)_2SO$): δ=210.5, 171.9, 166.36, 166.34, 147.42, 147.39, 136.7, 134.3, 133.45, 133.42, 121.83, 121.80, 72.8, 53.3, 49.9, 42.5, 38.60, 38.47, 31.6, 30.3, 29.8, 29.7, 29.4, 29.3, 29.2, 29.1, 29.0, 28.9, 28.7, 28.5, 27.3, 25.7, 23.7, 22.5, 14.4.; IR (neat): $\tilde{\nu}$=3359 (br), 2925, 2853, 1719, 1659, 1537, 1461, 1436, 1364, 1295, 1269, 1190, 1157, 1107, 1013, 969, 812, 549 (br); HRMS (FD$^+$): *m/z* calcd for $C_{55}H_{89}N_8O_9^+$ $[M+H]^+$ 1005.6747; found: 1005.6757;

Trivial macrocycle (25b):

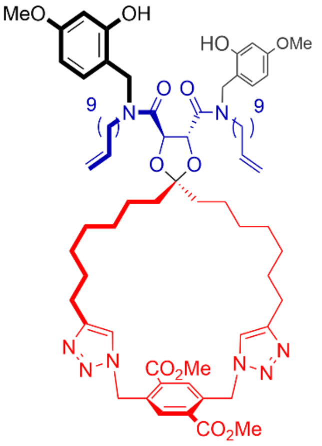

Macrocycle **23b** (211 mg, 172 µmol, 1 equiv) was dissolved in 34 mL of anhydrous THF/MeOH 1:1 and K_2CO_3 (5.96 g, 43.1 mmol, 250 equiv) was added. The suspension was vigorously stirred under a N_2 atmosphere for 5 hours, then the solution was neutralized with several drops of AcOH and quickly concentrated *in vacuo* without going to dryness. The slurry was diluted with 100 mL of EtOAc and 100 mL of H_2O was added. The aqueous phase was extracted 2 × 50 mL EtOAc and the reunited organic phases were washed with brine, dried over $MgSO_4$ and concentrated *in vacuo*. The obtained oil was dissolved in 5.7 mL of anhydrous DMSO and heated to 120°C for 5 days under a N_2 atmosphere. The resulting solution was diluted with 6 mL of H_2O and the solvents were removed by freeze drying. The crude product was dry loaded onto silica and purified by column chromatography (EtOAc/PE 1:1 → 2:1 → 3:1) to give trivial macrocycle **25b** (138 mg, 62%) as a colorless glass.
R_f=0.42 (EtOAc/PE 3:1); ^{1}H NMR (400 MHz, $CDCl_3$): δ=9.43 (s, 2H), 8.01 (s, 2H), 7.46 (s, 2H), 6.99 (d, J=8 Hz, 2H), 6.47 (s, 2H), 6.39 (d, J=11 Hz, 2H), 5.91 (s, 4H), 5.83 (m, 2H), 5.17 (s, 2H), 5.00 (d, J=17 Hz, 2H), 4.94 (d, J=10 Hz, 2H), 4.41 (d, J=15 Hz, 2H), 4.43 (d, J=15 Hz, 2H), 3.92 (s, 6H), 3.77 (s, 6H), 3.53–3.32 (m, 4H), 2.68 (t, J=8 Hz, 4H), 2.10–1.98 (m, 4H), 1.75–1.65 (m, 4H), 1.63–1.49 (m, 8H), 1.44–1.37 (m, 4H), 1.36–1.35 (m, 24H), 1.24–1.14 (m, 12H); ^{13}C NMR (125 MHz, $CDCl_3$): δ=170.0, 165.8, 161.4, 157.4, 148.6, 139.1, 137.0, 133.9, 132.4, 132.1, 121.6, 116.2, 114.3, 114.2, 105.8, 102.5, 75.3, 55.2, 52.9, 50.8, 47.2, 46.1, 36.5, 33.8, 29.5, 29.5, 29.4, 29.3, 29.3, 29.1, 29.0, 28.9, 28.6, 28.2, 26.9, 25.4, 23.9; IR (neat): $\tilde{\nu}$=2925, 2853, 1721, 1617, 1521, 1461, 1435, 1358, 1291, 1256, 1202, 1162, 1105, 1038, 964, 842, 731; HRMS (FD$^+$): *m/z* calcd for $C_{73}H_{105}N_8O_{12}^+$ [M+H]$^+$ 1285.7846; found: 1285.7814;

Trivial bismacrocycle (26b):

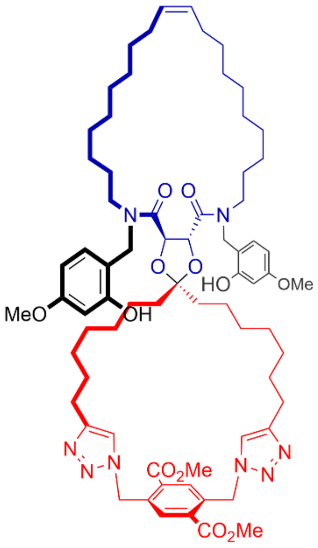

Trivial macrocycle **25b** (138 mg, 107 µmol, 1 equiv) was dissolved in 100 mL of CH_2Cl_2 under a N_2 atmosphere. The solution was bubbled with N_2 under vigorous stirring for 1 hour, then Grubbs 2nd generation catalyst (18.2 mg, 21.4 µmol, 0.2 equiv) was added and bubbling was continued for 10 minutes before warming the mixture at 40°C for 16 hours. The crude mixture was then cooled to room temperature, concentrated *in vacuo* and dry-loaded onto silica. The product was purified by column chromatography (EtOAc/PE 6:4 → 7:3 → 9:1) to give trivial bismacrocycle **26b** (92.1 mg, 68%) as an E/Z mixture, as a slightly brown glass.
R_f=0.31 (EtOAc/PE 7:3); ^{1}H NMR (400 MHz, $CDCl_3$): δ= complex, see figure S30; ^{13}C NMR (125 MHz, $CDCl_3$): δ=171.1, 170.9, 170.8, 170.5, 167.4, 166.0, 165.8, 165.8, 161.5, 161.4, 160.6, 157.5, 157.4, 156.4, 148.8, 148.7, 148.7, 148.6, 139.1, 137.1, 136.9, 134.1, 133.8, 132.7, 132.5, 132.4, 132.3, 132.2, 132.1, 130.61, 130.57, 130.5, 130.02, 129.96, 129.62, 129.58, 121.9, 121.5, 121.4, 121.0, 120.9, 116.8, 116.43, 116.37, 114.55, 114.52, 114.32, 114.29, 114.2, 114.2, 105.9, 105.8, 105.3, 102.6, 102.5, 102.1, 77.2, 74.7, 60.4, 55.2, 55.2, 53.0, 52.9, 52.9, 50.9, 50.7, 50.0, 47.1, 46.9, 46.3, 46.2, 46.1, 45.2, 37.9, 36.6, 36.55, 36.45, 33.8, 32.6, 32.2, 29.70, 29.66, 29.6, 29.52, 29.48, 29.40, 29.37, 29.3, 29.22, 29.16, 29.1, 29.00, 28.97, 28.9, 28.8, 28.8, 28.7, 28.6, 28.5, 28.5, 28.3, 28.2, 28.1, 28.0, 27.7, 27.0, 26.9, 26.8, 26.7, 26.6, 26.1, 26.0, 25.6, 25.5, 25.4, 24.0, 23.7, 23.4, 23.2, 22.7, 21.1, 14.2; IR (neat): $\tilde{\nu}$=3136, 2925, 2853, 1721, 1618, 1521, 1507, 1460, 1436, 1357, 1291, 1258, 1202, 1162, 1106, 1037, 965, 911, 842, 799, 731, 645; HRMS (FD$^+$): *m/z* calcd for $C_{71}H_{101}N_8O_{12}^+$ [M+H]$^+$ 1257.7533; found: 1257.7519;

Trivial bismacrocycle (26b-H_2):

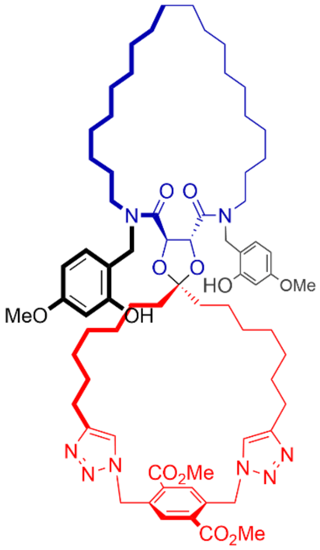

Alkene **26b** (80.4 mg, 63.9 µmol, 1 equiv) was dissolved in 10 mL of EtOAc/MeOH 1:1 under a N_2 atmosphere. After addition of 10%w/w Pd/C (13.6 mg, 12.8 µmol, 0.2 equiv), the mixture was bubbled with H_2 for 10 minutes and then stirred under a H_2 atmosphere at 45°C for 19 hours. The crude mixture was then cooled to room temperature, filtered over celite with EtOAc and concentrated *in vacuo*. The crude product was dry-loaded onto silica and purified by column chromatography (EtOAc/PE 7:3 → 9:1) to give alkane **26b-H_2** (71.4 mg, 89%) as a colorless glass. R_f=0.39 (EtOAc/PE 7:3); ^{1}H NMR (400 MHz, $CDCl_3$): δ= complex, see figure S32; ^{13}C NMR (125 MHz, $CDCl_3$): δ=170.44, 170.19, 170.02, 167.42, 165.97, 165.83, 165.78, 161.42, 160.58, 157.45, 157.40, 157.38, 156.35, 148.76, 148.62, 137.04, 137.02, 136.84, 134.07, 134.04, 133.82, 132.47, 132.42, 132.35, 132.20, 132.12, 130.30, 121.88, 121.56, 121.46, 116.26, 114.55, 114.34, 114.30, 105.90, 105.73, 105.68, 105.21, 102.59, 102.53, 102.03, 77.28, 76.63, 74.59, 60.40, 55.23, 53.01, 52.88, 52.86, 50.88, 50.72, 47.17, 46.97, 46.89, 46.35, 46.07, 45.12, 40.91, 38.00, 36.57, 36.44, 33.95, 31.92, 29.86, 29.70, 29.66, 29.63, 29.60, 29.58, 29.55, 29.48, 29.41, 29.36, 29.30, 29.27, 29.25, 29.18, 29.15, 29.06, 29.04, 29.01, 28.97, 28.91, 28.83, 28.79, 28.76, 28.66, 28.62, 28.58, 28.52, 28.47, 28.40, 28.35, 28.24, 27.79, 26.87, 26.85, 26.79, 26.60, 25.89, 25.59, 25.54, 25.49, 25.38, 24.00, 23.91, 23.70, 23.46, 22.70, 21.05, 20.85, 17.52, 17.30, 14.13; IR (neat): $\tilde{v}$=3138, 2924, 2853, 1721, 1618, 1507, 1460, 1436, 1360, 1291, 1257, 1202, 1162, 1106, 1038, 965, 912, 842, 786, 731, 645; HRMS (ESI$^+$) *m/z* calcd for $C_{71}H_{103}N_8O_{12}^+$ [M+H]$^+$ 1259.7690, found 1259.7664.

Cleavage of trivial bismacrocycle (26b-H_2):

Trivial bismacrocycle **26b-H_2** (65.5 mg, 52.0 µmol, 1 equiv) was dissolved in a solution of triethylsilane (83 µL, 520 µmol, 10 equiv) in 2.0 mL of TFA and stirred for 16 hours at room temperature. The reaction mixture was then diluted and concentrated 2 × 10 mL of toluene to give a mixture of bismacrocycle **27b** and separate macrocycles **28b** and **29**. This was dissolved in 2.5 mL of MeOH to which 2.5 mL of 32% aqueous HCl was added and then stirred for 5 hours. The reaction mixture was then carefully quenched with 100 mL of saturated aqueous $NaHCO_3$ and extracted 3 × 50 mL EtOAc. The reunited organic phases were washed with brine, dried and concentrated *in vacuo*. The crude product was purified by column chromatography using an automatic column machine (MeOH in CH_2Cl_2 0% → 20% gradient) to give macrocycles **28b** and **29** (23.5 mg, 45% ca.) as a mixture which could not be separated quantitatively.

Macrocyclic ketone (28b):

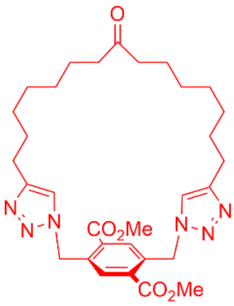

R_f=0.31 (CH_2Cl_2/MeOH 19:1); ^{1}H NMR (400 MHz, $CDCl_3$): δ=7.96 (s, 2H), 7.49 (s, 2H), 5.93 (s, 4H), 3.94 (s, 6H), 2.72 (t, J=7.4 Hz, 4H), 2.33 (t, J=6.9 Hz, 4H), 1.64 (m, 4H), 1.51 (m, 4H), 1.38–1.24 (m, 12H); ^{13}C NMR (125 MHz, $CDCl_3$): δ=211.52, 165.85, 148.51, 137.13, 133.43, 132.27, 121.68, 52.88, 50.83, 42.56, 28.94, 28.78, 28.55, 28.40, 25.52, 23.62; IR (neat): $\tilde{v}$=2924, 2853, 1731, 1717, 1703, 1461, 1435, 1288, 1249, 1203, 1112, 1051; HRMS (FD$^+$) *m/z* calcd for $C_{31}H_{43}N_6O_5^+$ [M+H]$^+$ 579.3289, found 579.3231.

Macrocyclic diol (29):

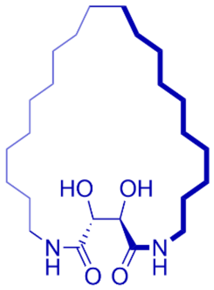

R_f=0.26 (CH_2Cl_2/MeOH 19:1); ^{1}H NMR (400 MHz, $CDCl_3$): δ=7.11 (s, 2H), 5.63 (s, 2H), 4.26 (s, 2H), 3.40 (m, 2H), 3.14 (m, 2H), 1.67–1.44 (m, 8H), 1.36–1.25 (m, 28H); ^{13}C NMR (125 MHz, $CDCl_3$): δ=174.1, 69.9, 39.1, 29.5, 29.1, 29.0, 28.7, 28.4, 28.1, 27.6, 27.5, 26.6; IR (neat): $\tilde{v}$=3368, 3299, 2924, 2853, 1651, 1618, 1535, 1457, 1438, 1411, 1277, 1111, 1065. HRMS (FD$^+$) *m/z* calcd for $C_{24}H_{46}N_2O_4Na^+$ $[M+Na]^+$ 449.3350, found 449.3357.

Macrocycle (23a):

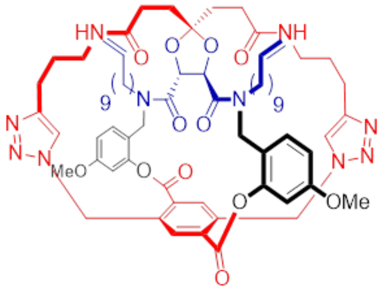

Azidoalkyne **22a**[1] (1.17 g, 0.939 mmol, 1 equiv) and TBTA (93.1 mg, 175 µmol, 0.2 equiv) were dissolved in 470 mL of CH_2Cl_2 under a N_2 atmosphere. The solution was bubbled with N_2 under vigorous stirring for 1 hour, then $Cu(MeCN)_4BF_4$ (59.1 mg, 189 µmol, 0.2 equiv) was added and the mixture was heated under reflux for 23 hours. Conversion was monitored by IR. Once the reaction was complete, the crude mixture was cooled to room temperature, concentrated *in vacuo* and dry-loaded onto silica. The product was purified by column chromatography using an automatic column machine (CH_2Cl_2/MeOH 43:7) to give macrocycle **23a** (1.10 g, 93%) as a colorless glass. ^{1}H NMR data matched those reported in literature.[1]

R_f=0.0.26–0.43 (CH_2Cl_2/MeOH 19:1); ^{1}H NMR (400 MHz, $CDCl_3$): δ= complex, see figure S46.

Unthreaded macrocycle (25a):

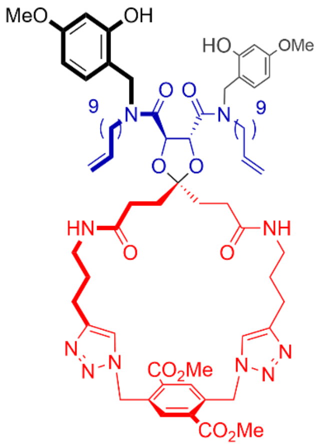

Bislactone **23a** (300 mg, 240 µmol, 1 equiv) was dissolved in 15 mL of anhydrous MeOH/THF 2:1 mixture and K_2CO_3 (7.07 g, 50 mmol, 210 equiv) was added. After stirring for 5 hours under a N_2 atmosphere at room temperature the reaction mixture was quenched with a few mL of AcOH, quickly concentrated and 50 mL EtOAc and 50 mL H_2O were added. The aqueous layer was then extracted 2×25mL of EtOAc. The reunited organic phases were washed with brine, dried over $MgSO_4$ and dry loaded onto silica. The product was purified by column chromatography using an automatic column machine (MeOH in EtOAc 1% → 20% gradient) to give unthreaded **25a** (107 mg, 34%) as a colorless glass.

R_f=0.0.18 (EtOAc/MeOH 9:1); ^{1}H NMR (400 MHz, $CDCl_3$): δ= complex, see figure S47; ^{13}C NMR (100 MHz, $CDCl_3$): δ=174.03, 172.76, 172.67, 169.80, 169.72, 169.67, 165.76, 165.67, 165.57, 161.44, 160.80, 157.97, 157.11, 156.80, 147.59, 139.14, 139.08, 137.03, 136.93, 136.69, 134.35, 133.49, 132.73, 132.54, 132.40, 132.26, 130.90, 121.89, 115.24, 115.07, 114.94, 114.21, 114.17, 114.14, 105.55, 104.02, 102.45, 102.35, 102.18, 75.75, 55.25, 55.19, 53.00, 52.97, 52.89, 50.97, 48.60, 46.82, 45.95, 39.00, 38.82, 38.52, 33.79, 32.83, 31.40, 30.69, 29.48, 29.46, 29.41, 29.27, 29.10, 29.02, 28.90, 28.30, 26.97, 26.77, 22.98, 22.63, 22.54; IR (neat): $\tilde{v}$=3271, 3077, 2925, 2853, 1720, 1617, 1551, 1522, 1508, 1434, 1352, 1290, 1258, 1201, 1163, 1104, 1037, 994, 964, 909, 844, 811, 779, 729, 644, 583; HRMS (FD$^+$): *m/z* calcd for $C_{71}H_{98}N_{10}O_{14}Na^+$ $[M+Na]^+$ 1337.7156; found: 1337.7253;

Trivial bismacrocycle (26a):

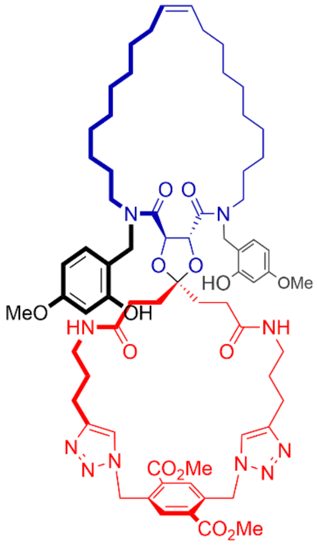

Dialkene **25a** (104 mg, 79.2 µmol, 1 equiv) was dissolved in 80 mL of CH_2Cl_2 under a N_2 atmosphere. The solution was bubbled with N_2 under vigorous stirring for 1 hour, then Grubbs 2nd generation catalyst (13.4 mg, 15.8 µmol, 0.2 equiv) was added and bubbling was continued for 10 minutes before warming the mixture at 40°C for 17 hours. Conversion was monitored by ^{1}H-NMR. Once the reaction was complete, the crude mixture was cooled to room temperature, concentrated *in vacuo* and dry-loaded onto silica. The product was purified by column chromatography (EtOAc/MeOH 19:1 → 18:2 → 17:3) to give double macrocycle **26a** (85.6 mg, 84%) as an E/Z mixture, as a white/brown solid.

R_f=0.13 (EtOAc/MeOH 9:1); ^{1}H NMR (400 MHz, $CDCl_3$): δ= complex, see figure S49; ^{13}C NMR (100 MHz, $CDCl_3$): δ=13C NMR (101 MHz, CDCl3) δ 174.10, 173.99, 173.20, 172.92, 172.90, 170.18, 170.02, 169.73, 165.75, 165.66, 165.57, 161.59, 161.55, 161.48, 160.97, 160.93, 158.21, 157.07, 156.63, 156.59, 147.96, 147.81, 147.71, 137.05, 136.82, 136.71, 135.49, 134.58, 133.65, 132.89, 132.52, 132.46, 132.30, 132.24, 131.26, 130.75, 130.72, 130.07, 130.04, 122.41, 121.89, 121.17, 115.77, 115.49, 115.43, 114.47, 113.93, 113.86, 105.70, 105.43, 103.97, 102.40, 102.29, 102.22, 75.58, 55.27, 55.24, 53.03, 52.98, 52.94, 51.12, 51.04, 47.94, 47.81, 46.39, 46.17, 45.84, 45.59, 44.04, 39.24, 38.78, 38.62, 34.95, 33.34, 33.22, 32.02, 31.88, 31.69, 31.55, 31.02, 30.96, 29.69, 29.52, 29.46, 29.38, 29.29, 29.26, 29.23, 29.21, 29.14, 29.02, 28.88, 28.81, 28.78, 28.66, 28.33, 28.19, 27.95, 27.91, 27.83, 27.13, 26.92, 26.85, 26.72, 26.49, 23.11, 22.69, 22.51, 14.12; IR (neat): $\tilde{v}$=3271, 3088, 2924, 2853, 1720, 1618, 1553, 1435, 1351, 1291, 1260, 1202, 1164, 1123, 1105, 1038, 965, 911, 846, 779, 730, 646, 581; HRMS (FD$^+$): *m/z* calcd for $C_{69}H_{95}N_{10}O_{14}^+$ $[M+H]^+$ 1287.7024; found: 1287.6935;

Trivial bismacrocycle (26a-H₂):

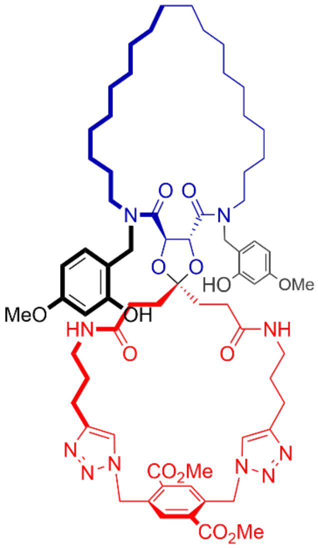

Alkene **26a** (85.6 mg, 66.5 µmol, 1 equiv) was dissolved in 4 mL of EtOAc/MeOH 1:1 under a N_2 atmosphere. After addition of 10%w/w Pd/C (14.2 mg, 13.3 µmol, 0.2 equiv), the mixture was bubbled with H_2 for 15 minutes and then stirred under a H_2 atmosphere at 50°C for 15 hours. The crude mixture was then cooled to room temperature, filtered over celite with EtOAc and concentrated *in vacuo*. The crude product was dry-loaded onto silica and purified by column chromatography (MeOH in EtOAc 5% → 10% → 15%) to give alkane **26a-H$_2$** (64.6 mg, 75%) as a white powder.

R_f=0.18 (EtOAc/MeOH 9:1); ^{1}H NMR (400 MHz, $CDCl_3$): δ= complex, see figure S51; ^{13}C NMR (100 MHz, $CDCl_3$): δ=13C NMR (75 MHz, CDCl3) δ 174.20, 172.88, 170.02, 165.66, 165.56, 161.55, 160.95, 158.20, 156.65, 147.96, 147.59, 136.80, 134.63, 132.90, 132.51, 132.31, 131.15, 122.51, 121.17, 115.40, 114.55, 113.96, 105.71, 103.95, 102.30, 75.55, 55.26, 53.01, 51.15, 48.24, 46.38, 45.87, 44.79, 44.76, 39.24, 29.71, 29.25, 29.08, 28.96, 28.71, 28.56, 28.12, 27.96, 27.74, 27.61, 27.53, 27.40, 26.95, 26.81, 23.09, 22.45, 14.14; IR (neat): $\tilde{v}$=3271, 3088, 2924, 2853, 1720, 1618, 1552, 1523, 1435, 1351, 1291, 1260, 1202, 1164, 1123, 1105, 1039, 965, 911, 845, 811, 778, 646; HRMS (FD$^+$): *m/z* calcd for $C_{69}H_{97}N_{10}O_{14}^+$ $[M+H]^+$ 1289.7180; found: 1289.7245;

Trivial bismacrocycle (27a):

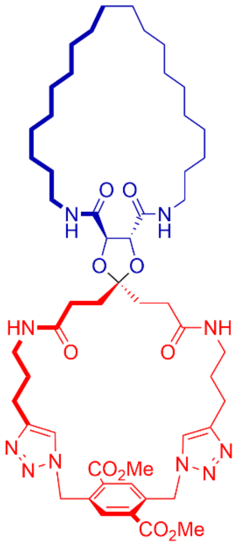

Tertiary amide **26a-H₂** (47.5 mg, 36.8 µmol, 1 equiv) was dissolved in a pre-mixed solution of Et_3SiH (58 µL, 0.37 mmol, 10 equiv) in 1.7 mL of TFA. After stirring at room temperature for 22 hours, 7 mL of toluene were carefully added to the mixture and the crude was concentrated *in vacuo*. The product was dry-loading onto silica and purified by column chromatography (MeOH in EtOAc 10% → 15% → 20%) to give secondary amide **27a** (32.8 mg, 88%) as a white powder.

R_f=0.07 (EtOAc/MeOH 9:1); ^{1}H NMR (400 MHz, $CDCl_3$): δ=7.93 (s, 2H), 7.77 (m , 2H), 7.52 (s, 2H), 6.71 (m, 2H), 5.97 (d, *J*=15 Hz, 2H) , 5.90 (d, *J*=15 Hz, 2H), 4.40 (s, 2H), 3.94 (s, 6H), 3.39–3.09 (m, 8H), 2.72 (t, *J*=7 Hz, 4H), 2.27 (m, 4H), 2.13–1.91 (m, 4H), 1.84 (m, 4H), 1.57 (m, 4H), 1.40–1.22 (m, 32H);

^{13}C NMR (100 MHz, $CDCl_3$): δ=13C NMR (101 MHz, CDCl3) δ 172.84, 169.30, 165.89, 147.46, 136.97, 133.69, 132.47, 121.97, 114.49, 78.09z, 52.96, 51.10, 39.43, 38.55, 32.53, 30.80, 29.70, 29.15, 29.00, 28.92, 28.88, 28.84, 28.74, 28.70, 28.62, 28.55, 28.51, 26.62, 22.60, 14.13; IR (neat): $\tilde{v}$=3280, 3081, 2925, 2854, 1721, 1654, 1547, 1437, 1350, 1296, 1258, 1202, 1125, 1107, 1051, 968, 919, 800, 732; HRMS (FD$^+$): *m/z* calcd for $C_{53}H_{80}N_{10}O_{10}Na^+$ $[M+Na]^+$ 1039.5951; found: 1039.5996;

NMR Spectra

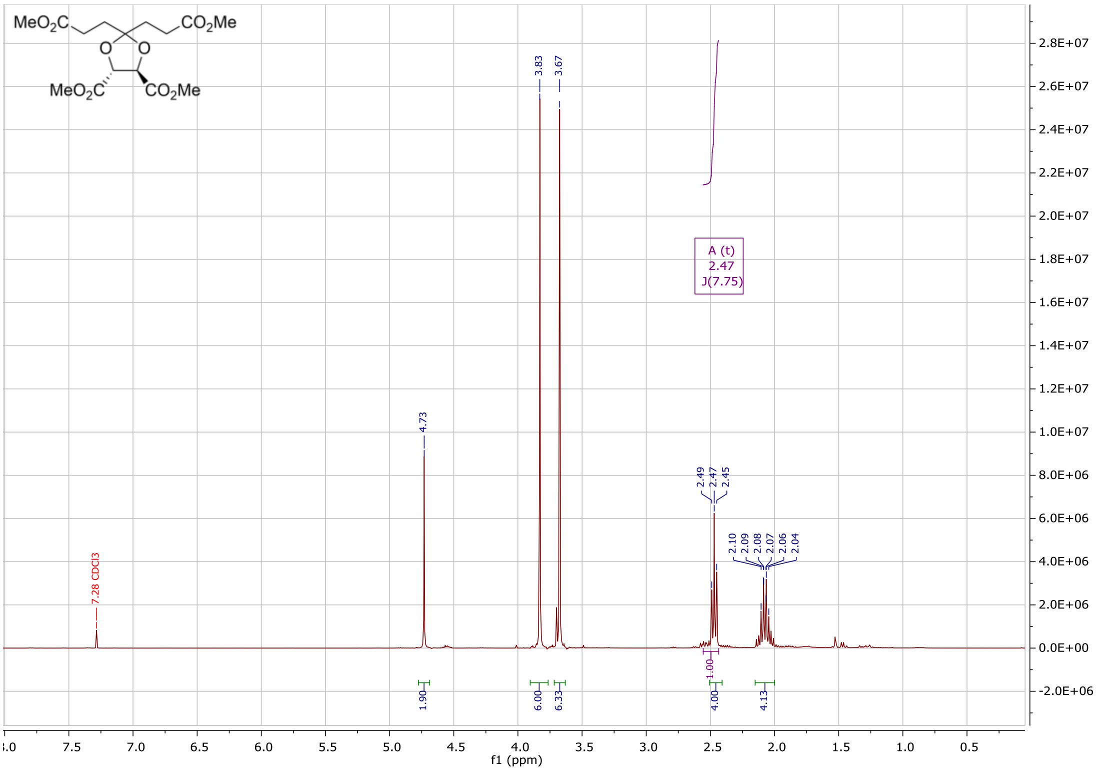

Figure S1. ^{1}H NMR, $CDCl_3$, compound **11**.

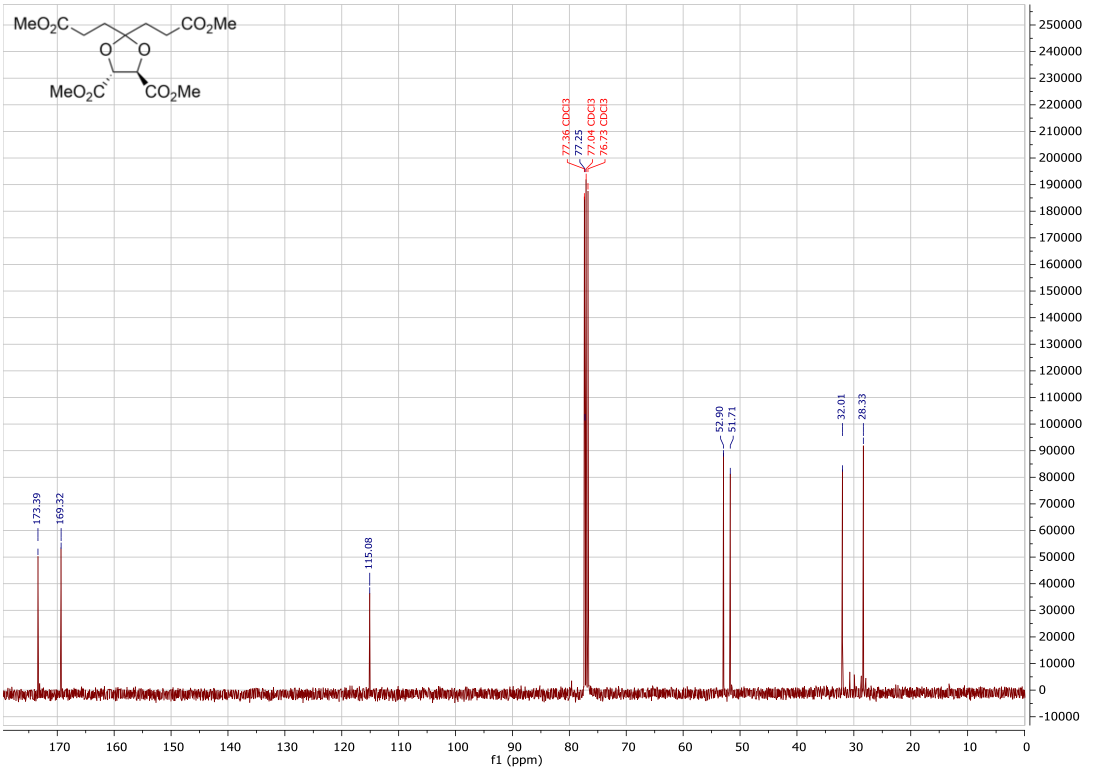

Figure S2. ^{13}C NMR, $CDCl_3$, compound **11**.

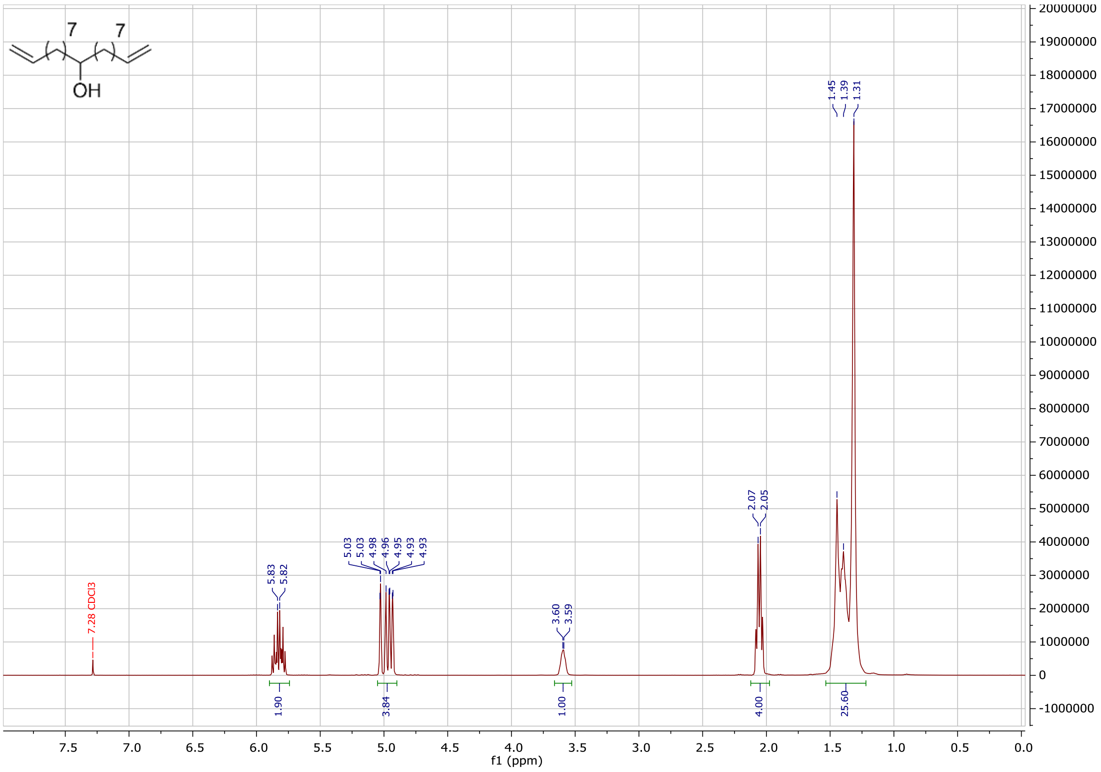

Figure S3. ^{1}H NMR, $CDCl_3$, compound **15**.

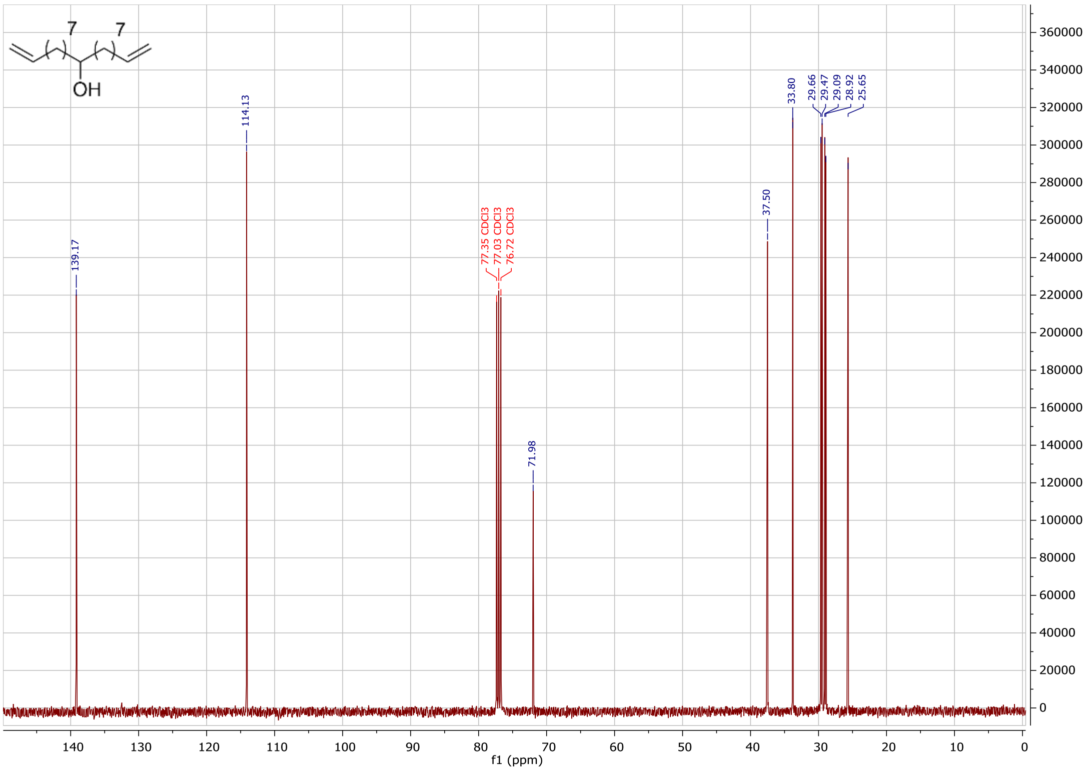

Figure S4. ^{13}C NMR, $CDCl_3$, compound **15**.

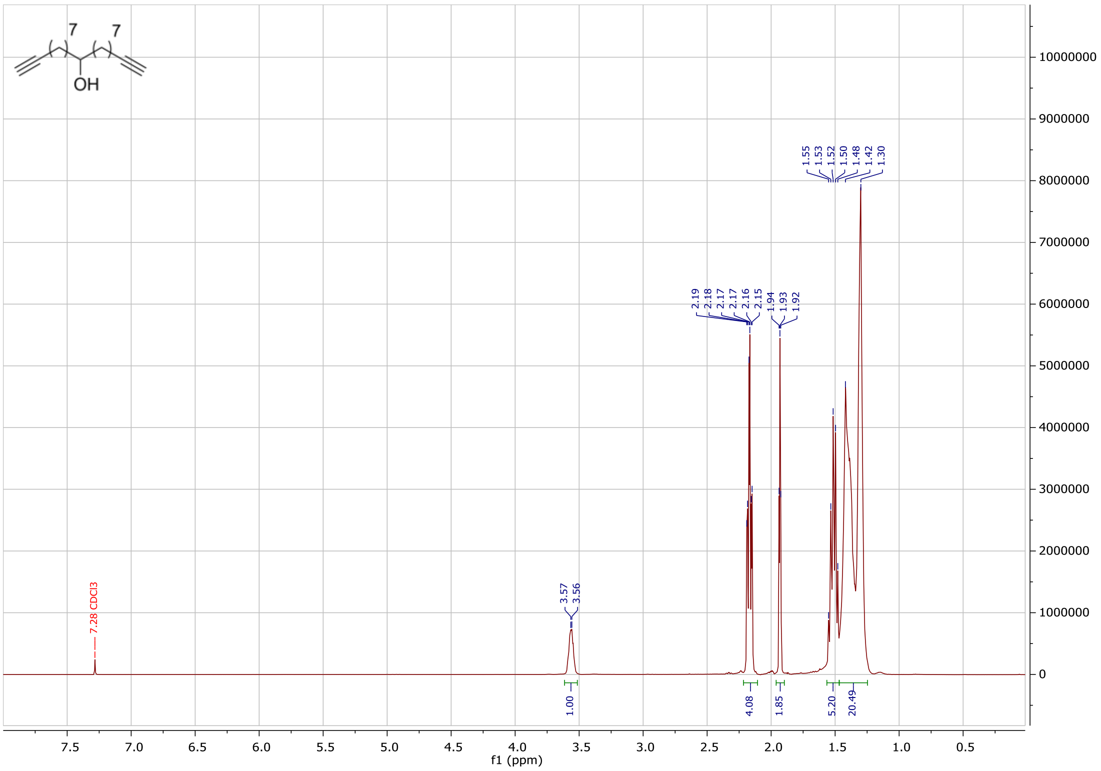

Figure S5. ^{1}H NMR, $CDCl_3$, compound **16**.

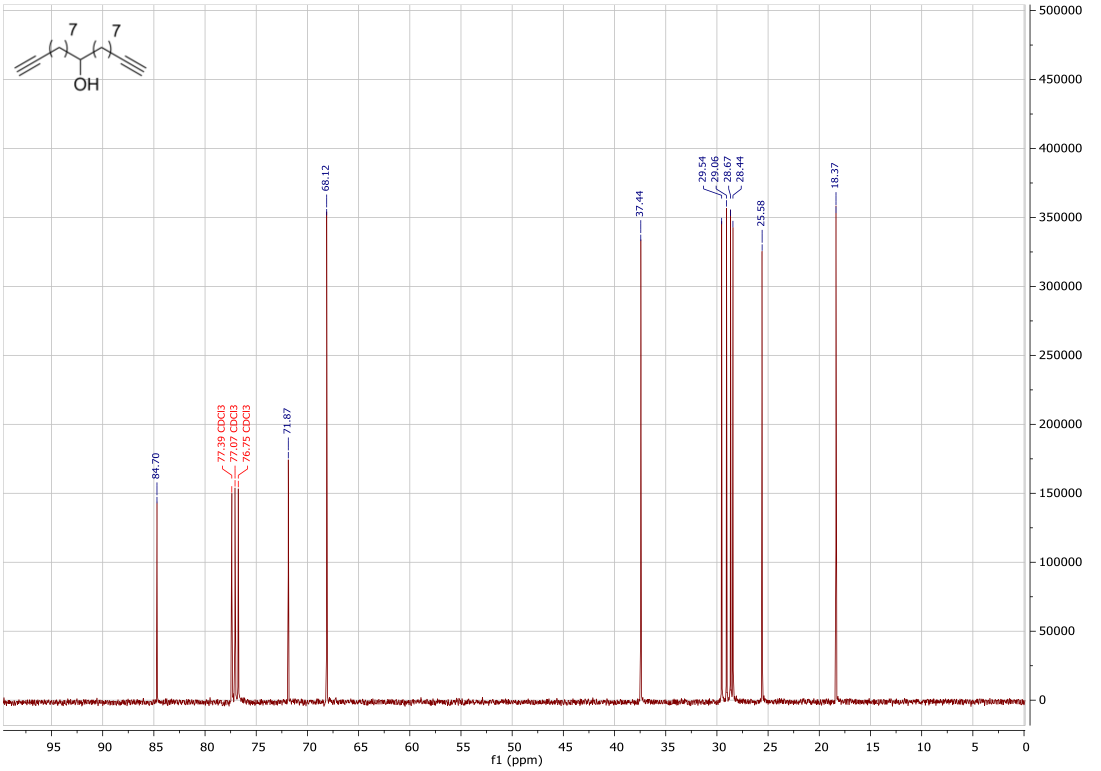

Figure S6. ^{13}C NMR, $CDCl_3$, compound **16**.

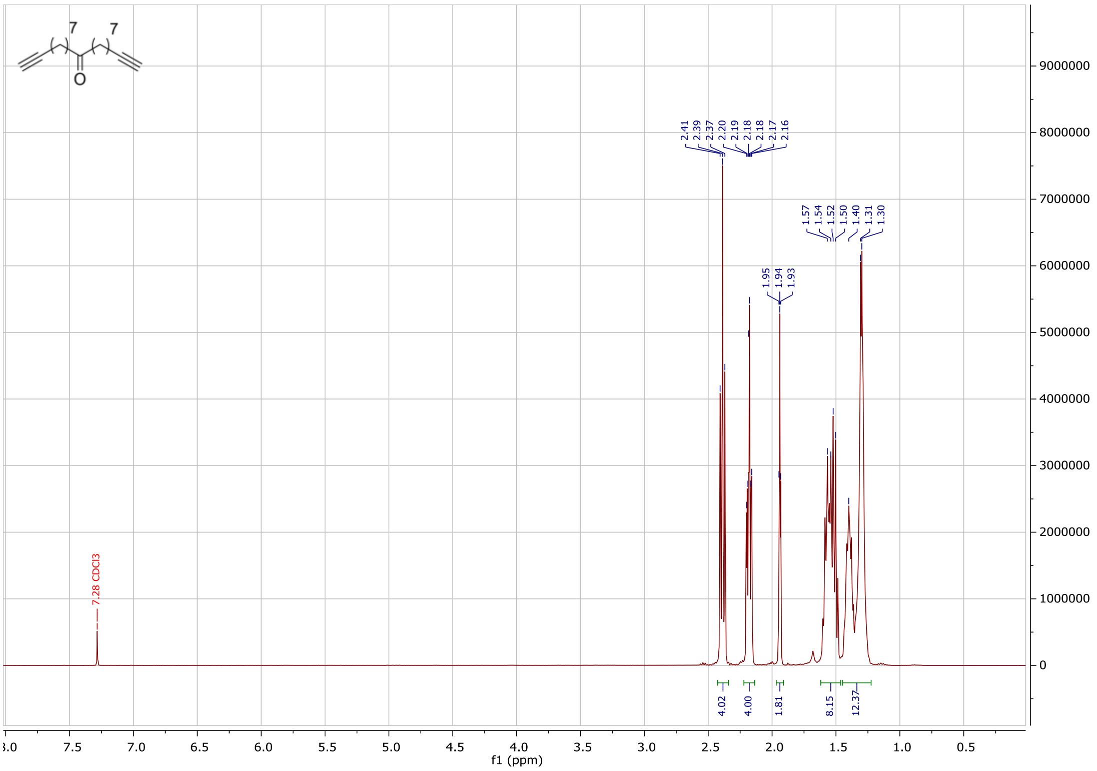

Figure S7. ^{1}H NMR, $CDCl_3$, compound **17**.

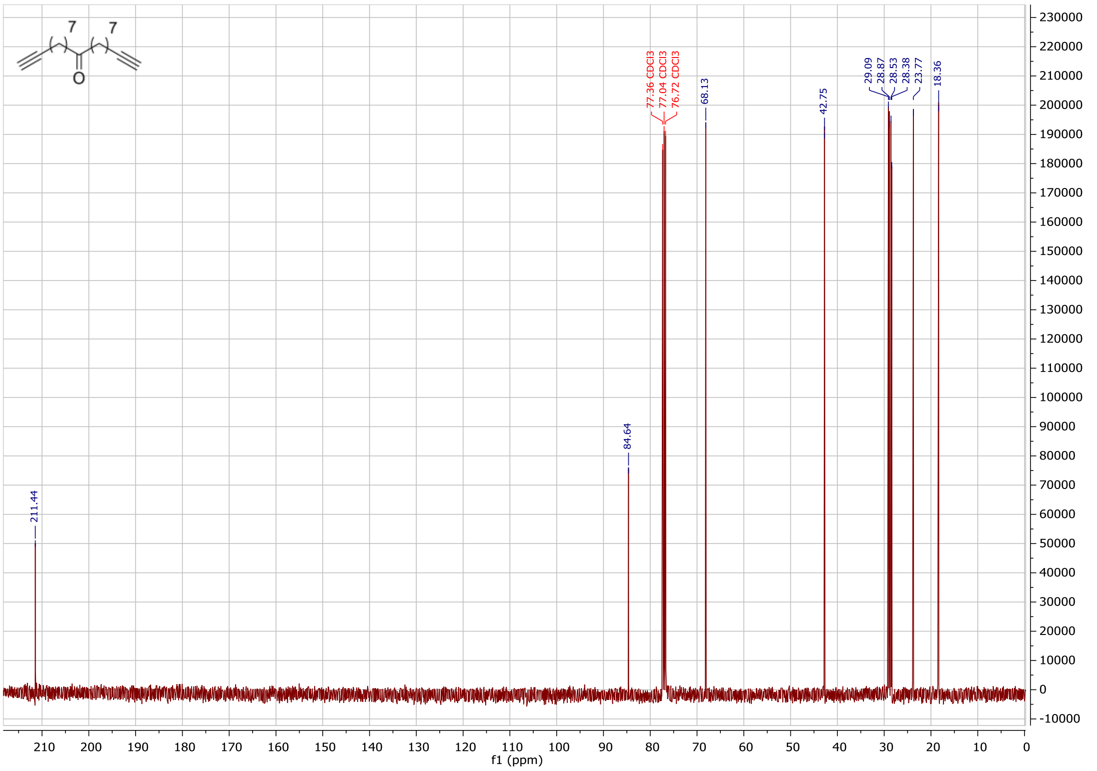

Figure S8. ^{13}C NMR, $CDCl_3$, compound **17**.

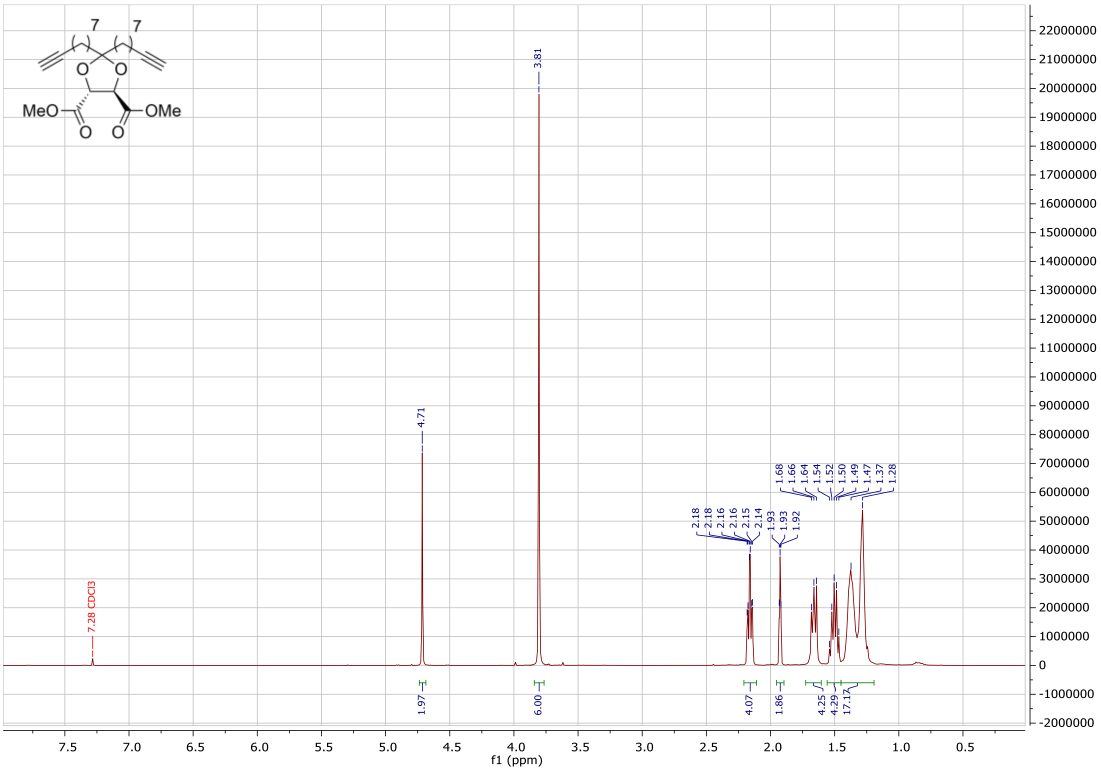

Figure S9. ^{1}H NMR, $CDCl_3$, compound **18**.

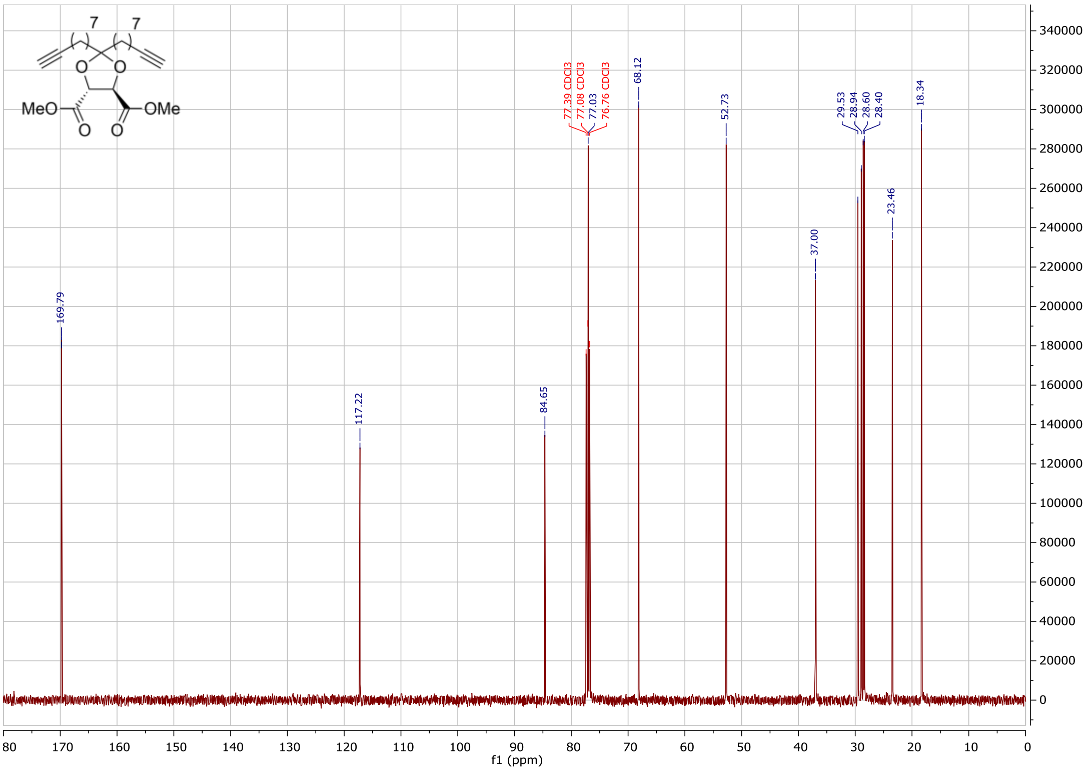

Figure S10. ^{13}C NMR, $CDCl_3$, compound **18**.

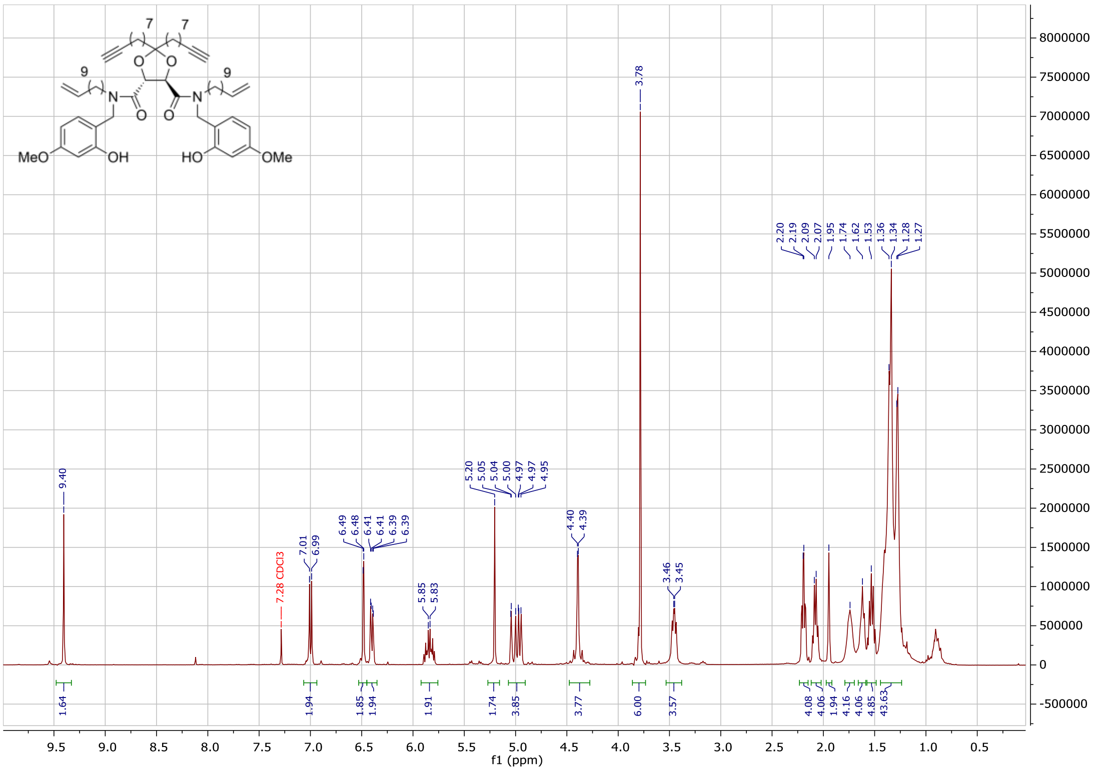

Figure S11. ^{1}H NMR, $CDCl_3$, compound **20**.

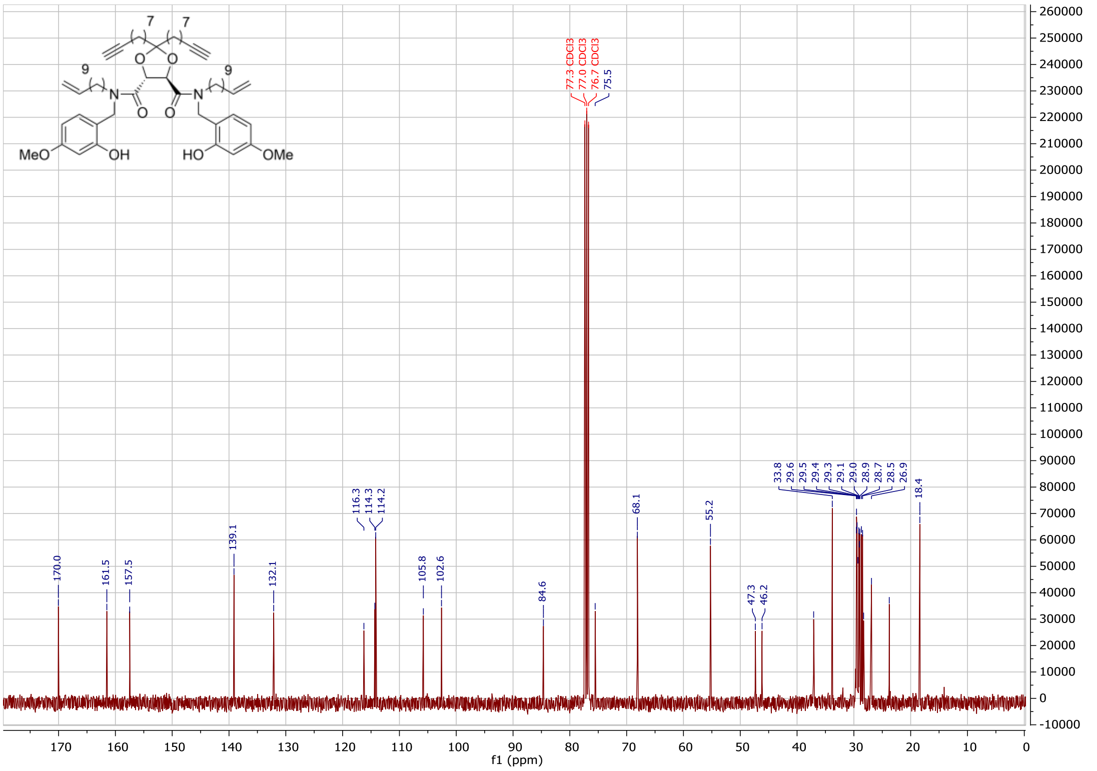

Figure S12. ^{13}C NMR, $CDCl_3$, compound **20**.

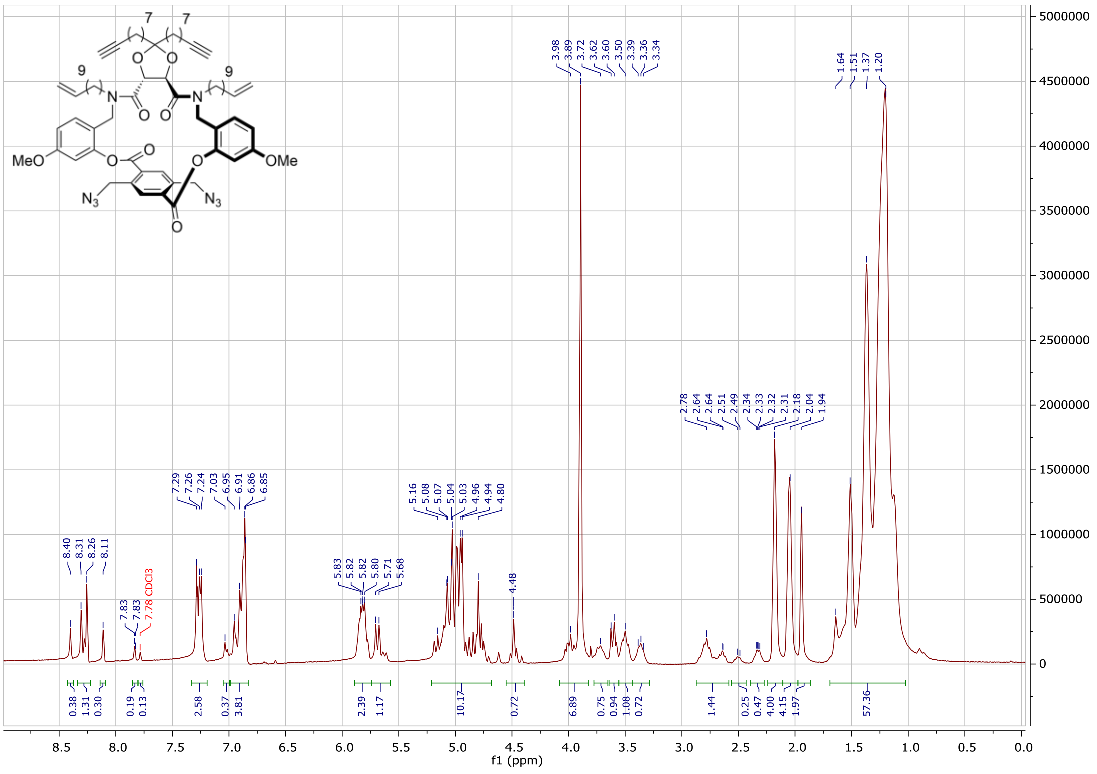

Figure S13. ^{1}H NMR, $CDCl_3$, compound **22b**.

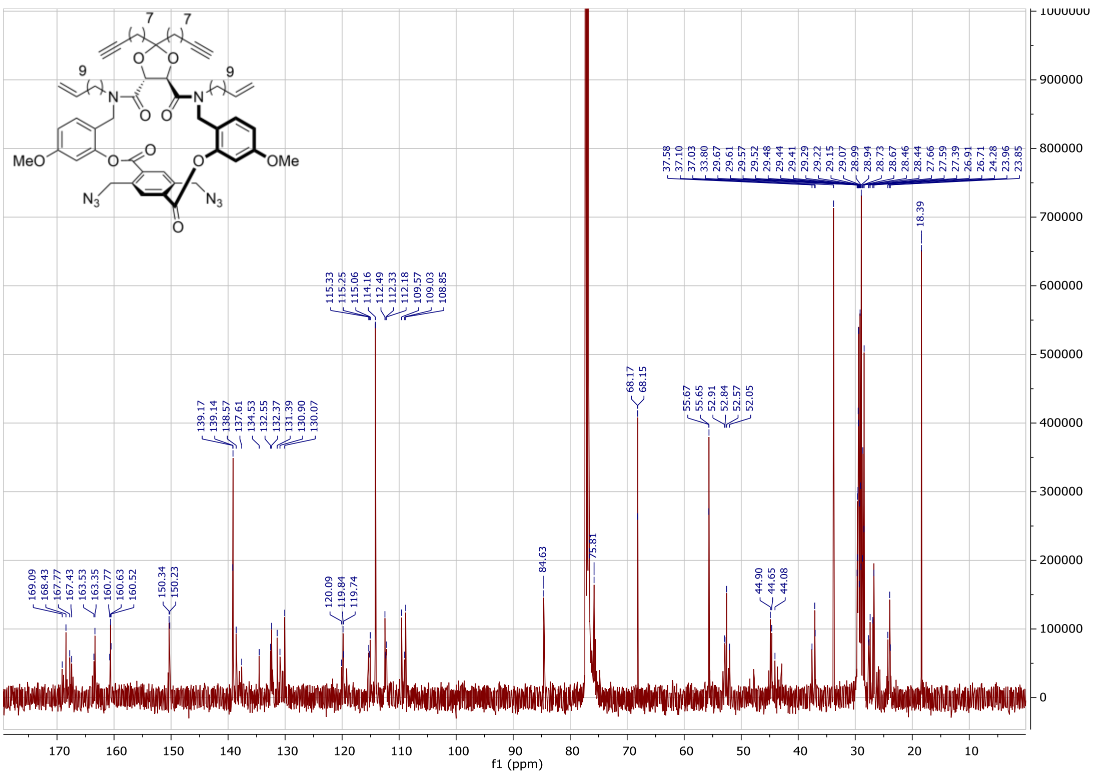

Figure S14. ^{13}C NMR, $CDCl_3$, compound **22b**.

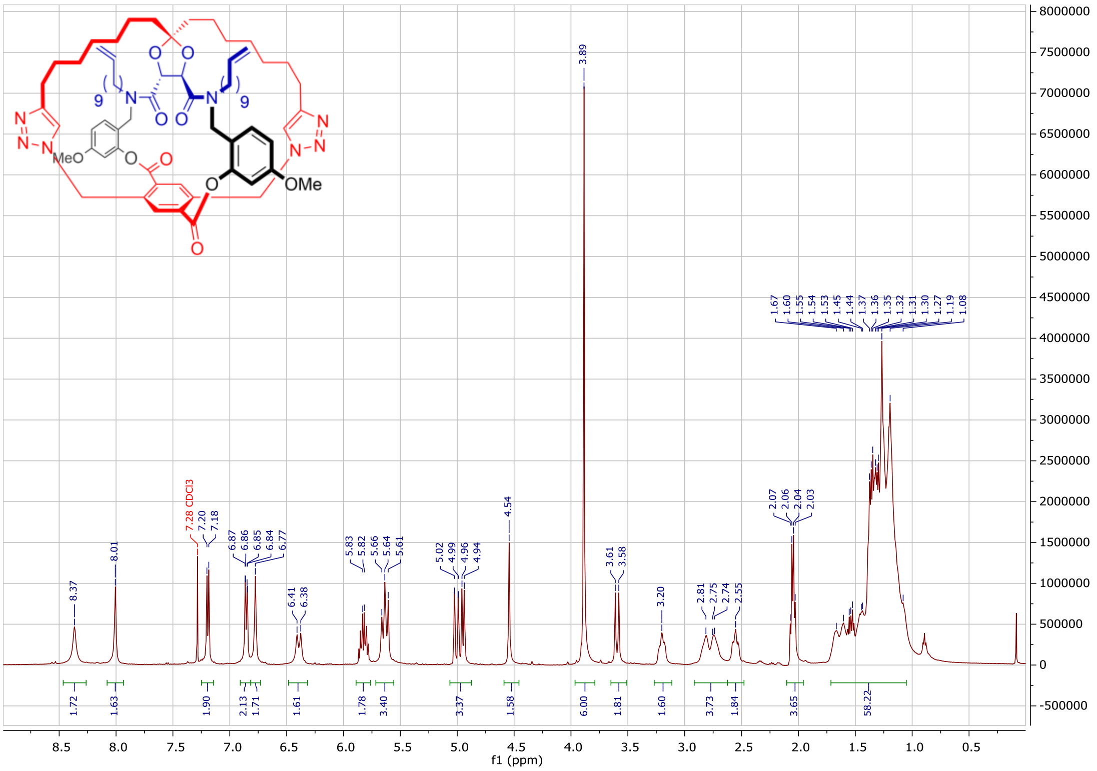

Figure S15. ^{1}H NMR, $CDCl_3$, compound **23b**.

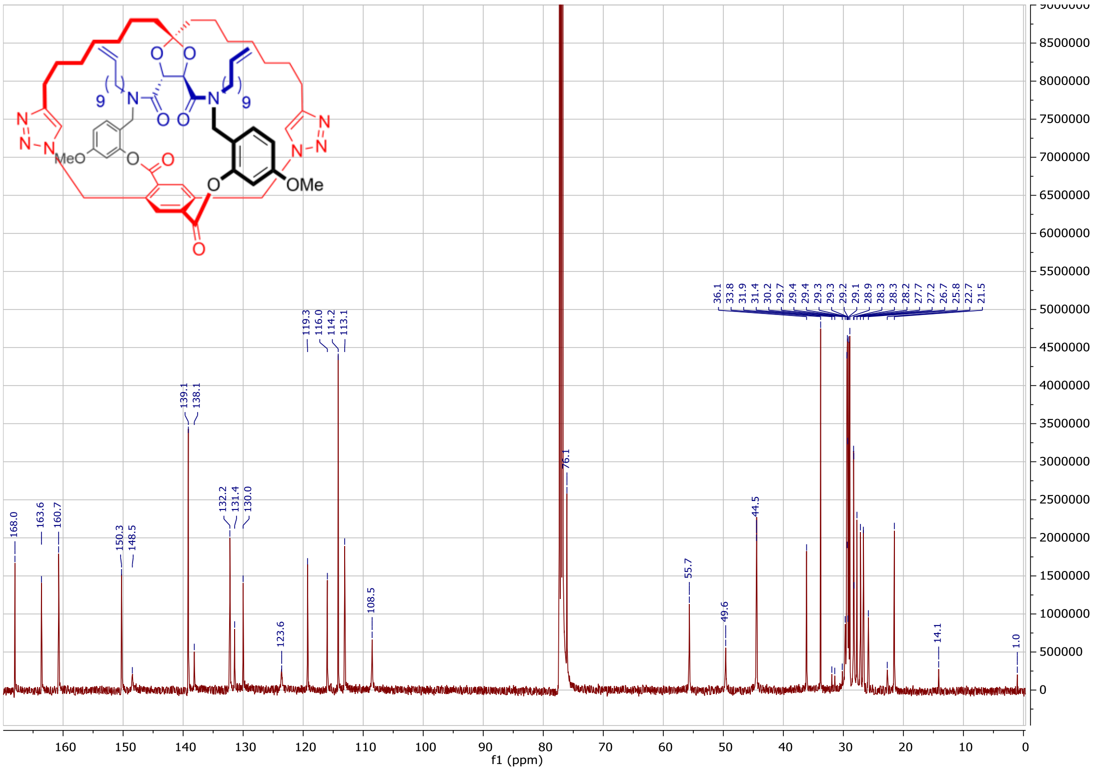

Figure S16. ^{13}C NMR, $CDCl_3$, compound **23b**.

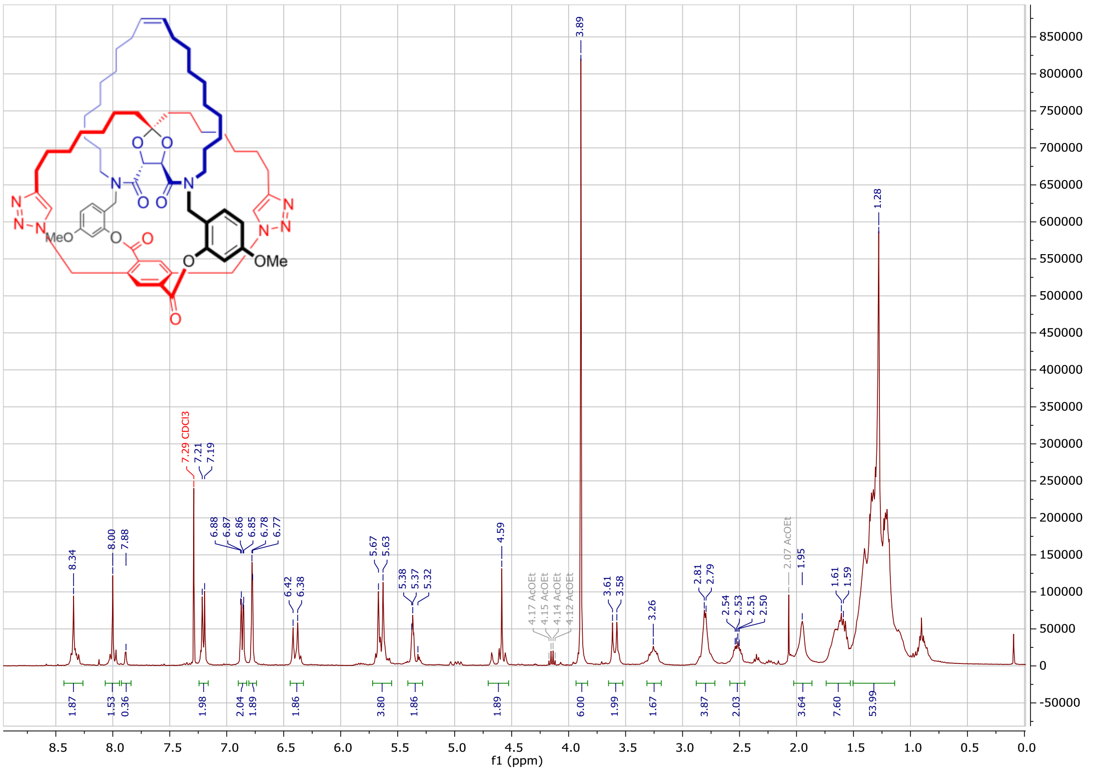

Figure S17. ^{1}H NMR, $CDCl_3$, compound **24b**.

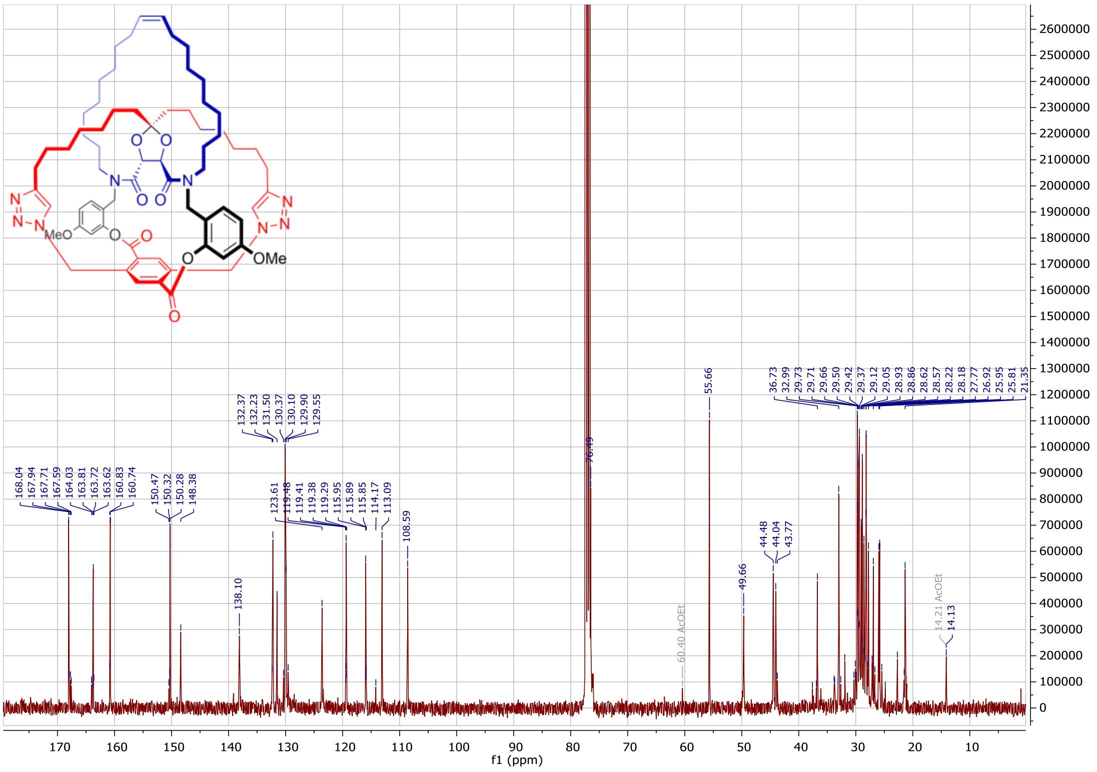

Figure S18. ^{13}C NMR, $CDCl_3$, compound **24b**.

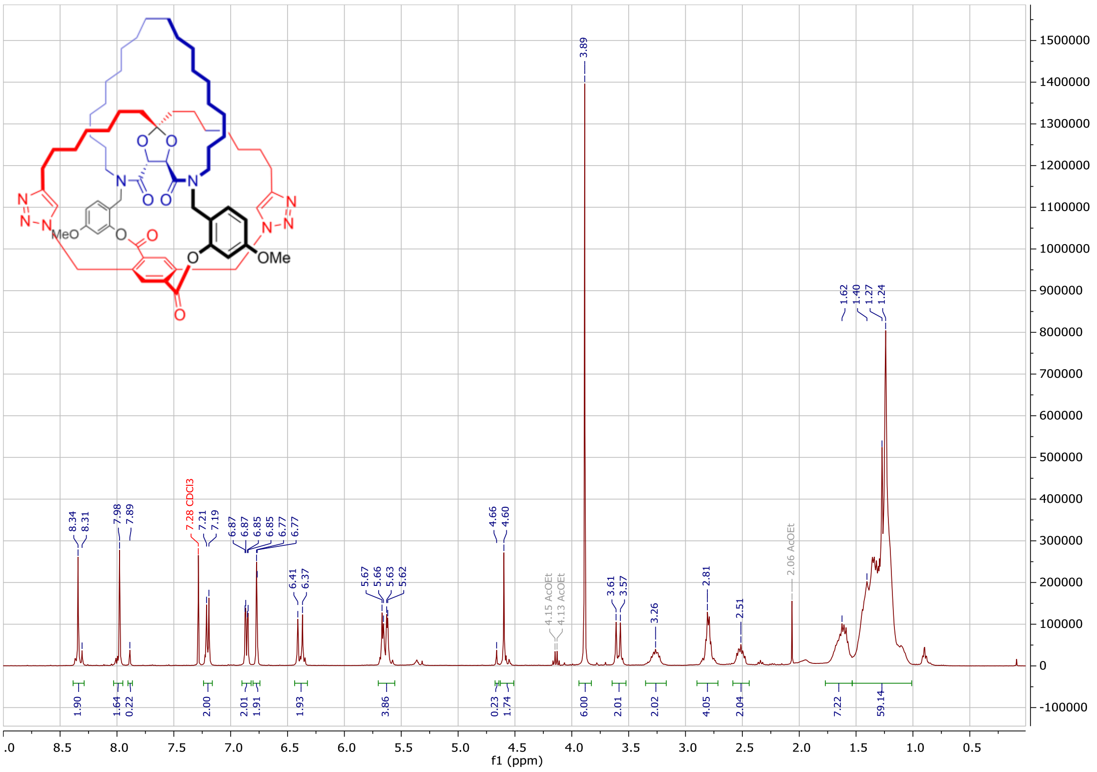

Figure S19. ^{1}H NMR, $CDCl_3$, compound **24b-H$_2$**.

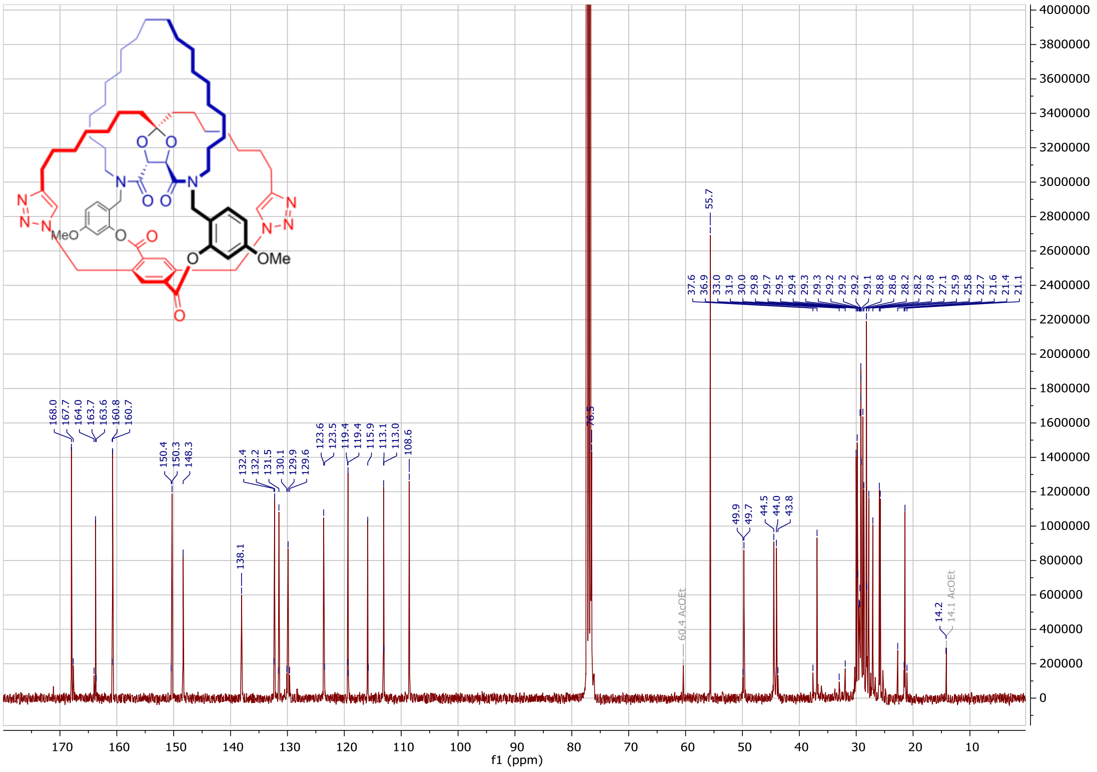

Figure S20. ^{13}C NMR, $CDCl_3$, compound **24b-H$_2$**.

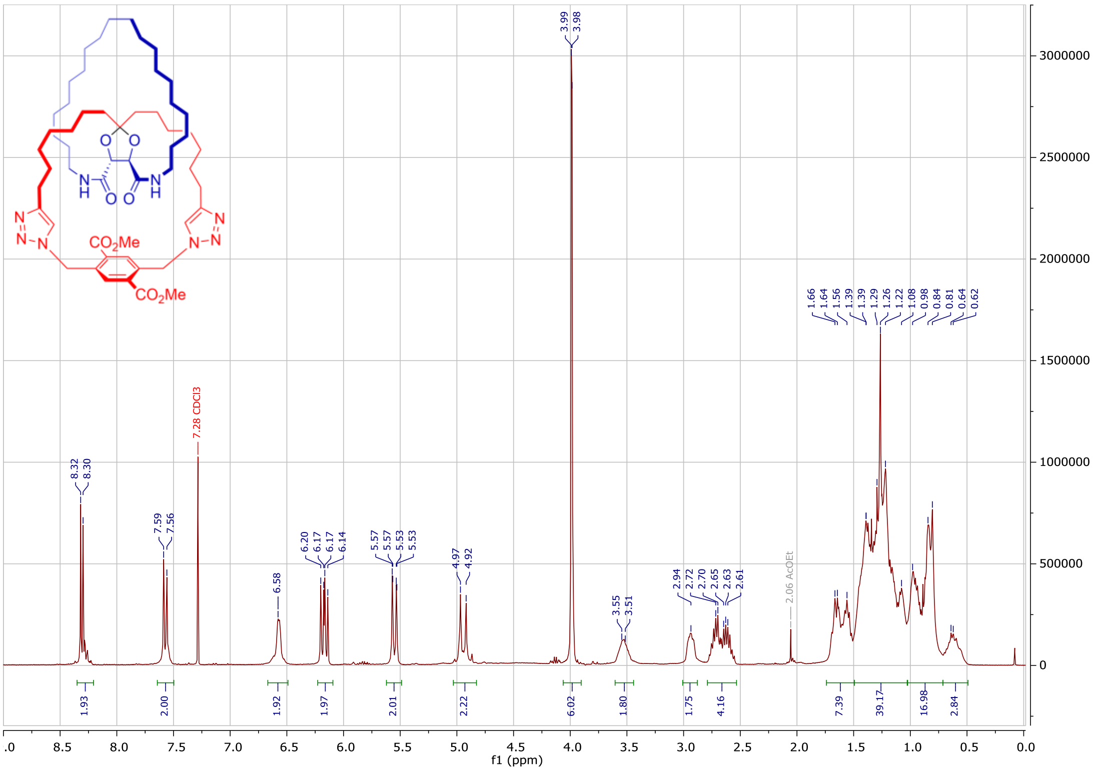

Figure S21. ^{1}H NMR, $CDCl_3$, compound **9b**.

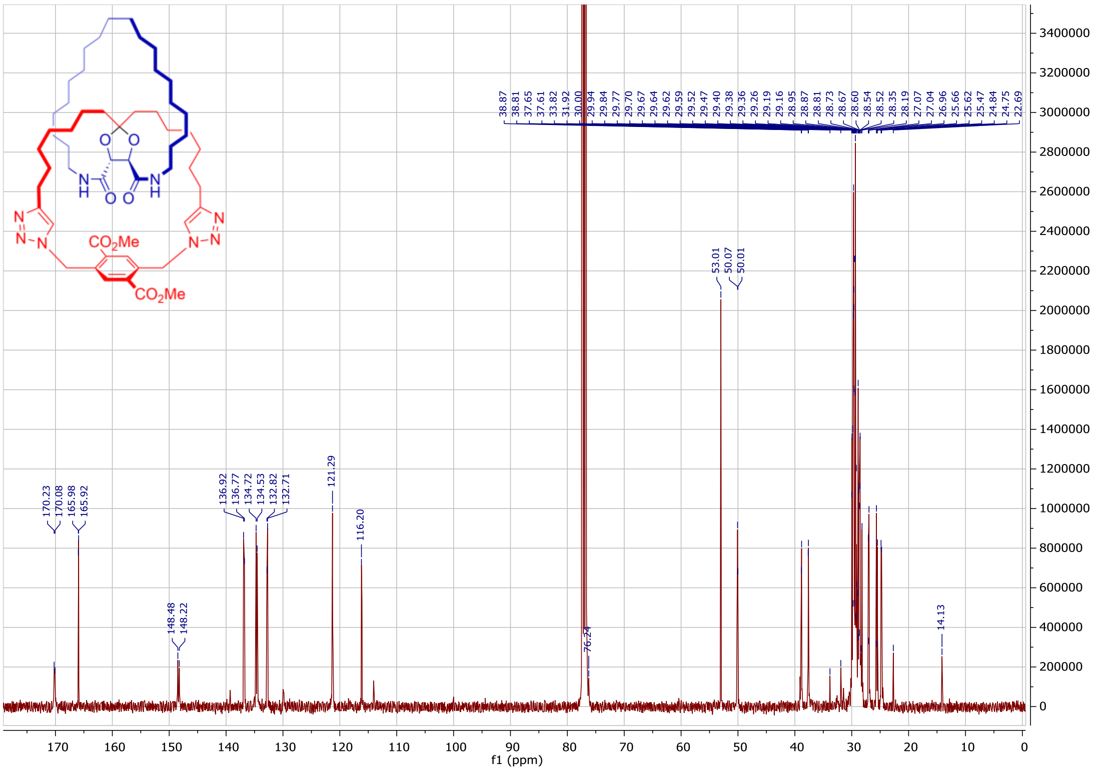

Figure S22. ^{13}C NMR, $CDCl_3$, compound **9b**.

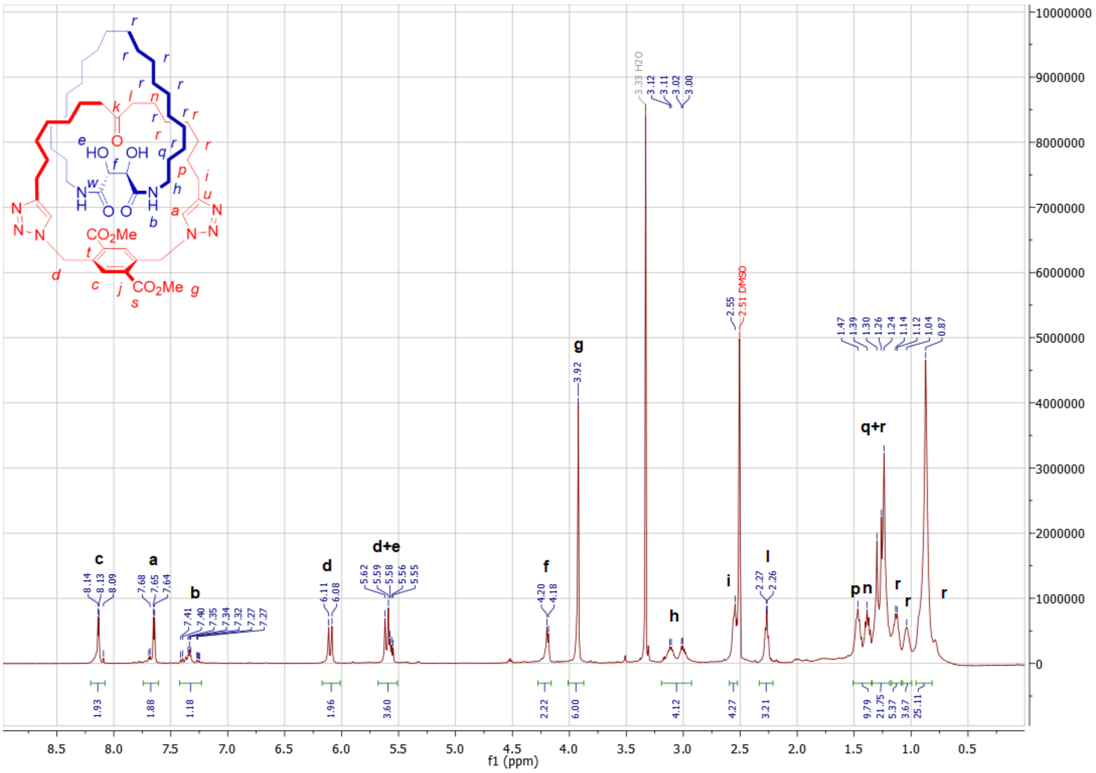

Figure S23. ^{1}H NMR, $(CD_3)_2SO$, compound **10b**.

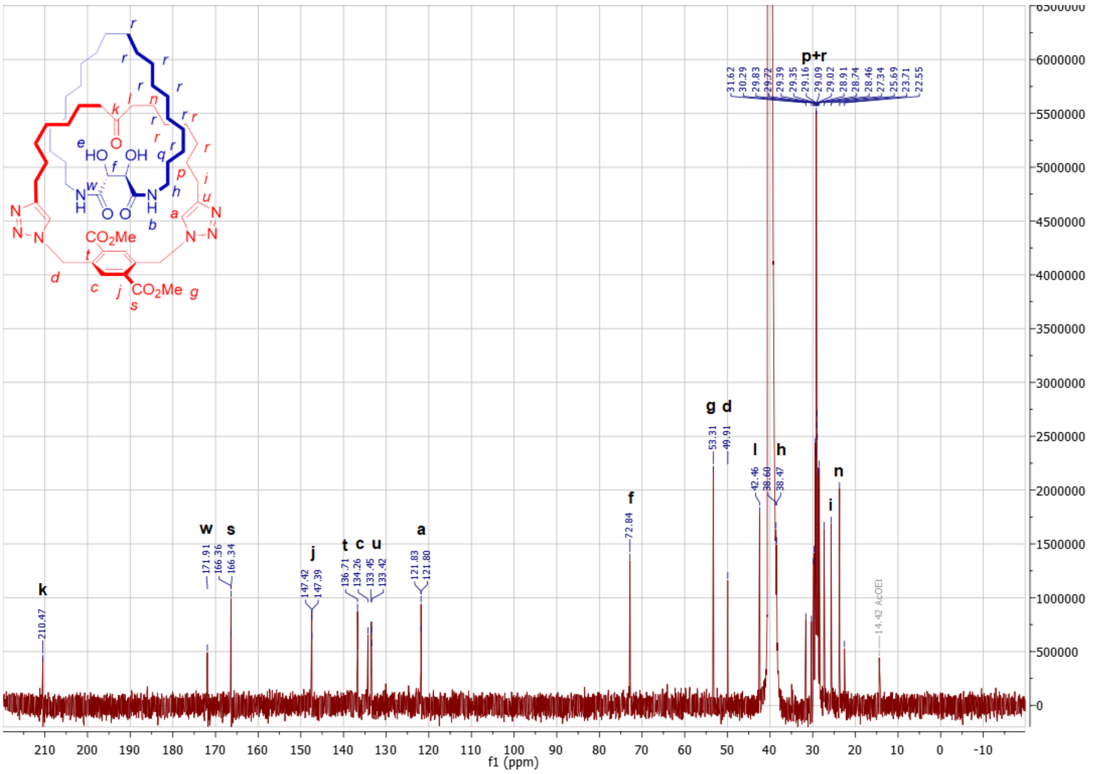

Figure S24. ^{13}C NMR, $(CD_3)_2SO$, compound **10b**.

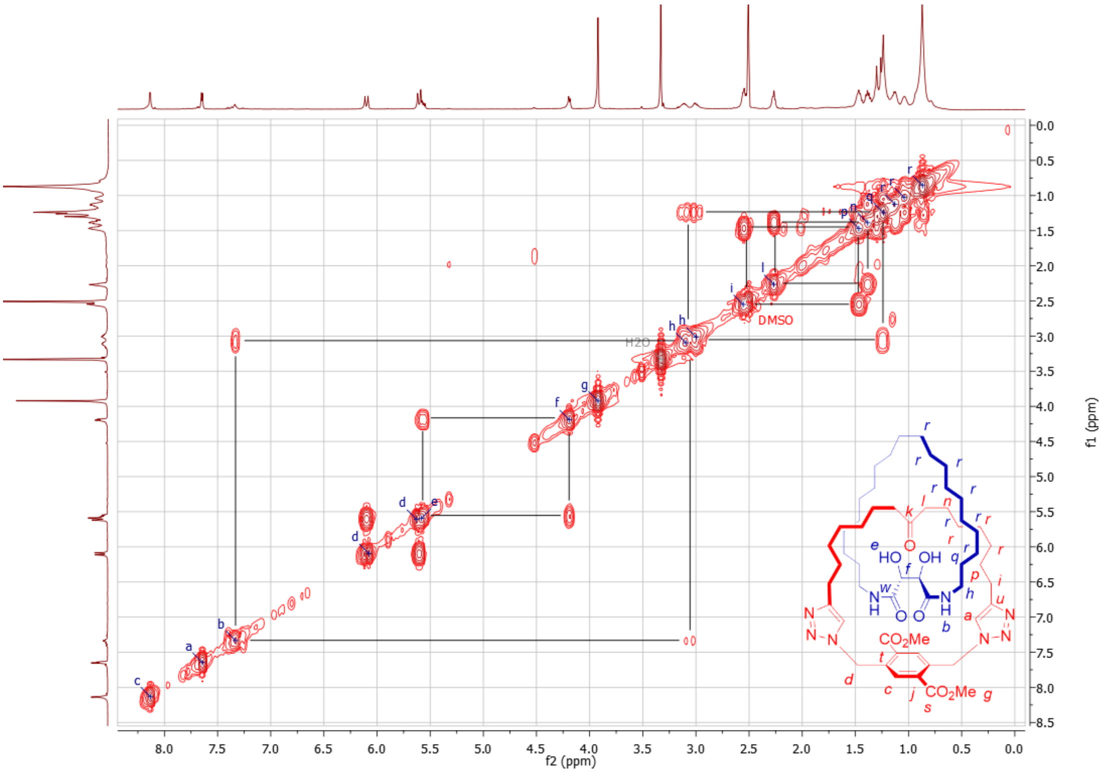

Figure S25. COSY NMR, $(CD_3)_2SO$, compound **10b**.

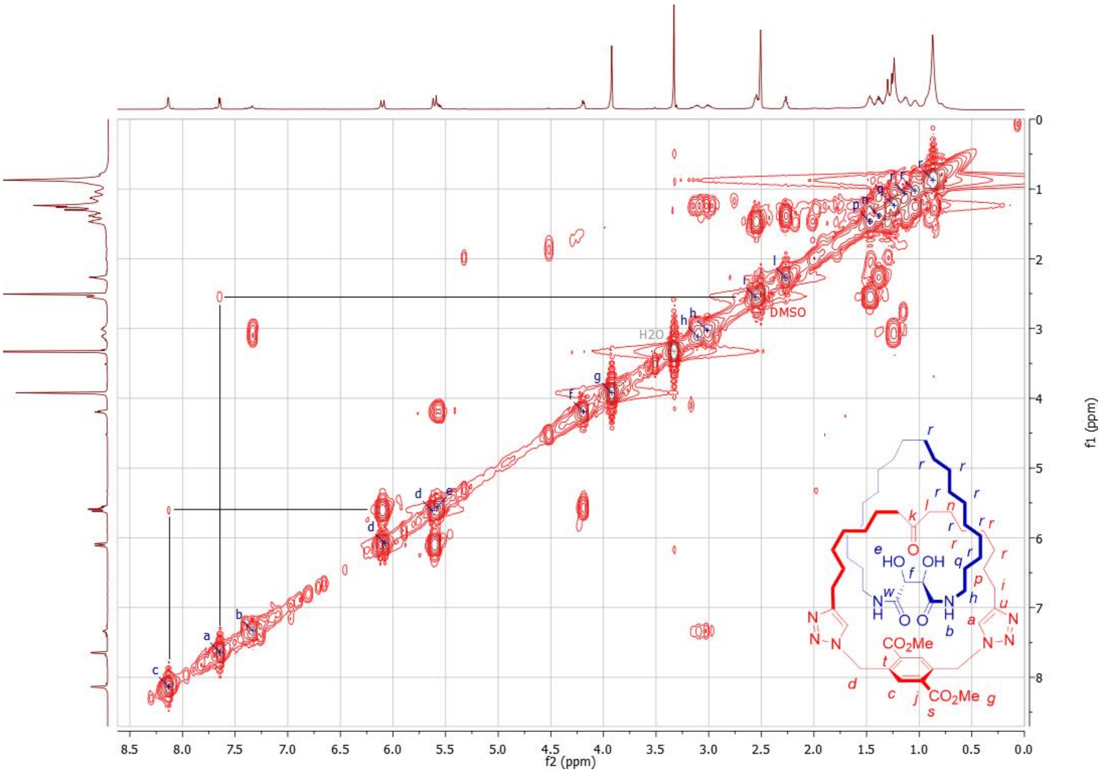

Figure S26. COSY NMR, $(CD_3)_2SO$, compound **10b**, with long range $^3J_{HH}$ couplings of the aromatic protons *a* and *c*.

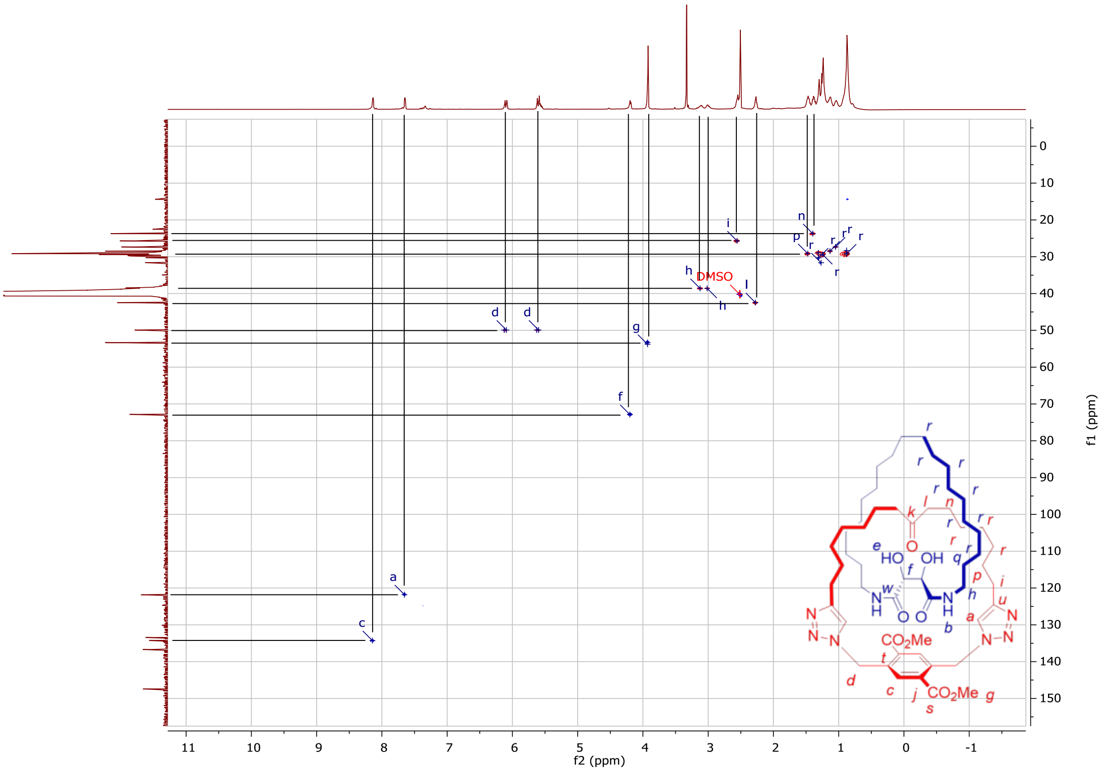

Figure S27. HSQC NMR, $(CD_3)_2SO$, compound **10b**.

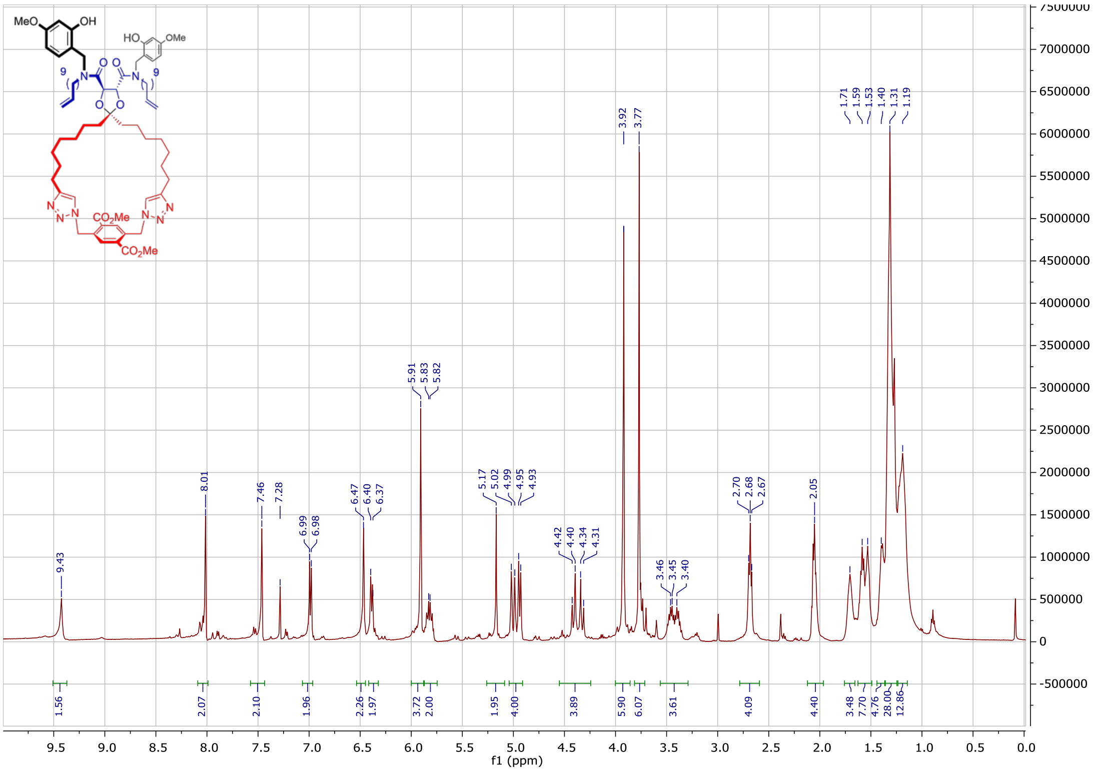

Figure S28. ^{1}H NMR, $CDCl_3$, **25b**.

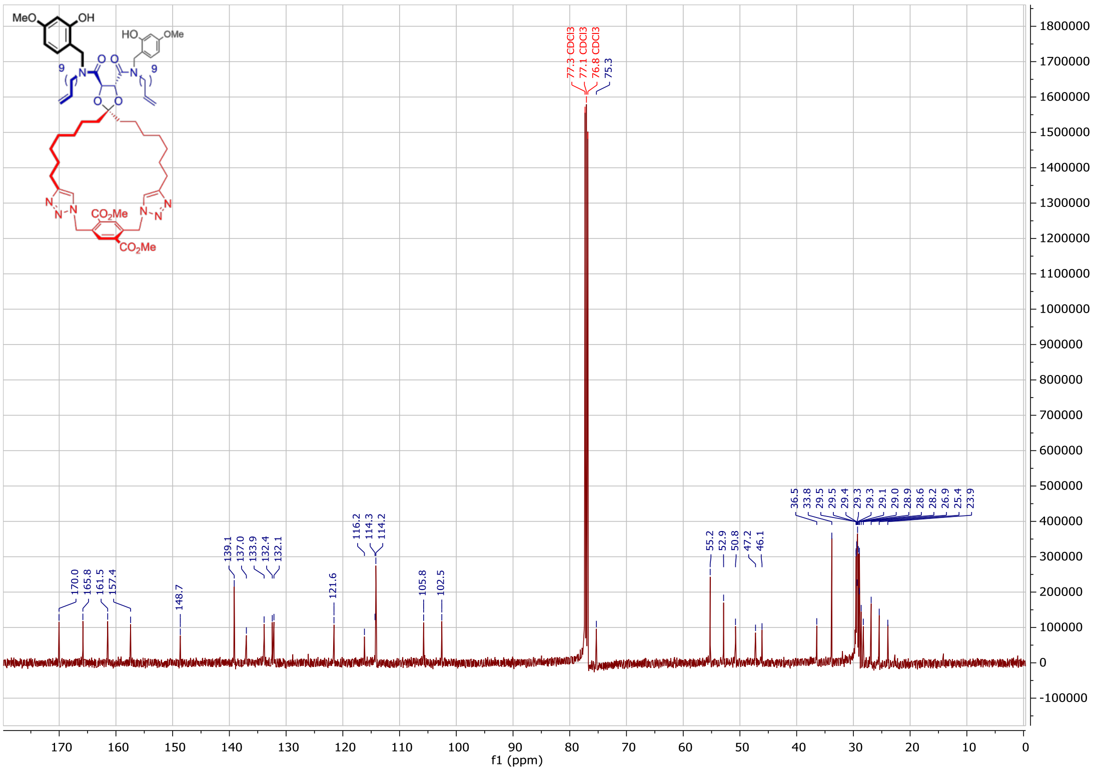

Figure S29. ^{13}C NMR, $CDCl_3$, **25b**.

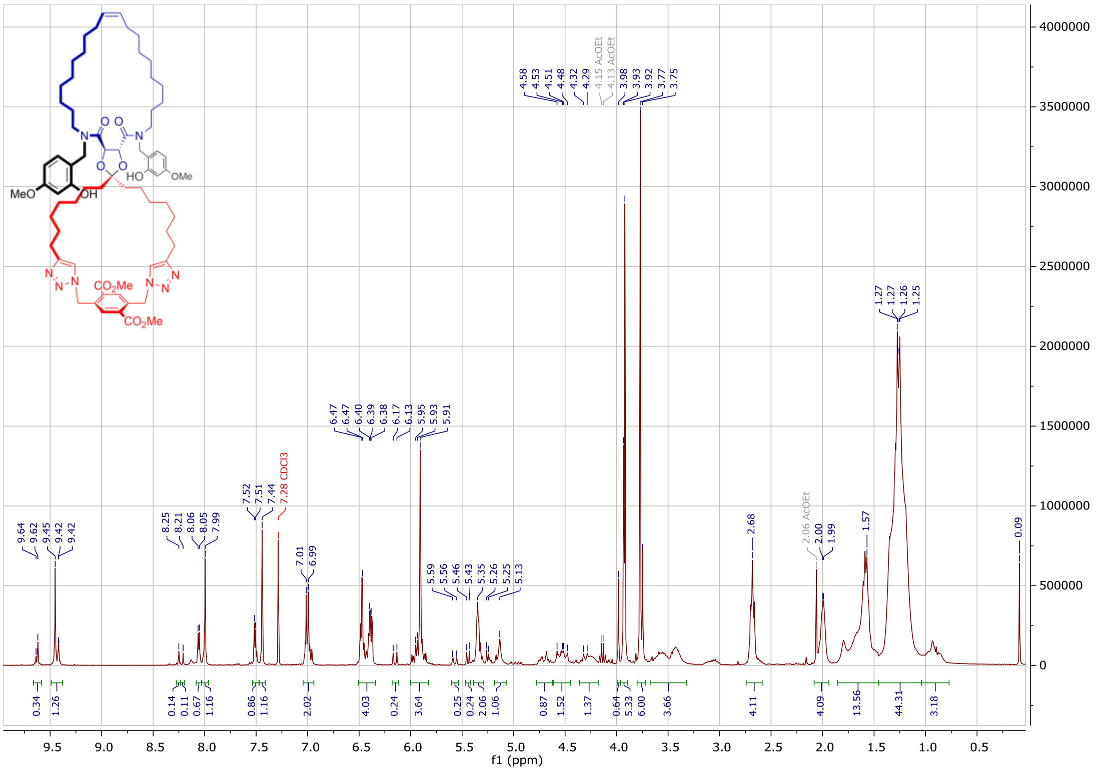

Figure S30. ^{1}H NMR, $CDCl_3$, **26b**.

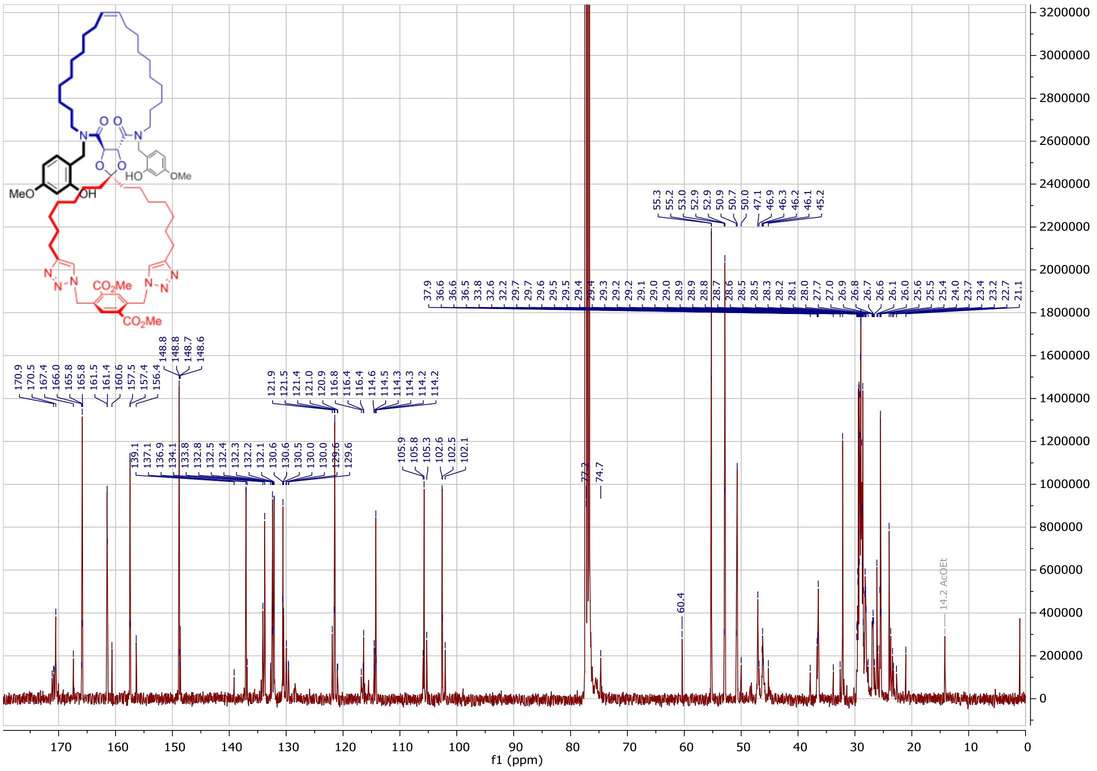

Figure S31. ^{13}C NMR, $CDCl_3$, **26b**.

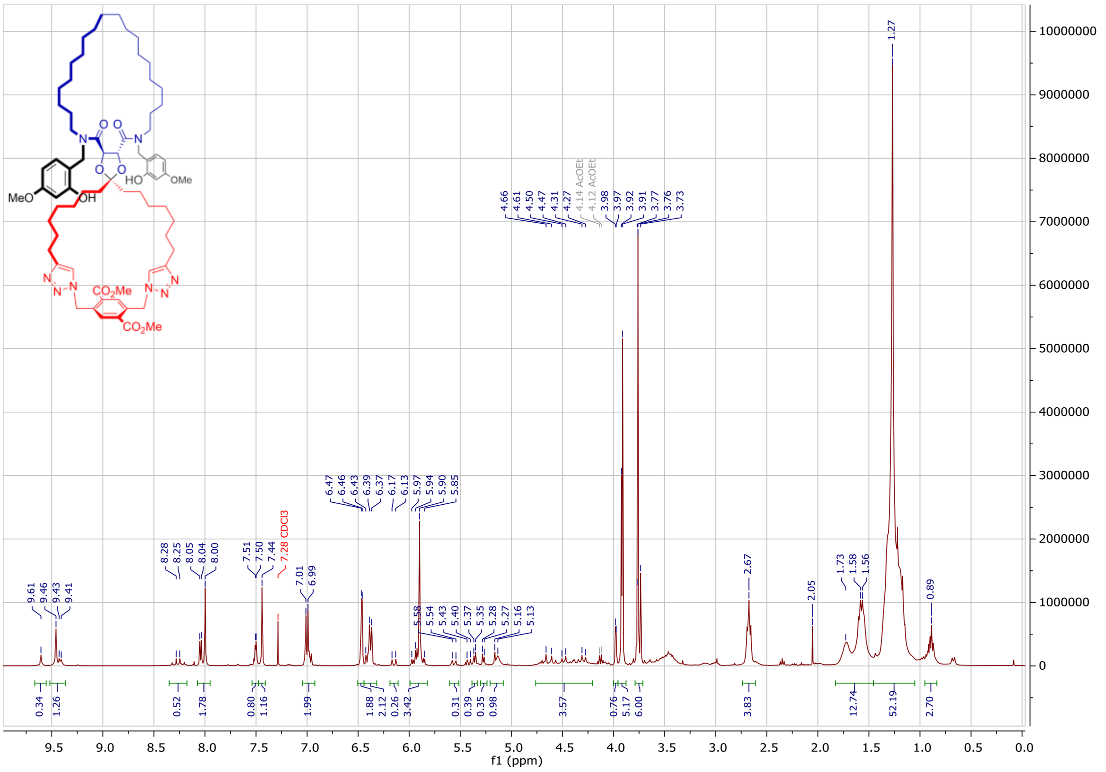

Figure S32. ^{1}H NMR, $CDCl_3$, **26b-H₂**.

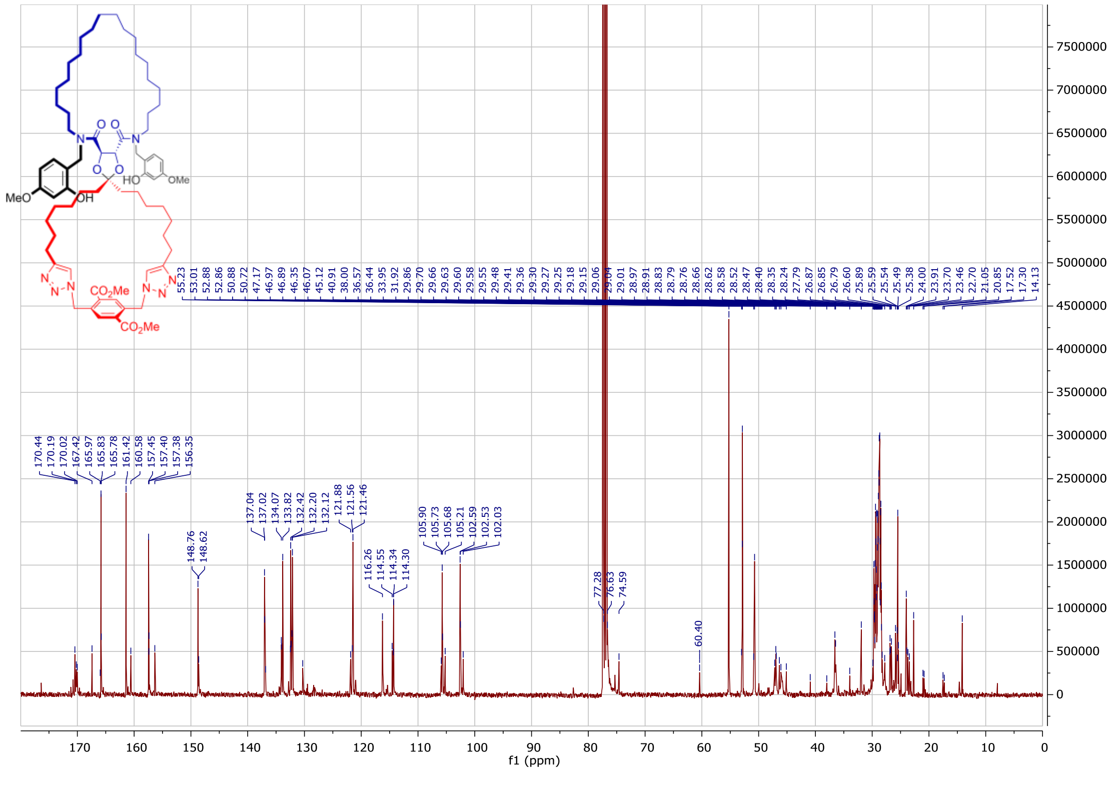

Figure S33. ^{13}C NMR, $CDCl_3$, **26b-H₂**.

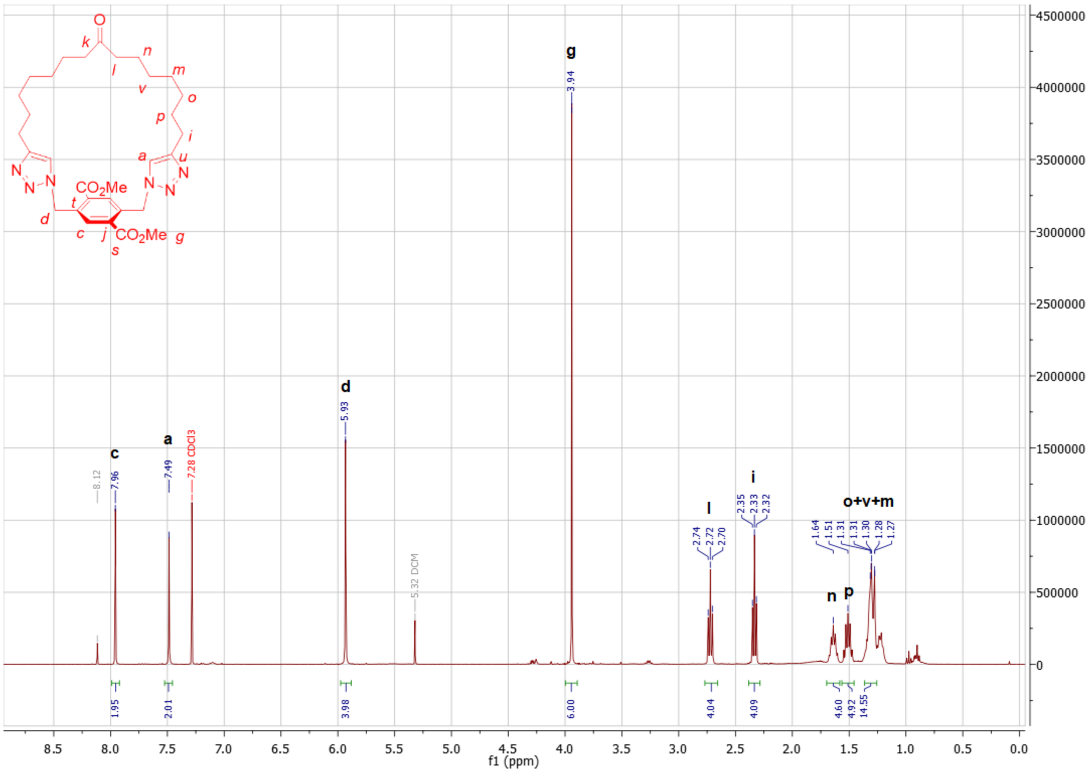

Figure S34. ^{1}H NMR, $CDCl_3$, **28b**.

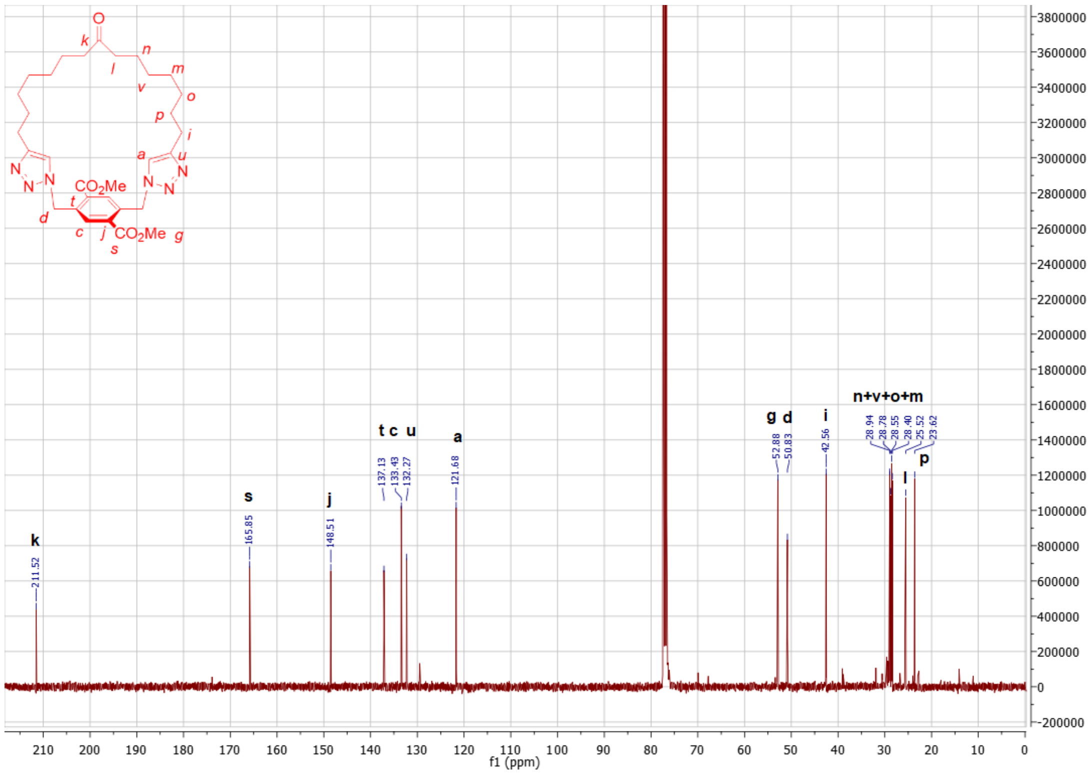

Figure S35. ^{13}C NMR, $CDCl_3$, **28b**.

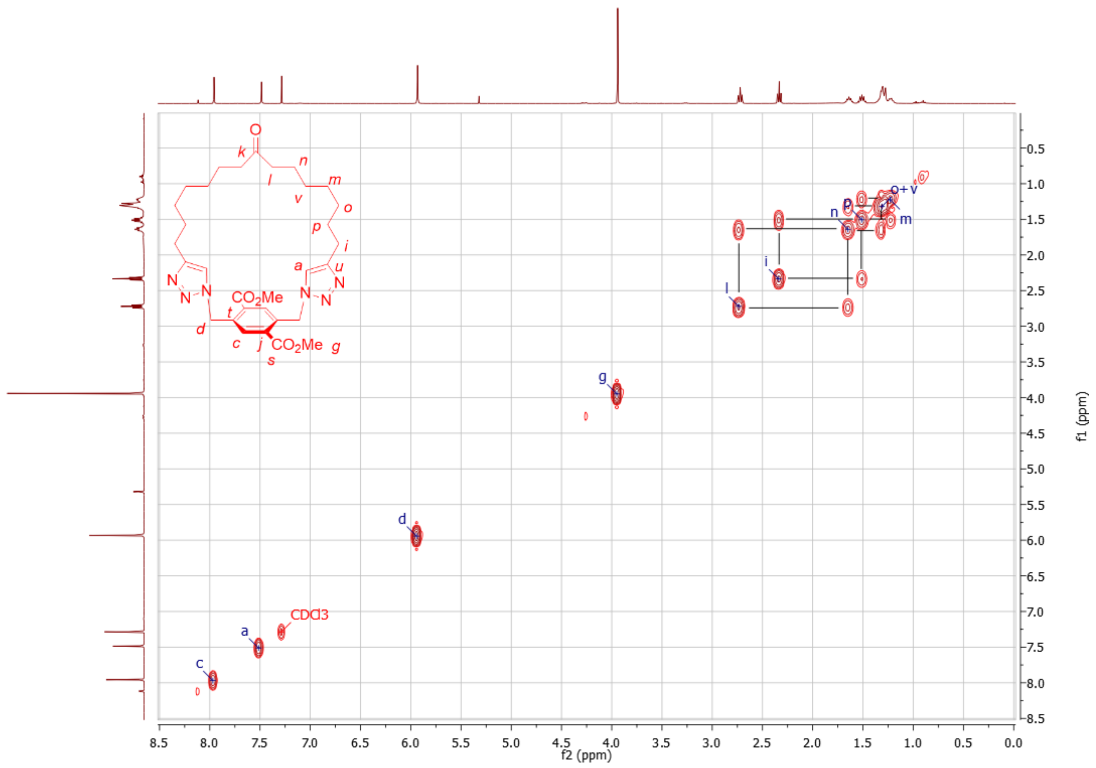

Figure S36. COSY NMR, $CDCl_3$, **28b**.

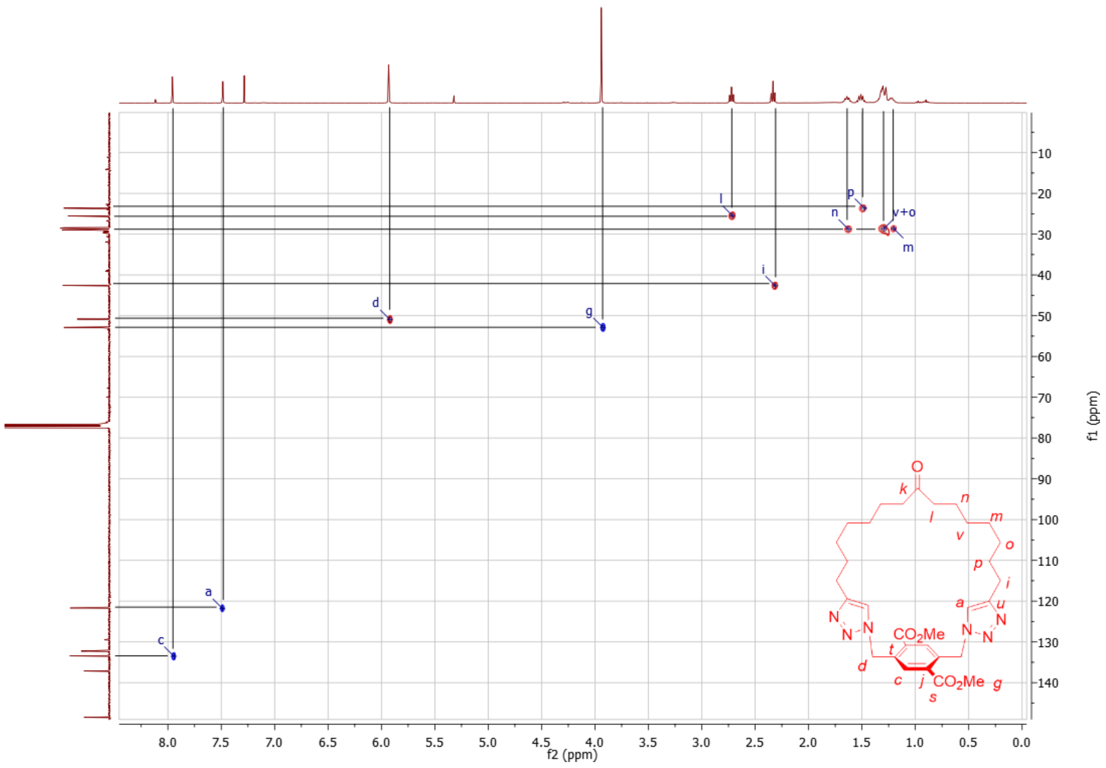

Figure S37. HSQC NMR, $CDCl_3$, **28b**.

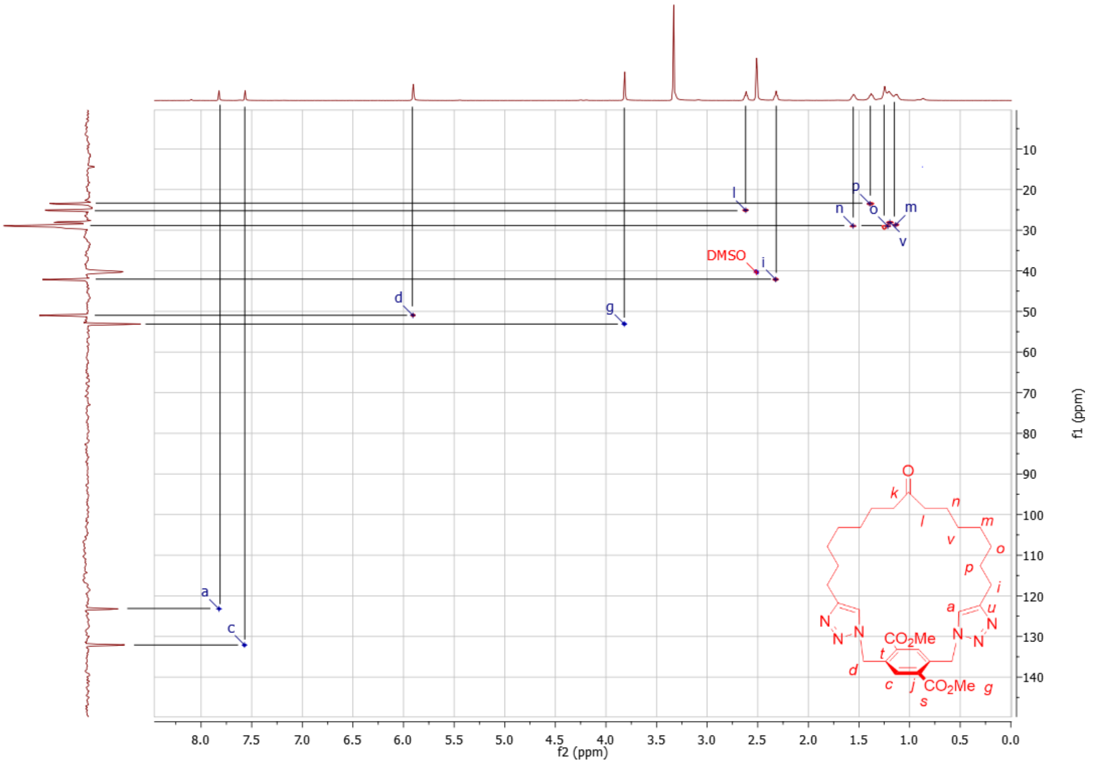

Figure S38. HSQC NMR, $(CD_3)_2SO$, **28b**. Note how proton peaks *c* and *a* swap positions in $(CD_3)_2SO$ relative to the spectra taken in $CDCl_3$, this is relevant for the assignment of peaks *a* and *c* in the equimolar mixture spectrum in Figure S43 .

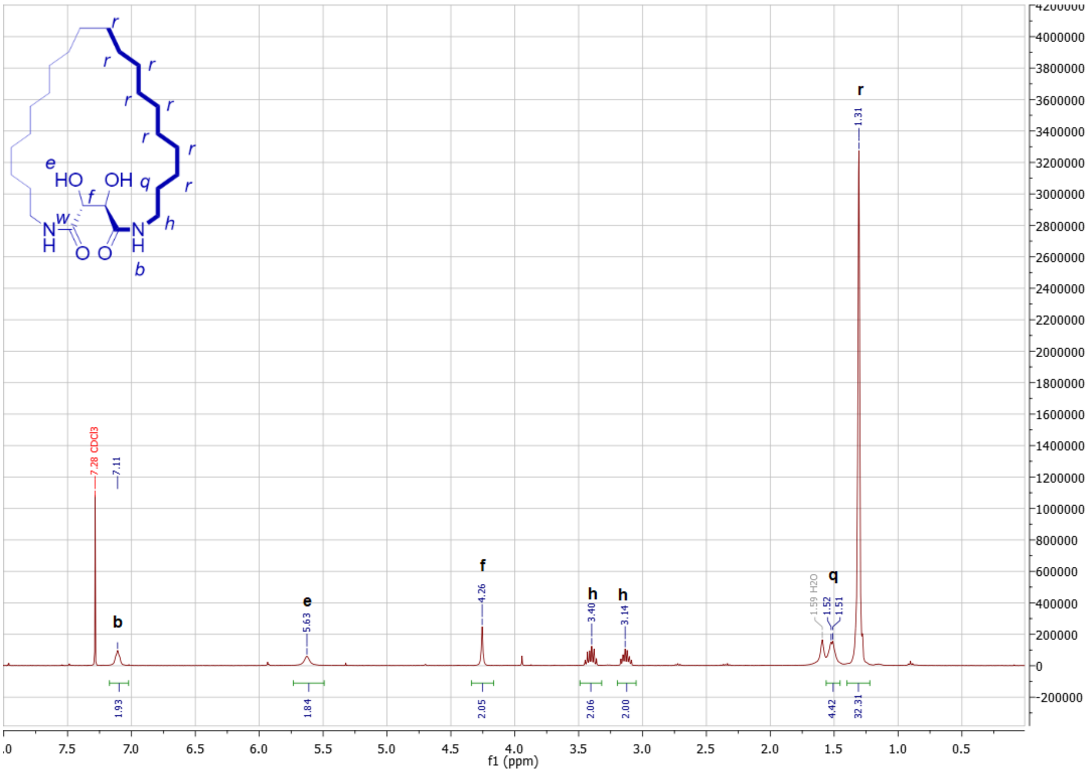

Figure S39. ^{1}H NMR, $CDCl_3$, **29**.

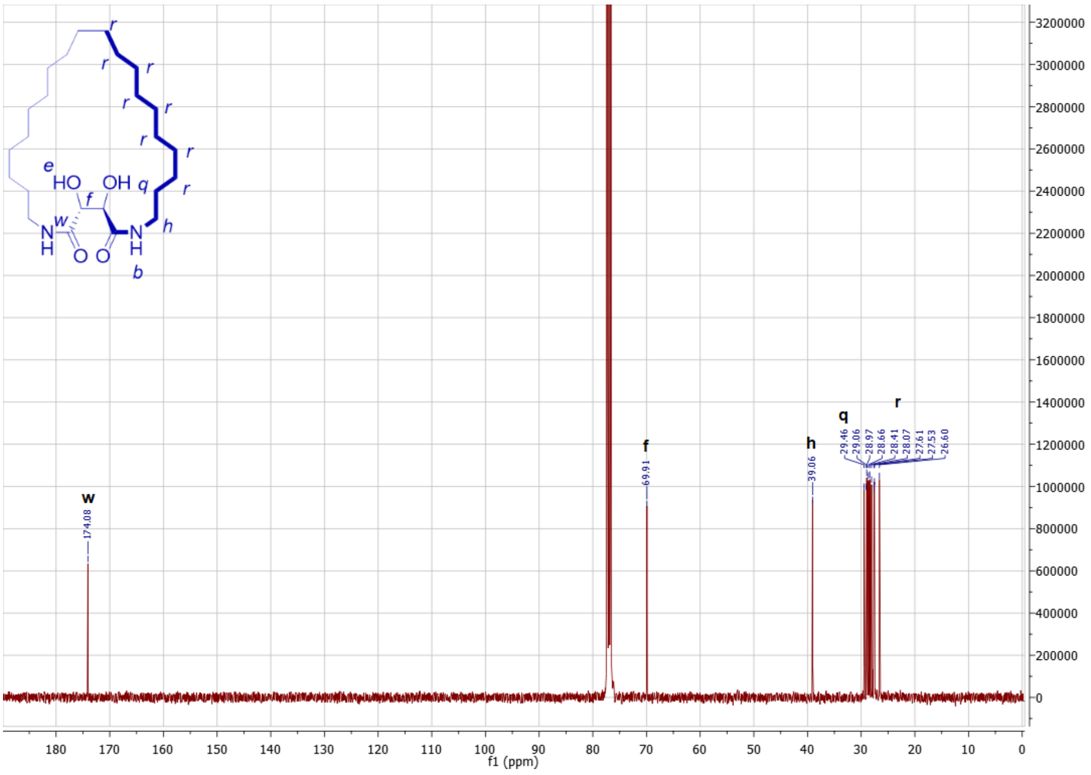

Figure S40. ^{13}C NMR, $CDCl_3$, **29**.

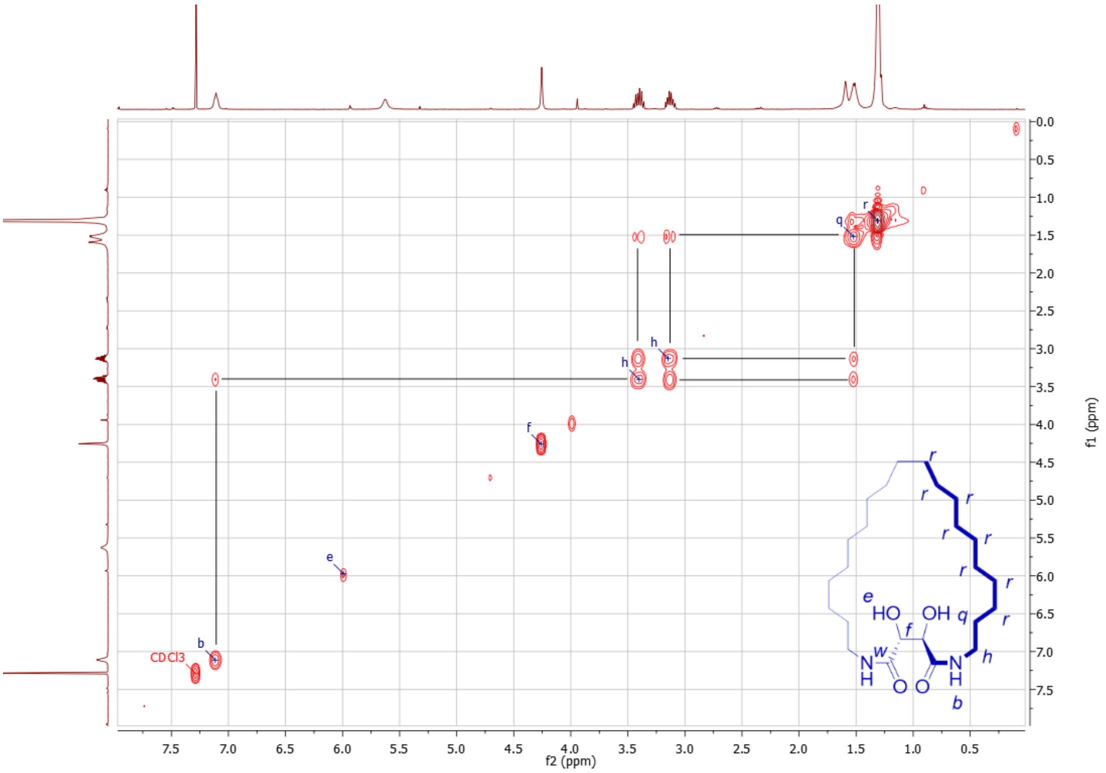

Figure S41. COSY NMR, $CDCl_3$, **29**.

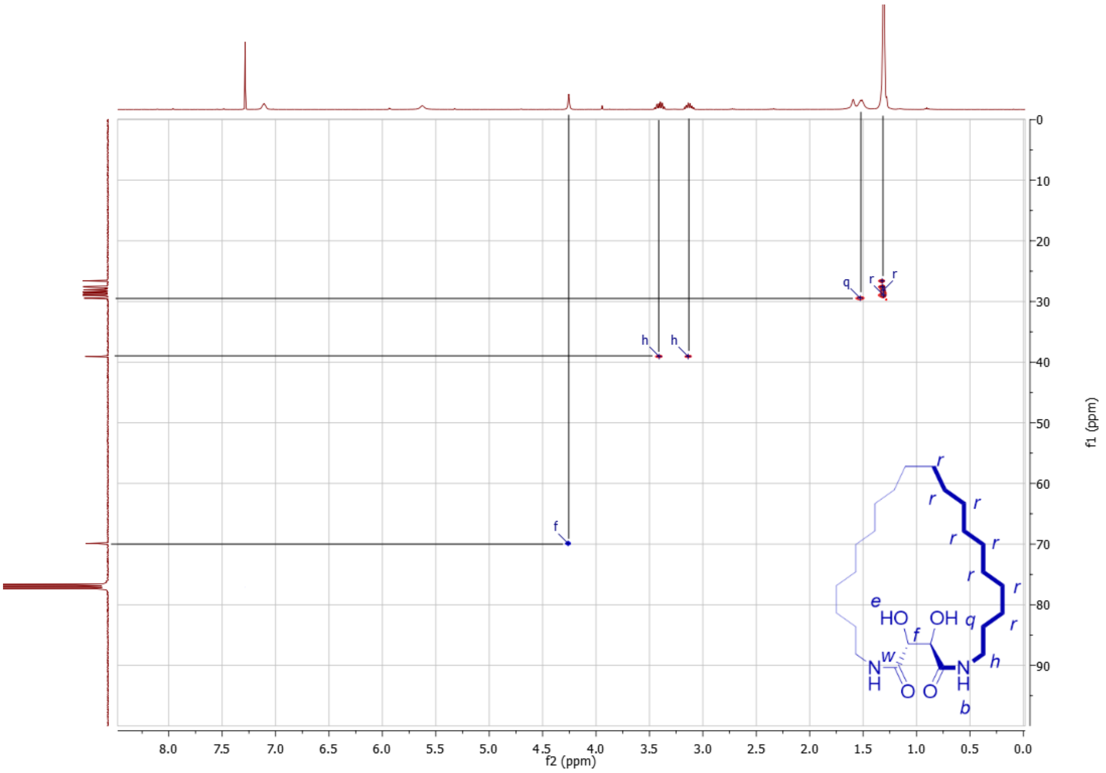

Figure S42. HSQC NMR, $CDCl_3$, **29**.

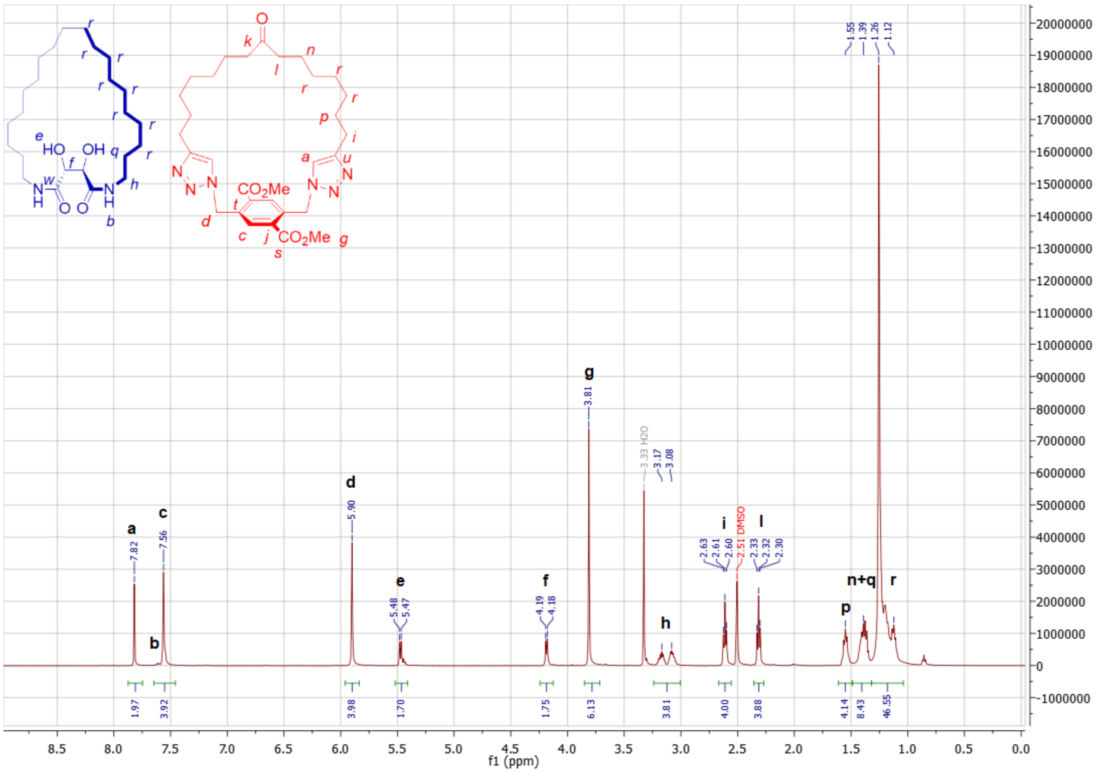

Figure S43. ^{1}H NMR, $(CD_3)_2SO$, equimolar mixture of **28b** and **29**.

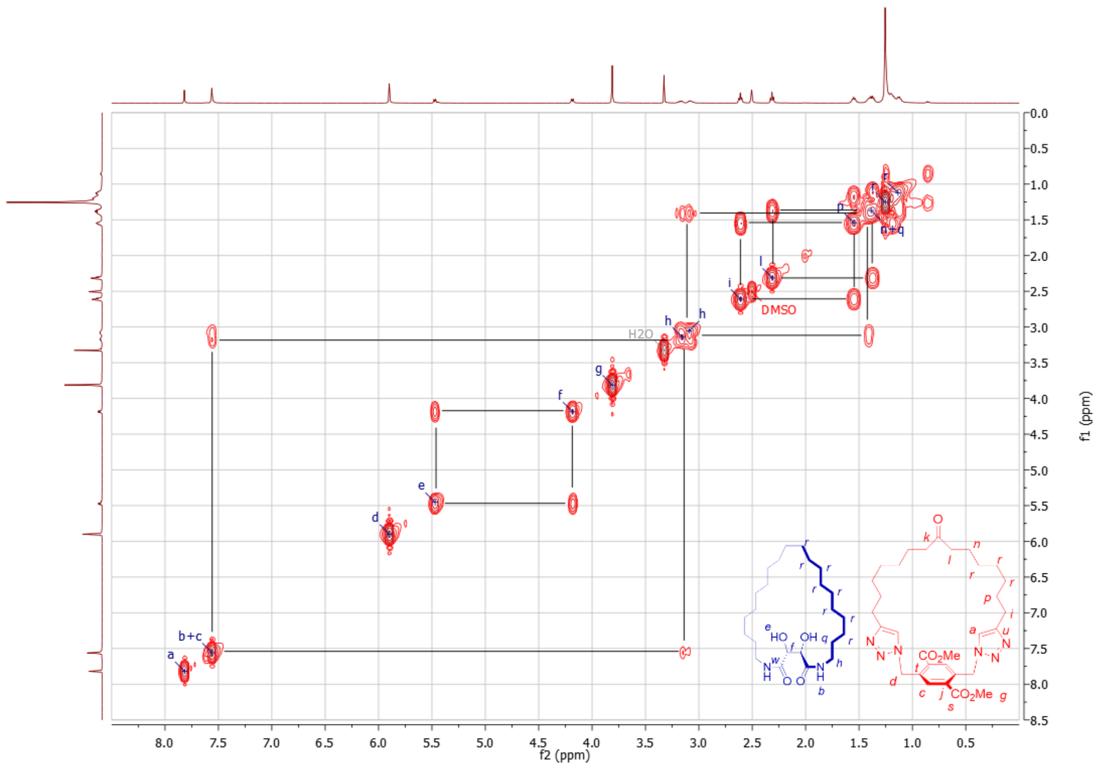

Figure S44. COSY NMR, $(CD_3)_2SO$, equimolar mixture of **28b** and **29**.

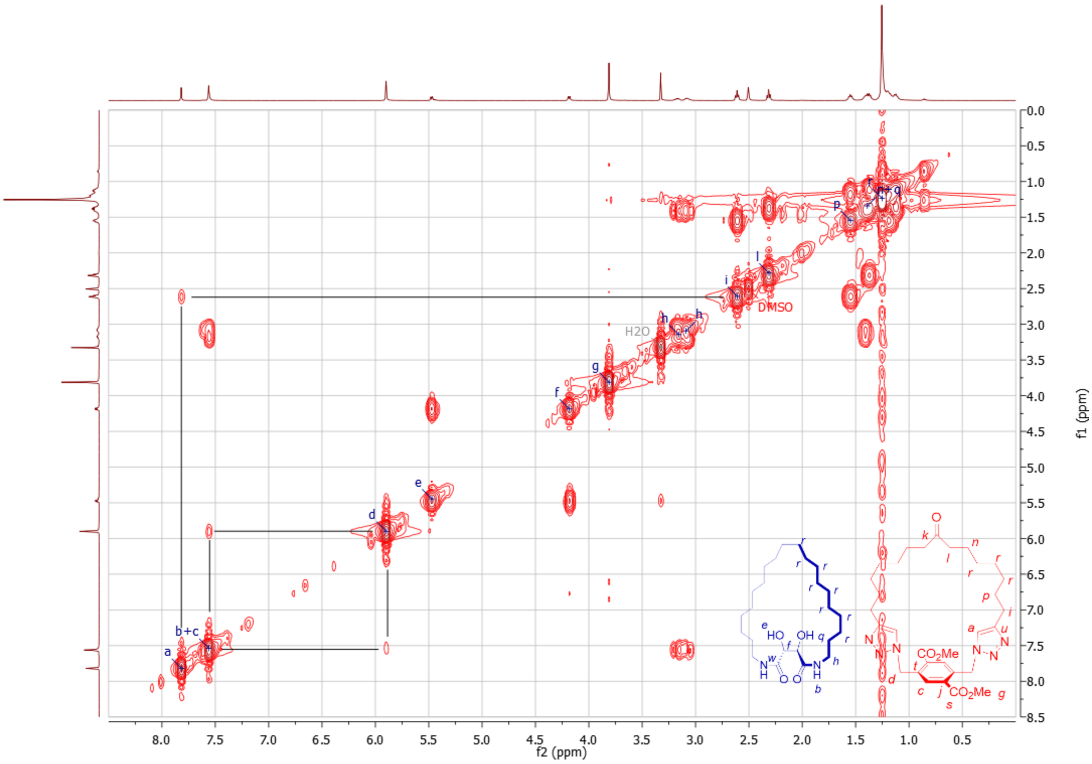

Figure S45. COSY NMR, $(CD_3)_2SO$, equimolar mixture of **28b** and **29**, with long range $^3J_{HH}$ couplings of the aromatic protons *a* and *c*.

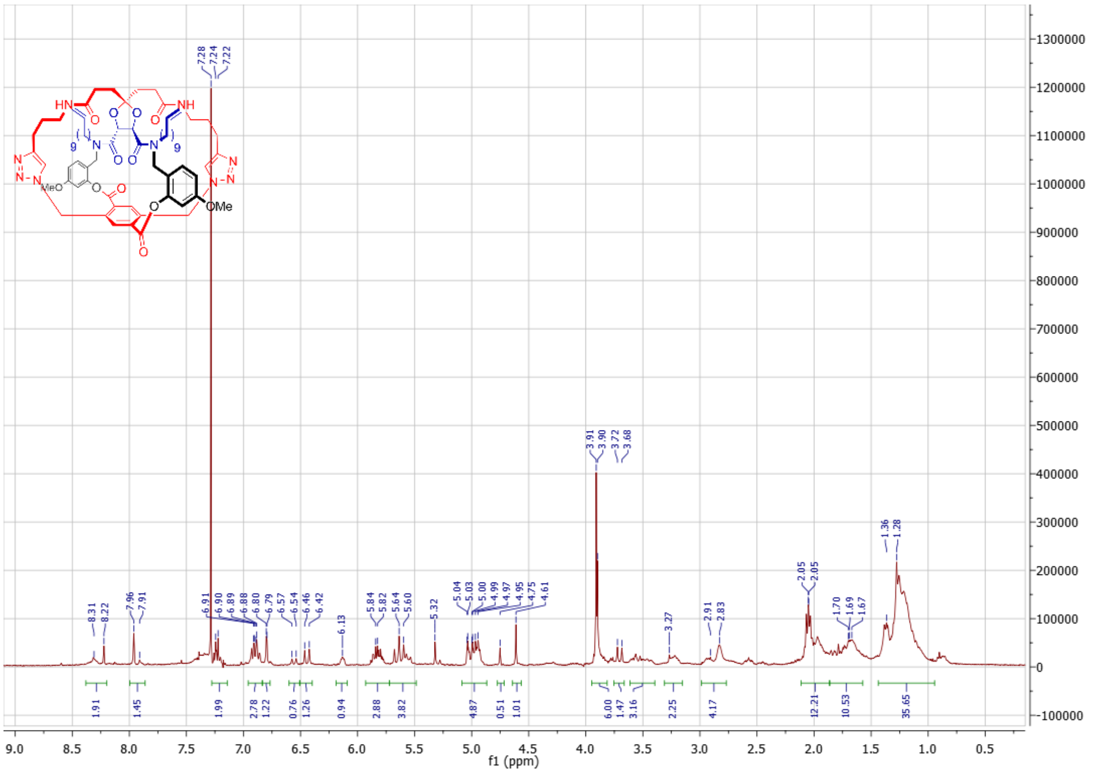

Figure S46. ^{1}H NMR, $CDCl_3$, **23a**.

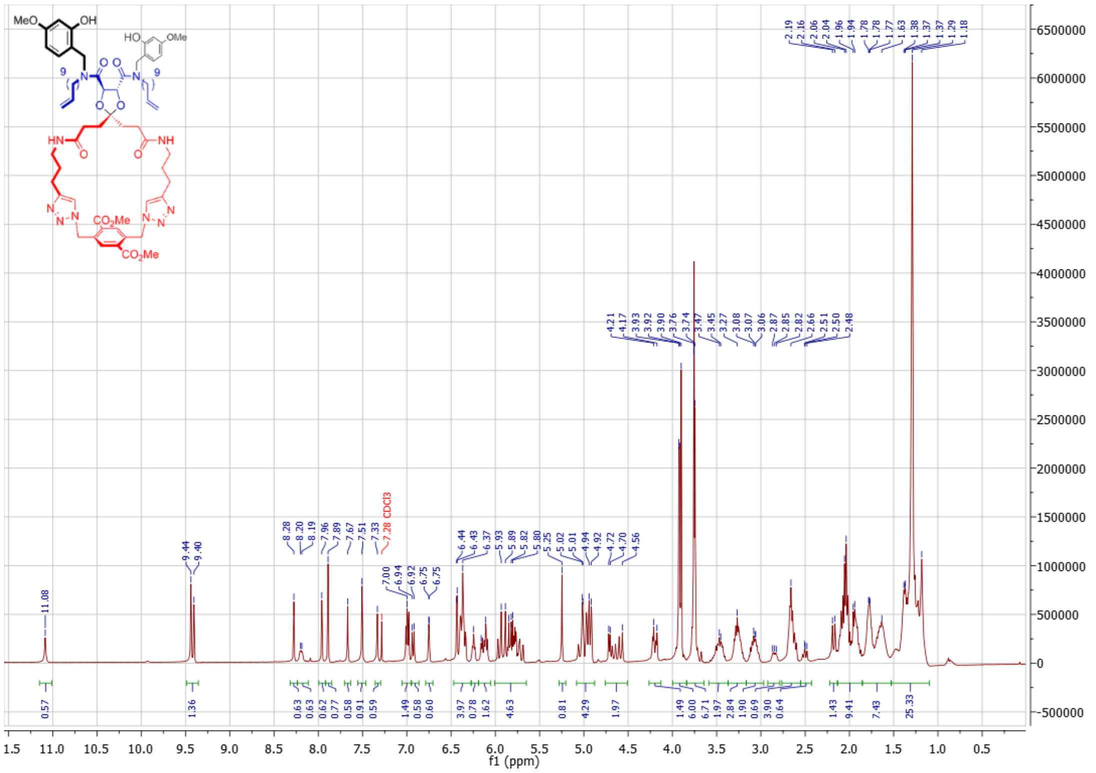

Figure S47. ^{1}H NMR, $CDCl_3$, compound **25a**.

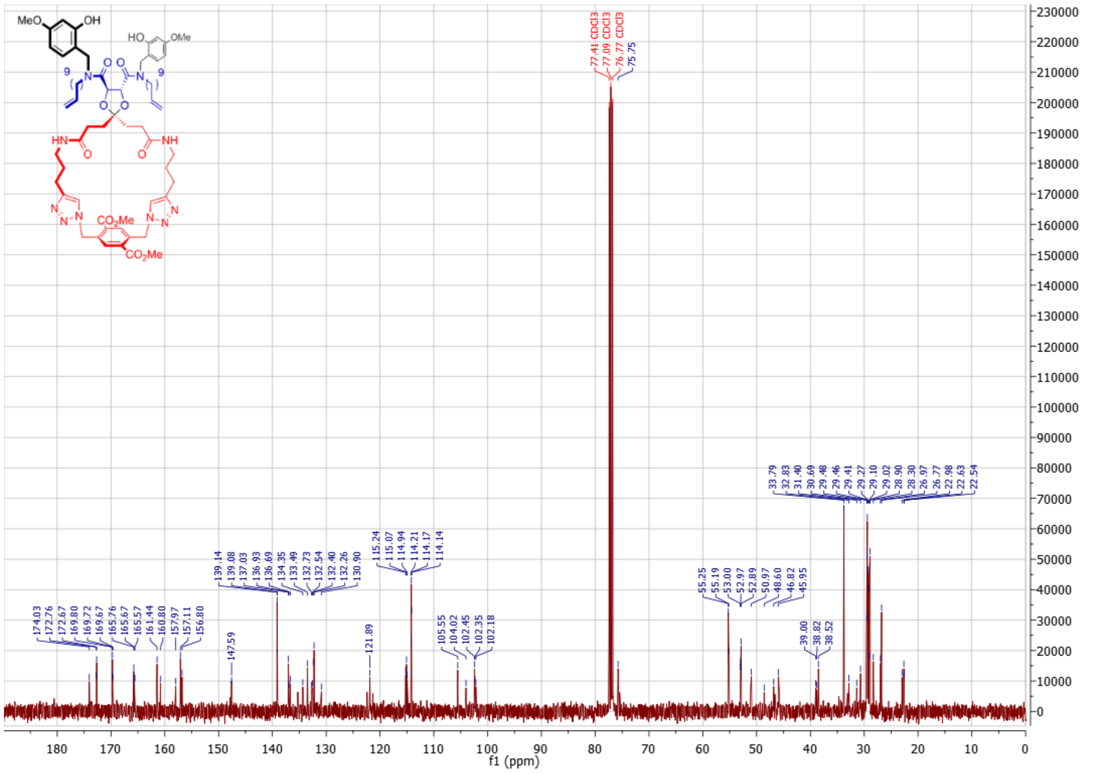

Figure S48. ^{13}C NMR, $CDCl_3$, compound **25a**.

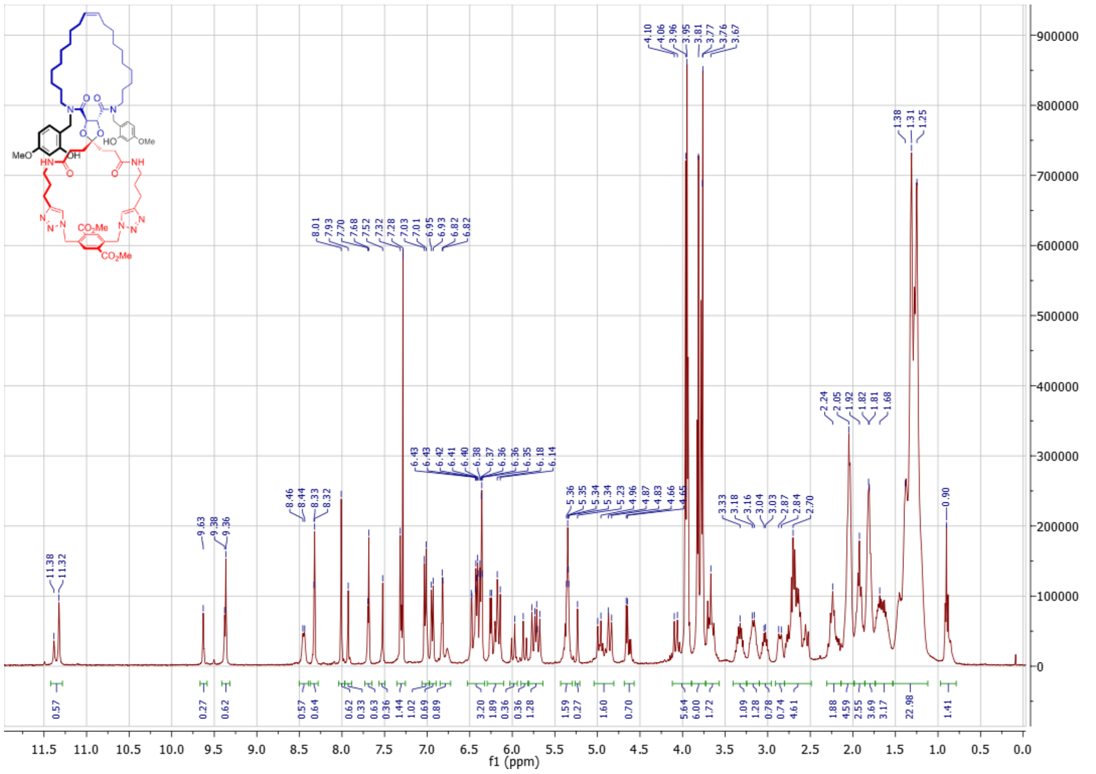

Figure S49. ^{1}H NMR, $CDCl_3$, compound **26a**.

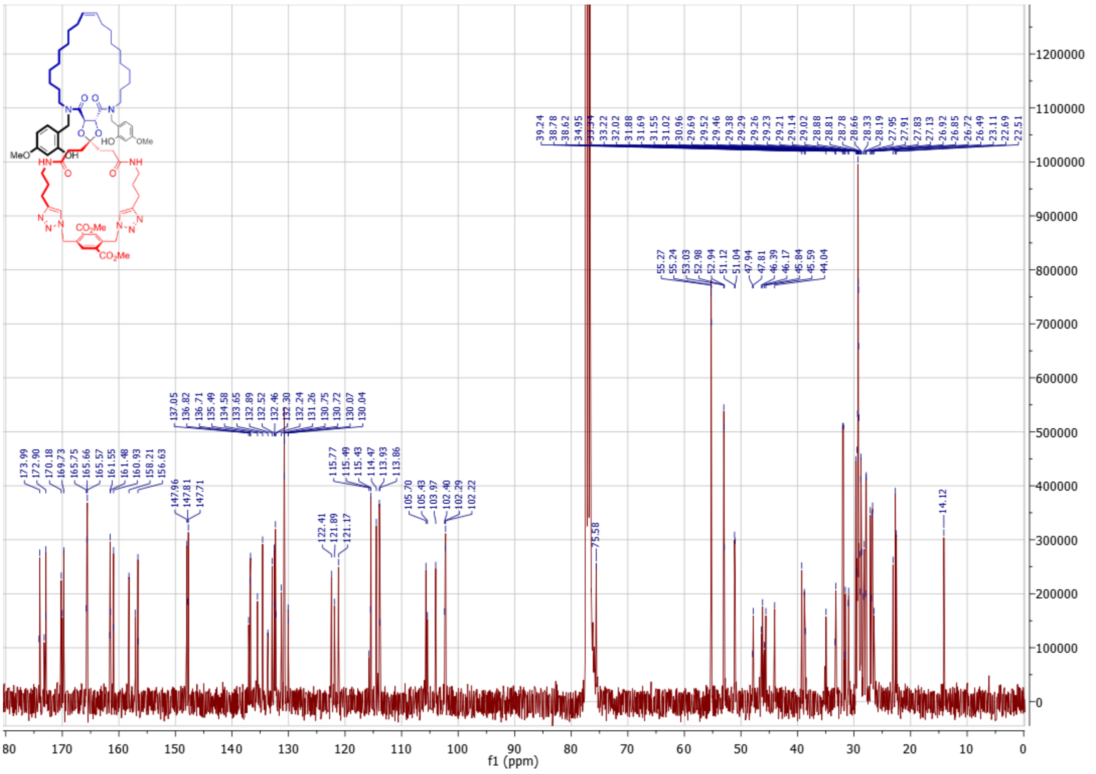

Figure S50. ^{13}C NMR, $CDCl_3$, compound **26a**.

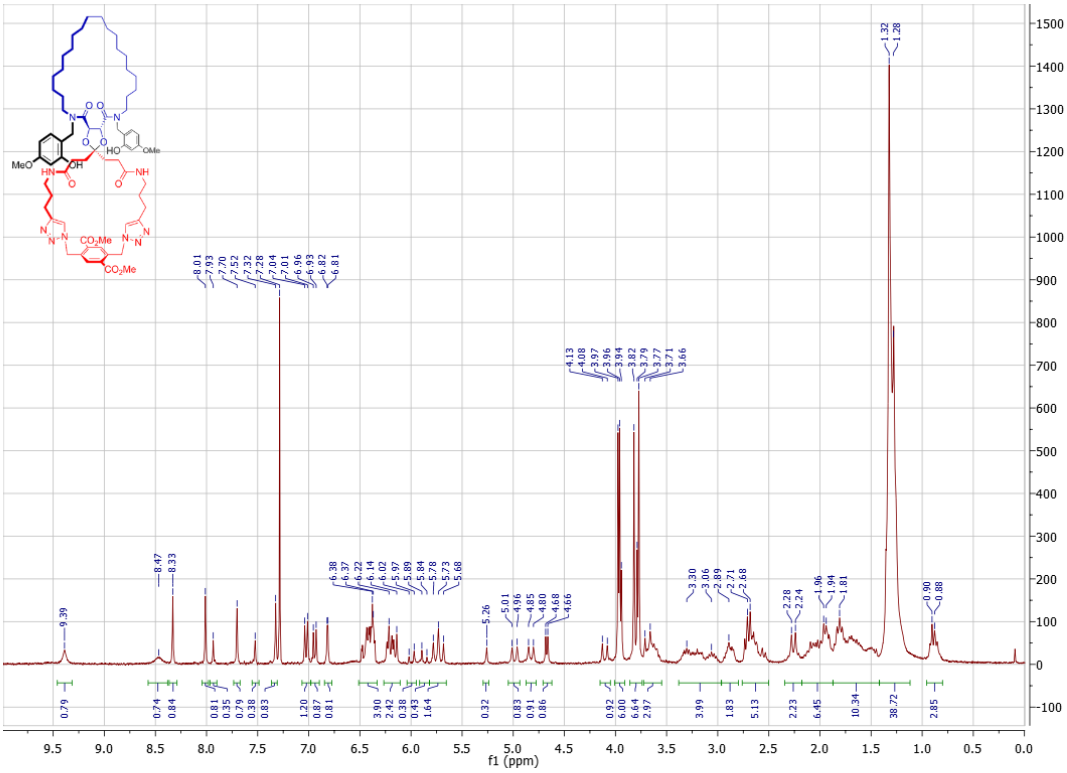

Figure S51. ^{1}H NMR, $CDCl_3$, compound **26a-H$_2$**.

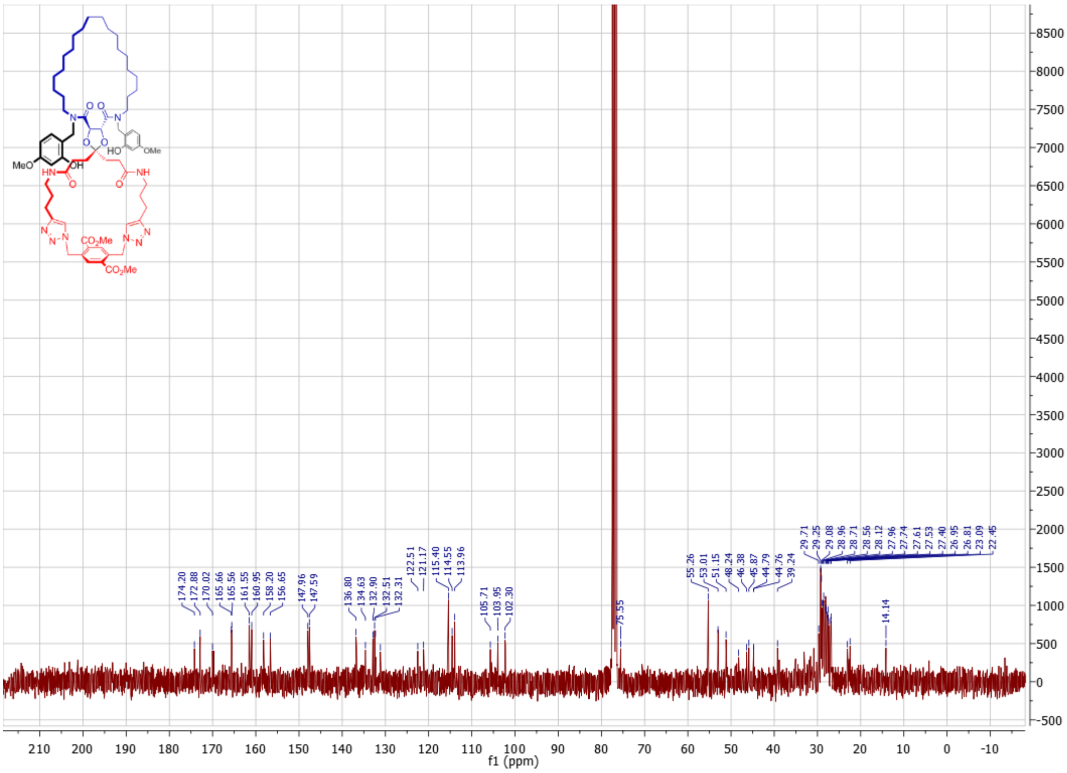

Figure S52. ^{13}C NMR, $CDCl_3$, compound **26a-H$_2$**.

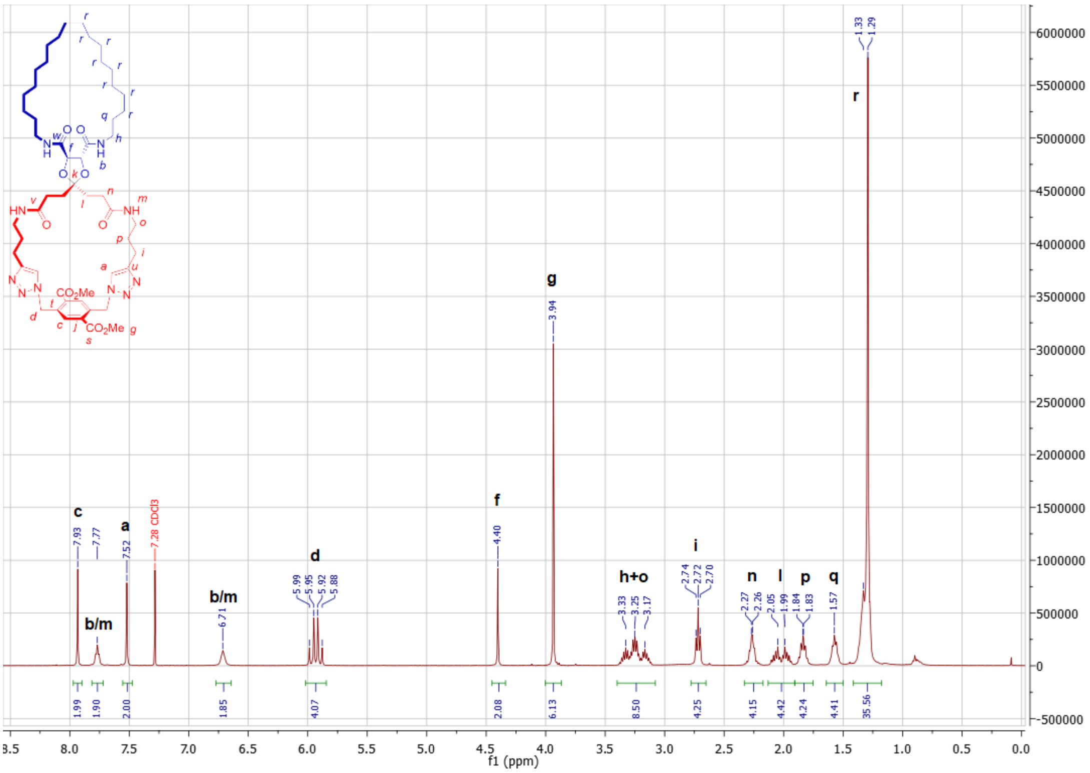

Figure S53. ^{1}H NMR, $CDCl_3$, compound **27a**.

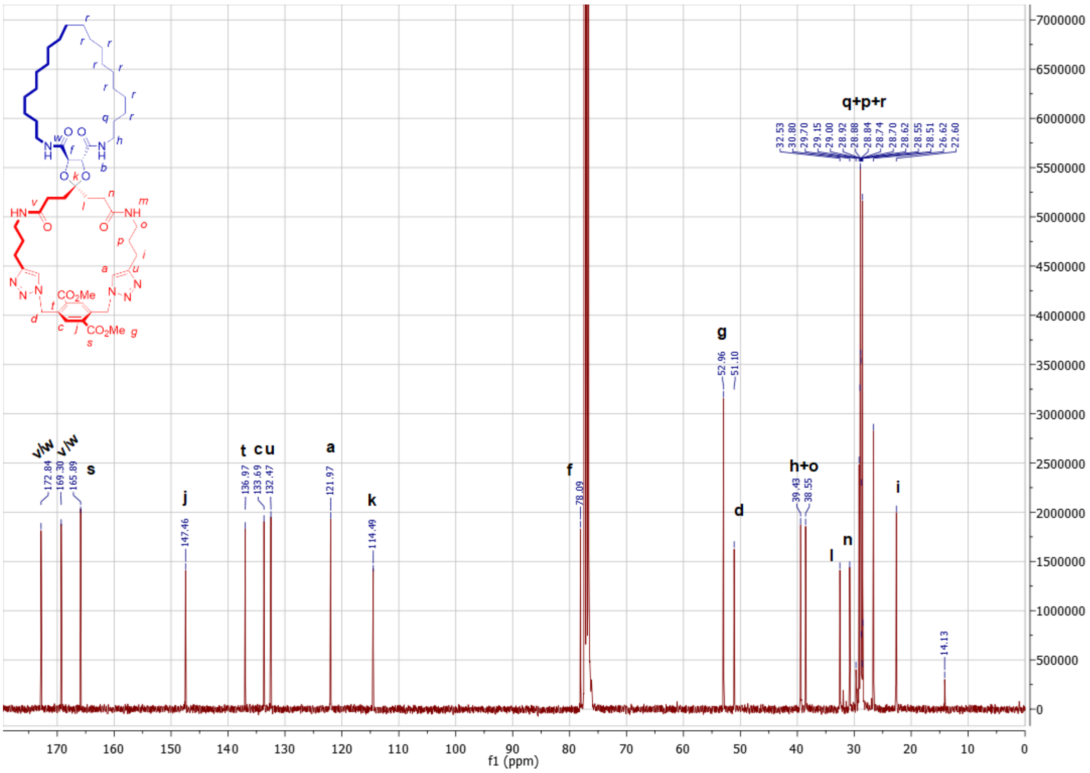

Figure S54. ^{13}C NMR, $CDCl_3$, compound **27a**.

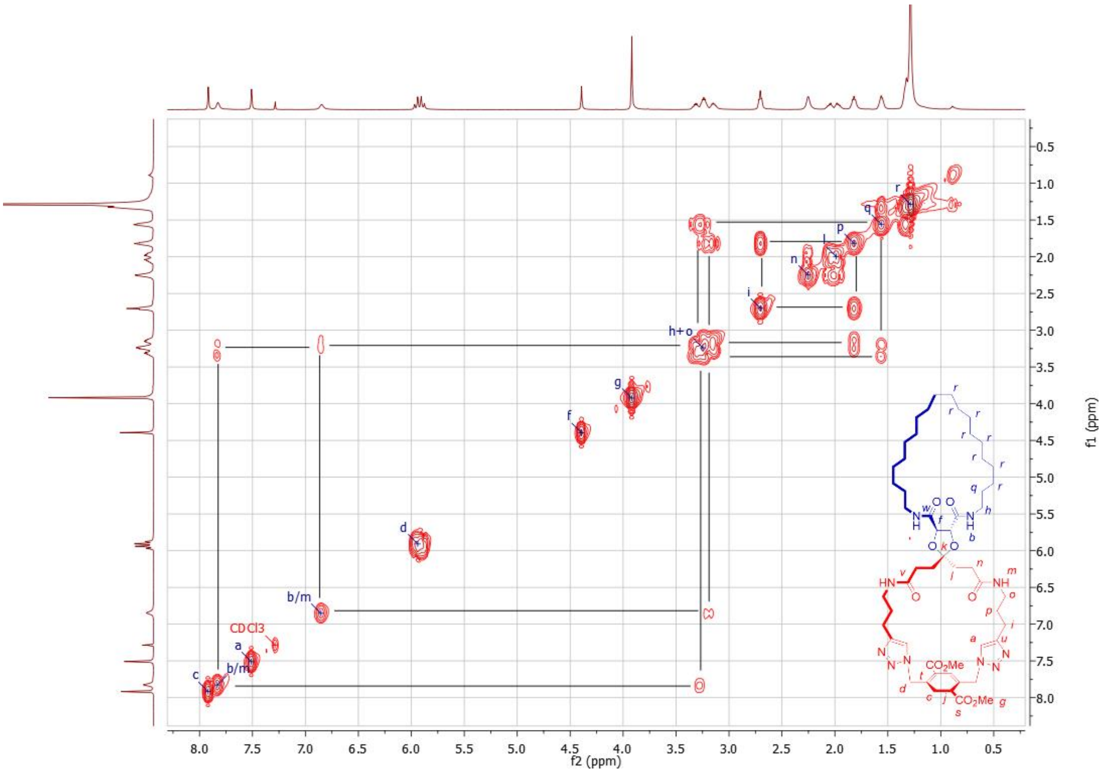

Figure S55. COSY NMR, $CDCl_3$, compound **27a**.

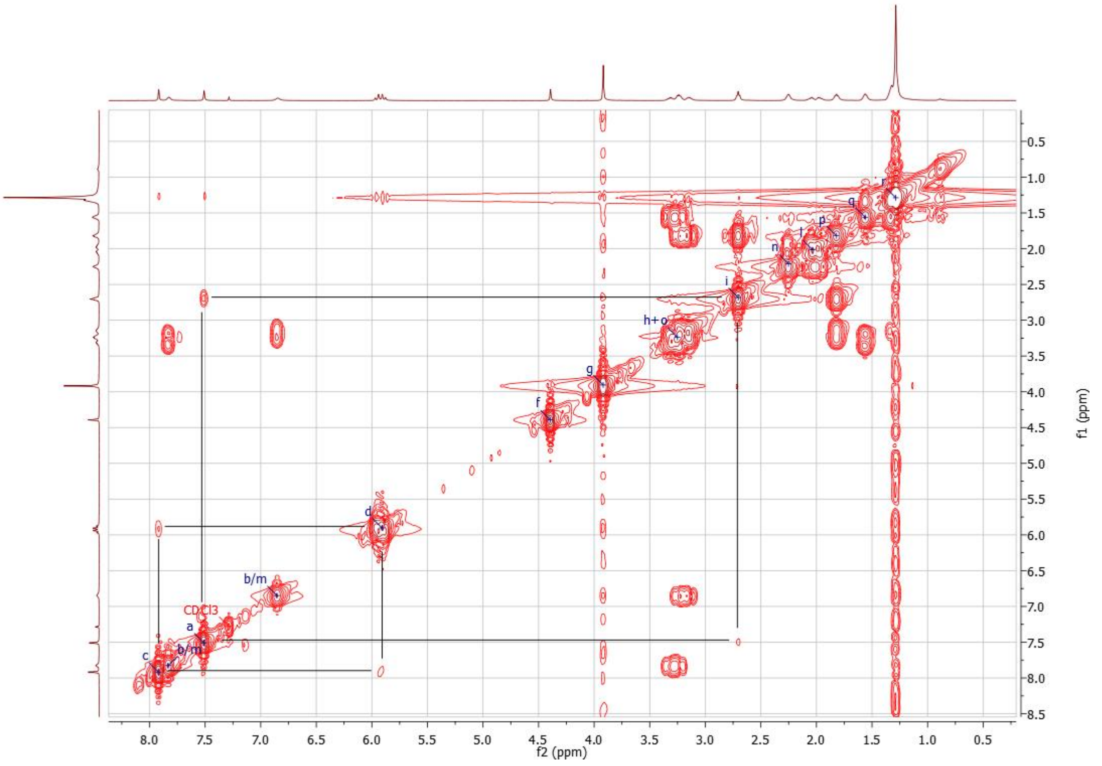

Figure S56. COSY NMR, $CDCl_3$, compound **27a**, with long range $^3J_{HH}$ couplings of the aromatic protons *a* and *c*.

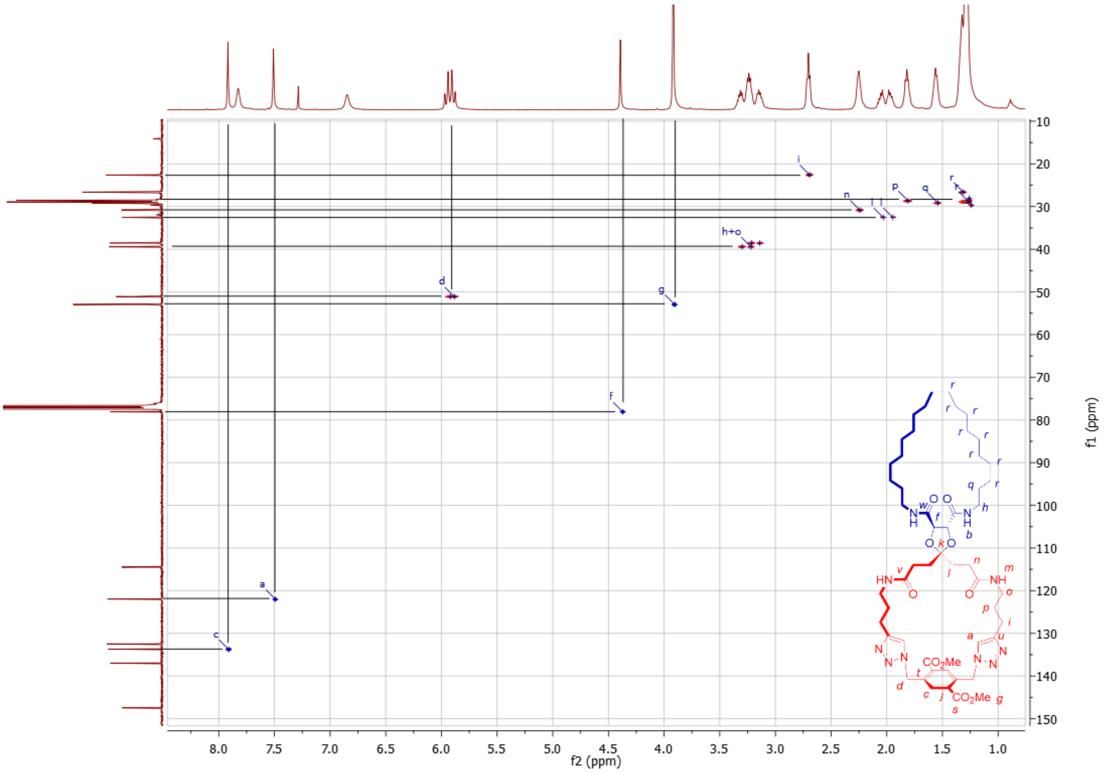

Figure S57. HSQC NMR, $CDCl_3$, compound **27a**.

Mass Spectra

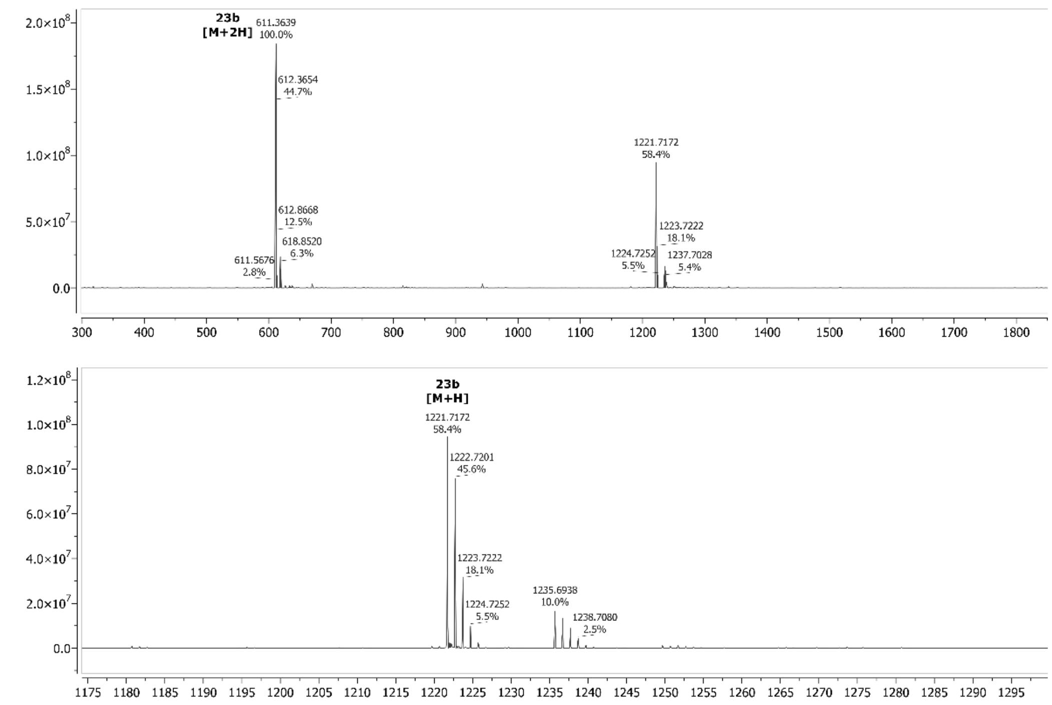

Figure S58. Mass spectrum (ESI+), compound **23b**.

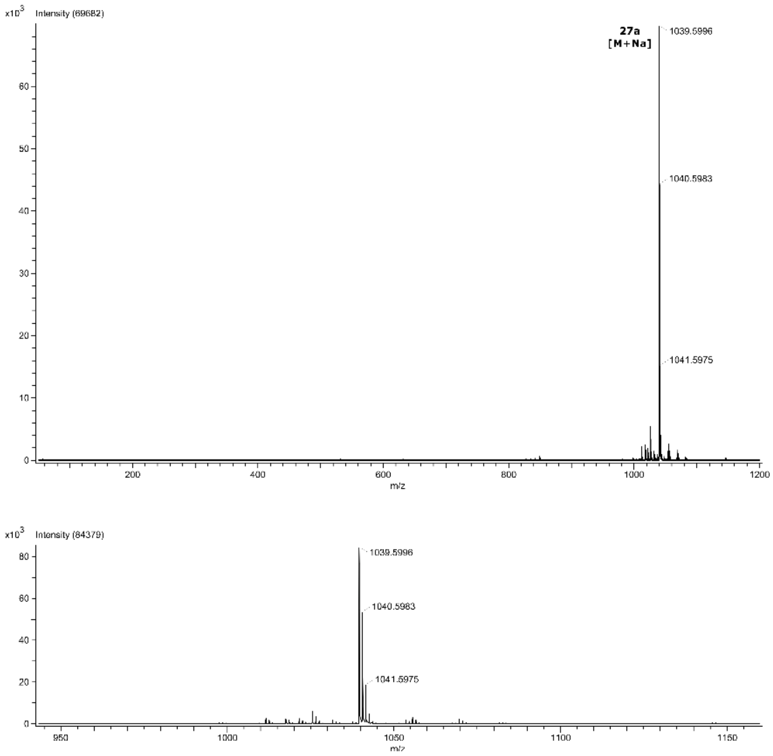

Figure S59. Mass spectrum (FD+), compound **27a**.

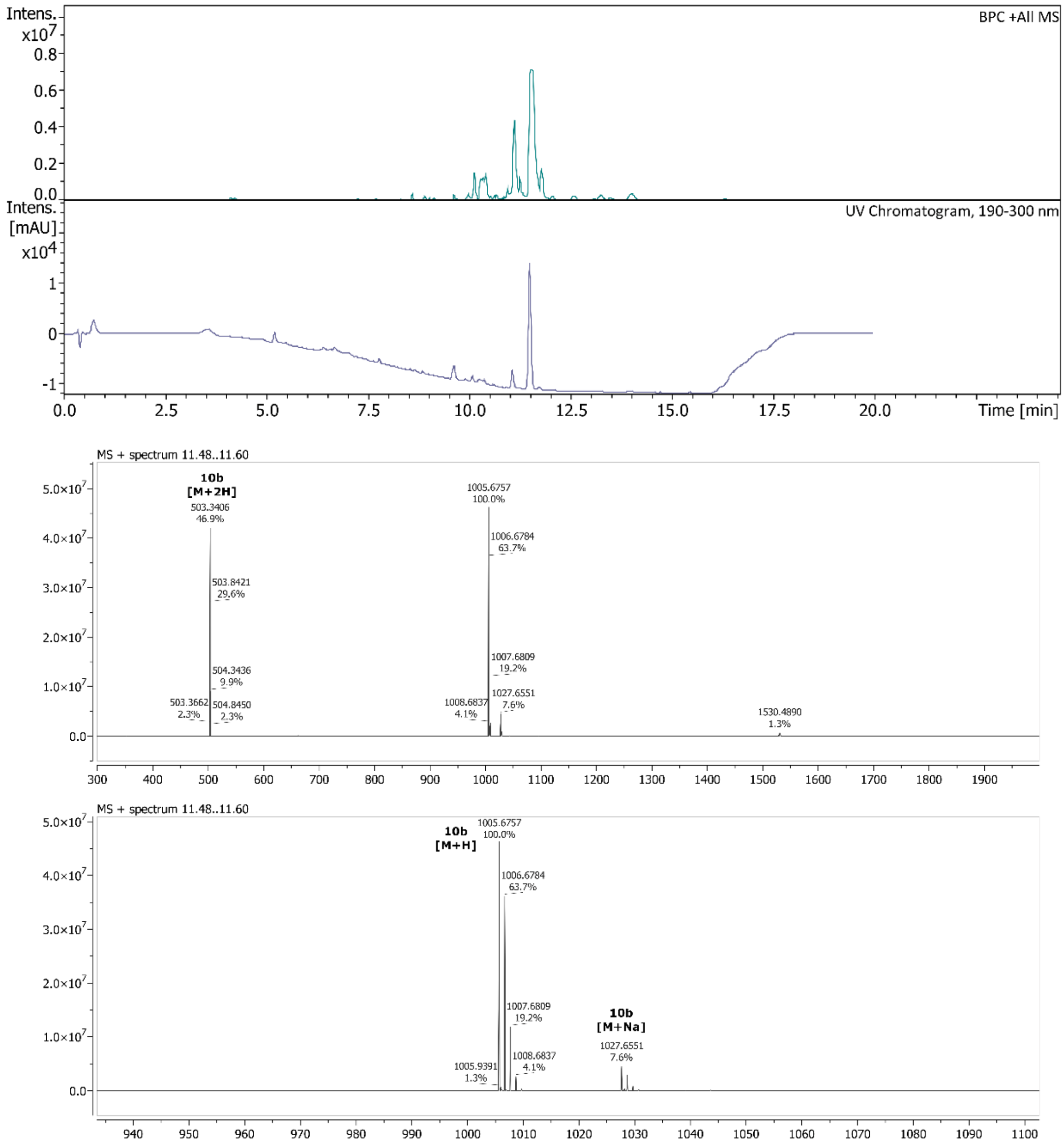

Figure S60. Chromatogram and mass spectrum (ESI+), compound **10b**.

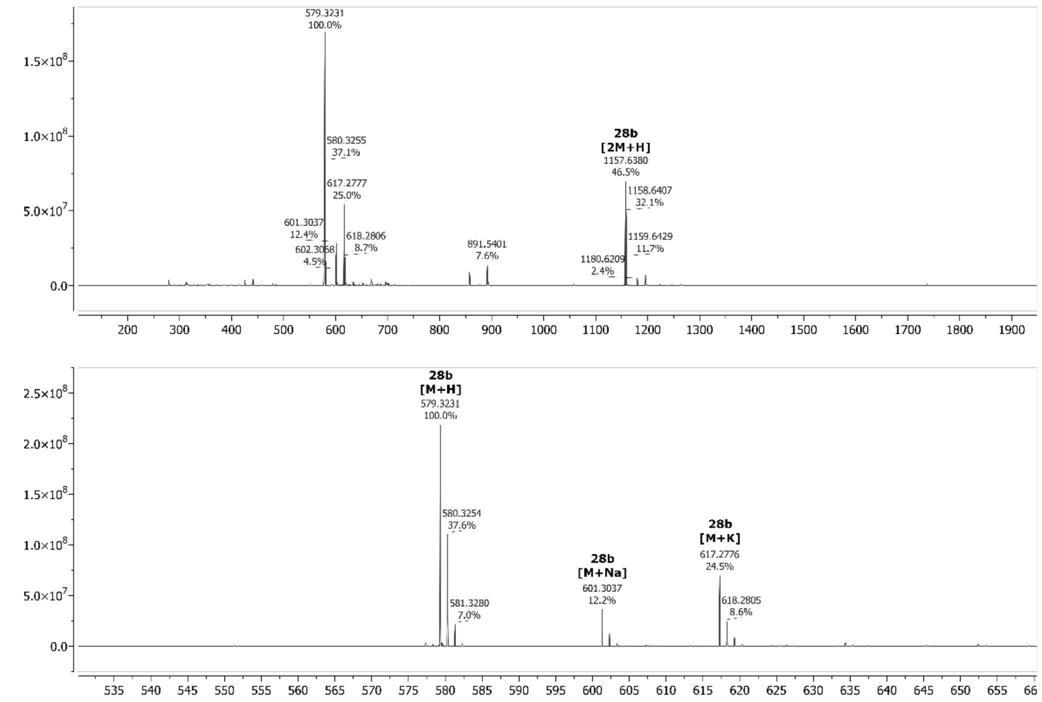

Figure S61. Mass spectrum (ESI+), compound **28b**.

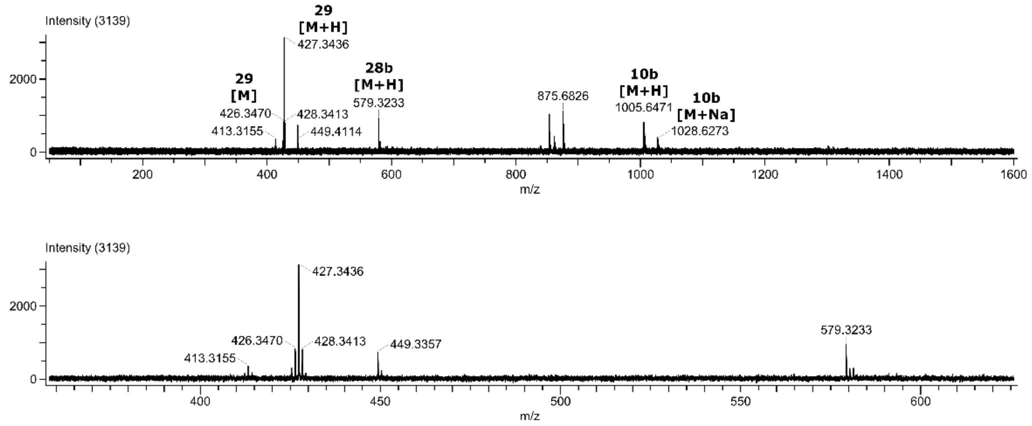

Figure S62. Mass spectrum (ESI+), compound **29**. Trace amounts of ketone ring **28b** and catenane **10b** are visible as well due to their much higher ionizability. Ketone ring **28b** is a co-product of the synthesis of **29**, while catenane **10b** is a byproduct arising from incomplete unwinding of **25b’**.

Molecular mechanics

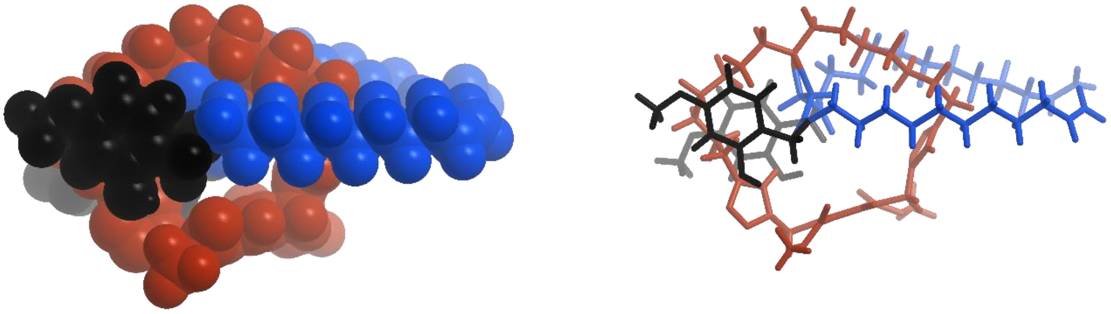

Figure S63. Side view of space filling (left) and tube (right) representations of calculated structure of **25b’** (UFF force field).

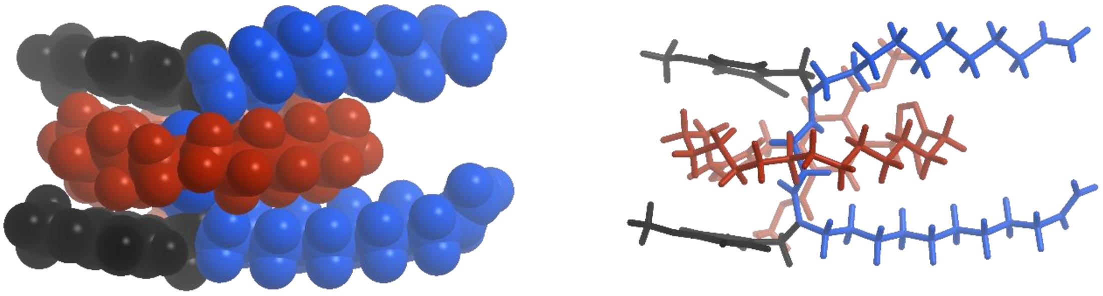

Figure S64. Top view of space filling (left) and tube (right) representations of calculated structure of **25b’** (UFF force field).

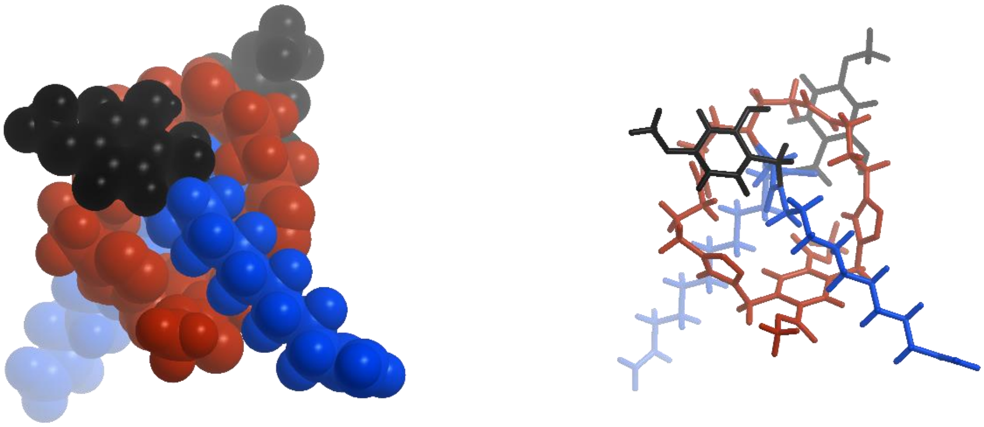

Figure S65. Side view of space filling (left) and tube (right) representations of calculated structure of **25a’** (UFF force field).

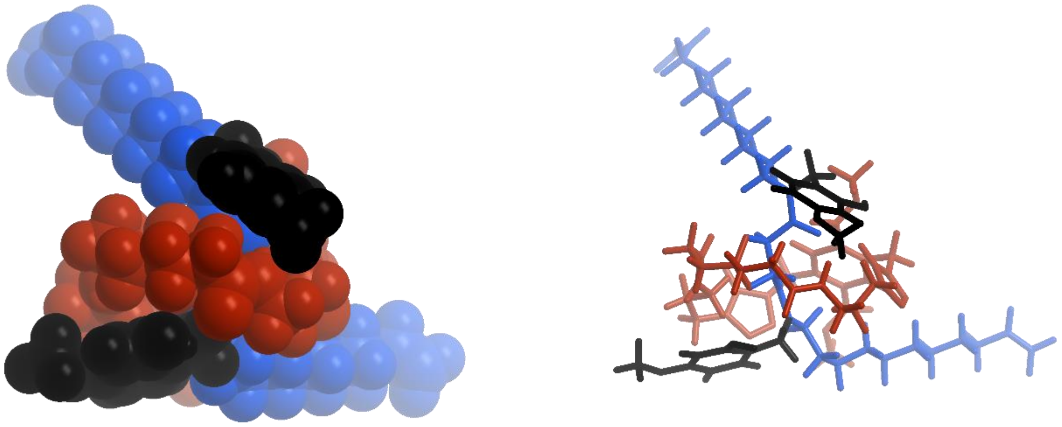

Figure S66. Side view of space filling (left) and tube (right) representations of calculated structure of **25a’** (UFF force field).

XYZ-coordinates of calculated structure of 25b’ (UFF force field)

C	2.4993	2.0542	-3.0032
C	3.4663	1.2137	-2.1663

C	1.4834	1.1521	-3.7111
C	2.7786	-0.0440	3.6453
C	1.3481	-0.5325	3.7405
C	-1.4279	1.1421	-1.6410
C	-1.5422	-0.3589	-1.8733
C	-2.2045	1.6632	-0.4516
C	-0.4297	-1.1652	-1.2394
N	-2.3733	3.0823	-0.2337
N	-0.0404	-2.4331	-1.8081
C	-3.4926	3.5498	0.5987
C	-0.2489	-3.6730	-1.0449
C	-1.7027	-4.0945	-1.0610
C	-4.8098	3.2689	-0.0947
C	-4.9379	3.6784	-1.5161
C	-6.0196	3.3517	-2.2277
C	-7.1179	2.5769	-1.6195
C	-7.0309	2.2454	-0.3120
C	-5.8505	2.6269	0.4966
C	-2.4502	-4.4457	0.1900
C	-3.6262	-5.0848	0.1044
C	-4.2518	-5.3680	-1.2210
C	-3.6121	-4.9736	-2.3278
C	-2.2871	-4.3343	-2.2493
O	-5.8788	2.2947	1.8555
O	-1.9819	-4.1186	1.4636
C	-3.2460	0.6557	-4.5119
C	-1.8615	0.7406	-3.8124
C	-0.7896	1.1113	-4.8940
O	-1.5493	-0.5231	-3.2589
O	-1.9134	1.7353	-2.8087
C	-1.3638	-1.3588	4.1023
C	-1.0422	-0.0413	4.1520
C	0.3688	0.3696	3.9404
C	0.9871	-1.9837	3.6528
C	-0.2949	-2.3497	3.8273
O	-2.7150	0.8528	0.3664
O	-0.1341	-0.9261	-0.0368
C	-2.7587	-1.8931	4.3645
N	3.2537	-0.0827	2.2670
N	-3.7565	-1.2724	3.4997
C	-3.8279	-1.3568	2.0800
C	-5.0810	-1.0736	1.7383
N	-5.8330	-0.8423	2.9250
N	-5.0568	-0.9773	3.9186
N	3.7315	-1.2289	1.6249
N	4.4732	-0.9217	0.6425
C	4.5632	0.4982	0.5807
C	3.8469	1.0087	1.5735
C	0.3988	1.9726	-4.4141
C	4.4229	2.0971	-1.3548
C	5.3405	1.2648	-0.4491
C	-4.4527	0.4577	-3.5721
C	-4.8633	-1.0122	-3.3952
C	-6.2125	-1.1062	-2.6530
C	-6.3240	-2.3205	-1.7168
C	-5.4393	-2.2453	-0.4561
C	-5.6379	-0.9568	0.3520
C	0.7869	-2.4869	-3.0222
C	2.2708	-2.6262	-2.6579
C	3.1522	-2.6922	-3.9160
C	4.5698	-3.2069	-3.6178
C	5.4080	-2.2034	-2.8152
C	6.8066	-2.7615	-2.5264
C	7.6337	-1.7688	-1.7018
C	9.0400	-2.3115	-1.4262
C	9.8618	-1.3104	-0.6088
C	11.2344	-1.8611	-0.3342
C	12.3407	-1.2648	-0.7879
C	-1.3292	4.0363	-0.6350
C	-0.1726	4.0026	0.3660
C	0.9531	4.9531	-0.0567
C	2.1550	4.8220	0.8842
C	3.3664	5.6004	0.3569
C	4.6079	5.3263	1.2144
C	5.8437	6.0264	0.6390
C	7.0911	5.7086	1.4721
C	8.3329	6.3851	0.8850
C	9.5437	6.0685	1.7199
C	10.5871	5.3943	1.2278
O	-8.1978	2.2269	-2.4595
C	-9.2485	1.4222	-1.9215
O	-5.5007	-6.0016	-1.3796
C	-6.1909	-6.4033	-0.1976
C	-2.0304	1.0238	4.4382
O	-1.8971	2.1491	3.8786
O	-3.0123	0.8192	5.4231
C	-3.9375	1.8906	5.6044
C	1.9896	-3.0398	3.3809
O	1.6712	-4.0150	2.6415
O	3.2330	-3.0070	4.0342
C	4.1344	-4.0628	3.7019
H	1.9595	2.7554	-2.3309
H	3.0657	2.6448	-3.7560
H	4.0519	0.5510	-2.8374
H	2.8736	0.5815	-1.4706
H	2.0051	0.5142	-4.4570
H	1.0206	0.4920	-2.9598
H	3.4463	-0.6371	4.2988
H	2.8607	0.9990	4.0249
H	-0.3682	1.4400	-1.5033
H	-2.5021	-0.7468	-1.4761
H	-3.4026	3.0672	1.5909
H	-3.4268	4.6454	0.7718
H	0.3301	-4.5063	-1.4999
H	0.1761	-3.5613	-0.0305
H	-4.1406	4.2348	-1.9934
H	-6.0852	3.6452	-3.2678
H	-7.8127	1.6904	0.1862
H	-4.1398	-5.3369	1.0221
H	-4.0519	-5.1532	-3.3009
H	-1.7677	-4.1236	-3.1749
H	-5.3218	2.8939	2.4478
H	-1.2934	-3.3824	1.4635
H	-3.2258	-0.1394	-5.2895
H	-3.4207	1.6161	-5.0468
H	-1.2681	1.6981	-5.7088
H	-0.4105	0.1851	-5.3797
H	0.6124	1.4246	3.9969
H	-0.5580	-3.4009	3.8004
H	-2.7981	-2.9928	4.2109
H	-3.0220	-1.7140	5.4283
H	-3.0075	-1.5904	1.4157
H	3.7528	2.0511	1.8394
H	0.8649	2.4526	-5.3029
H	0.0422	2.8010	-3.7670
H	3.8408	2.8076	-0.7332
H	5.0525	2.6919	-2.0517
H	5.9251	0.5590	-1.0739

H	6.0586	1.9382	0.0665
H	-4.2652	0.9142	-2.5777
H	-5.3175	0.9994	-4.0164
H	-4.9732	-1.4953	-4.3907
H	-4.0661	-1.5611	-2.8612
H	-6.4246	-0.1816	-2.0793
H	-7.0222	-1.1760	-3.4120
H	-7.3839	-2.4132	-1.3935
H	-6.0760	-3.2430	-2.2838
H	-5.6990	-3.1146	0.1853
H	-4.3695	-2.3419	-0.7332
H	-5.1277	-0.1162	-0.1618
H	-6.7215	-0.7215	0.4250
H	0.4725	-3.3528	-3.6437
H	0.6407	-1.5875	-3.6470
H	2.5712	-1.7589	-2.0342
H	2.4145	-3.5496	-2.0574
H	2.6908	-3.3903	-4.6486
H	3.2040	-1.6901	-4.3938
H	4.5079	-4.1737	-3.0713
H	5.0794	-3.4025	-4.5866
H	5.5045	-1.2609	-3.3963
H	4.9040	-1.9804	-1.8521
H	6.7138	-3.7166	-1.9636
H	7.3265	-2.9662	-3.4883
H	7.7137	-0.8080	-2.2569
H	7.1202	-1.5805	-0.7333
H	8.9612	-3.2697	-0.8659
H	9.5562	-2.5072	-2.3922
H	9.9236	-0.3448	-1.1586
H	9.3596	-1.1195	0.3640
H	11.3274	-2.7682	0.2561
H	13.3145	-1.6900	-0.5672
H	12.2884	-0.3566	-1.3805
H	-0.9589	3.8006	-1.6533
H	-1.7451	5.0660	-0.6891
H	-0.5472	4.2929	1.3720
H	0.2225	2.9646	0.4258
H	1.2668	4.7065	-1.0941
H	0.5837	6.0019	-0.0422
H	1.8797	5.1985	1.8938
H	2.4223	3.7493	0.9751
H	3.5746	5.2949	-0.6922
H	3.1383	6.6889	0.3670
H	4.4260	5.6845	2.2515
H	4.7982	4.2313	1.2478
H	6.0057	5.6839	-0.4071
H	5.6739	7.1257	0.6302
H	6.9359	6.0617	2.5159
H	7.2507	4.6073	1.4913
H	8.4763	6.0490	-0.1661
H	8.1904	7.4872	0.8772
H	9.5707	6.4029	2.7531
H	11.4444	5.1863	1.8598
H	10.5972	5.0435	0.2003
H	-9.9917	1.2253	-2.7210
H	-8.8532	0.4444	-1.5714
H	-9.7657	1.9571	-1.0965
H	-5.5878	-7.1397	0.3758
H	-6.4377	-5.5206	0.4300
H	-7.1429	-6.8918	-0.4893
H	-4.4968	2.0819	4.6639
H	-4.6657	1.6069	6.3915
H	-3.4091	2.8120	5.9316
H	5.0789	-3.9195	4.2657
H	3.7032	-5.0470	3.9861
H	4.3694	-4.0463	2.6155

XYZ-coordinates of calculated structure of 25a’ (UFF force field)

N	2.1474	-0.7764	3.9728
C	1.2525	0.3804	3.9892
C	3.1075	-0.9587	3.0144
C	-0.9569	4.7712	1.4373
C	-1.4280	4.0240	0.2108
C	1.9769	-2.0434	0.0133
C	0.6380	-2.3776	0.6712
C	1.9721	-2.0583	-1.5022
C	-0.2238	-1.1998	1.0844
N	3.1465	-1.9832	-2.2327
N	-1.5803	-1.3915	1.5719
C	3.1567	-2.4246	-3.6396
C	-1.9371	-2.6277	2.3120
C	-1.4776	-2.5858	3.7617
C	2.8681	-3.9114	-3.7140
C	3.8848	-4.8065	-3.1063
C	3.6983	-6.1273	-3.0591
C	2.4769	-6.7369	-3.6150
C	1.5591	-5.9341	-4.1975
C	1.7440	-4.4653	-4.2504
C	-0.6044	-3.6536	4.3530
C	-0.0552	-3.4712	5.5624
C	-0.3968	-2.2759	6.3860
C	-1.3000	-1.4117	5.9118
C	-1.8571	-1.5669	4.5553
O	0.7007	-3.7354	-4.8365
O	-0.3955	-4.8856	3.7295
C	2.1272	-5.1996	1.2231
C	2.2422	-3.6785	1.5472
C	3.0892	-3.5146	2.8484
O	0.9586	-3.1460	1.7852
O	2.8722	-3.0167	0.4723
C	-2.1604	2.6971	-2.2074
C	-0.9185	2.5314	-1.6876
C	-0.5655	3.2031	-0.4124
C	-2.7848	4.2377	-0.3796
C	-3.1023	3.6237	-1.5325
O	0.8787	-2.2209	-2.1085
O	0.1868	-0.0291	0.8562
C	-2.6796	1.9102	-3.3961
N	-0.9291	3.8796	2.5894
N	-3.0138	0.5456	-2.9964
C	-2.1486	-0.5842	-2.9521
C	-2.9214	-1.6596	-2.8541
N	-4.2837	-1.2439	-2.8828
N	-4.3093	0.0209	-2.9834
N	-2.0353	3.2258	3.1347
N	-1.7078	2.6041	4.1906
C	-0.3174	2.8237	4.4248
C	0.1598	3.6069	3.4622
C	3.9210	-2.2207	2.9737
C	1.6261	1.3425	5.1236
C	0.4497	2.2287	5.5702
C	1.5006	-5.5715	-0.1404
C	-0.0056	-5.6224	-0.1359
N	-0.7103	-5.1189	-1.2013
C	-2.1751	-5.1234	-1.2929
C	-2.7258	-3.6942	-1.4228
C	-2.4618	-3.0850	-2.8065
O	-0.5987	-6.2604	0.7755
O	3.4191	-0.0109	2.2422
C	-2.4934	-0.2260	1.4876

C	-3.9883	-0.5715	1.6446
C	-4.8990	0.6570	1.4818
C	-5.2471	0.9241	0.0144
C	-6.2370	2.0865	-0.1179
C	-6.7135	2.2417	-1.5671
C	-7.5419	3.5191	-1.7411
C	-7.9988	3.6834	-3.1951
C	-8.8044	4.9729	-3.3761
C	-9.2328	5.1240	-4.8104
C	-10.5204	5.1572	-5.1656
C	4.3861	-1.4061	-1.6774
C	4.4587	0.0867	-2.0038
C	5.7197	0.7269	-1.4145
C	5.7740	2.2236	-1.7415
C	7.0381	2.8698	-1.1631
C	7.0897	4.3662	-1.4931
C	8.3580	5.0131	-0.9241
C	8.4141	6.5070	-1.2652
C	9.6799	7.1548	-0.6967
C	9.7288	8.6139	-1.0608
C	9.7252	9.5763	-0.1339
O	2.3482	-8.1380	-3.4957
C	1.1358	-8.7515	-3.9358
O	0.1566	-2.0142	7.6551
C	1.1863	-2.8882	8.1142
C	0.1387	1.7596	-2.3706
O	0.9433	1.0750	-1.6796
O	0.3624	1.9683	-3.7421
C	1.3586	1.1394	-4.3366
C	-3.8082	5.0675	0.2928
O	-4.6033	5.7597	-0.4051
O	-3.9799	4.9486	1.6826
C	-5.0523	5.7070	2.2402
H	2.0005	-1.5123	4.6990
H	1.2867	0.9292	3.0242
H	0.2154	0.0210	4.1123
H	-1.6036	5.6414	1.6639
H	0.0596	5.1846	1.2557
H	2.3378	-1.0504	0.3347
H	0.0243	-2.9941	-0.0038
H	2.4827	-1.7863	-4.2313
H	4.1562	-2.2551	-4.0968
H	-3.0240	-2.8261	2.2917
H	-1.5205	-3.4938	1.7704
H	4.7886	-4.3875	-2.6815
H	4.4486	-6.7583	-2.5996
H	0.6487	-6.3309	-4.6234
H	0.5787	-4.2517	5.9605
H	-1.5858	-0.5495	6.5008
H	-2.5162	-0.7923	4.1997
H	0.7394	-2.7328	-4.7605
H	-1.1140	-5.1287	3.0641
H	1.5923	-5.7205	2.0463
H	3.1557	-5.6236	1.2049
H	3.8256	-4.3440	2.9273
H	2.4299	-3.6242	3.7361
H	0.4287	3.0610	-0.0028
H	-4.0819	3.7754	-1.9666
H	-3.5973	2.3957	-3.7928
H	-1.9675	1.9005	-4.2427
H	-1.0719	-0.5684	-3.0222
H	1.1763	3.9593	3.3455

H	4.5080	-2.2753	3.9158
H	4.6557	-2.1716	2.1403
H	1.9744	0.7639	6.0074
H	2.4702	1.9843	4.7893
H	0.8347	3.0483	6.2137
H	-0.2462	1.6189	6.1851
H	1.8718	-4.8915	-0.9332
H	1.8534	-6.5899	-0.4124
H	-0.1763	-4.7331	-2.0102
H	-2.4849	-5.7310	-2.1702
H	-2.6292	-5.5893	-0.3921
H	-3.8238	-3.7213	-1.2486
H	-2.2785	-3.0526	-0.6356
H	-3.0018	-3.6644	-3.5855
H	-1.3807	-3.1241	-3.0436
H	-2.2016	0.5162	2.2597
H	-2.3611	0.2602	0.4960
H	-4.2724	-1.3618	0.9151
H	-4.1714	-0.9667	2.6644
H	-4.4243	1.5564	1.9331
H	-5.8435	0.4636	2.0369
H	-5.6985	0.0087	-0.4276
H	-4.3189	1.1710	-0.5407
H	-5.7421	3.0209	0.2207
H	-7.1211	1.9053	0.5322
H	-7.3304	1.3587	-1.8446
H	-5.8387	2.2776	-2.2496
H	-6.9264	4.3985	-1.4496
H	-8.4322	3.4738	-1.0757
H	-8.6289	2.8130	-3.4839
H	-7.1067	3.7107	-3.8601
H	-8.1755	5.8480	-3.1047
H	-9.6857	4.9583	-2.6968
H	-8.4704	5.2143	-5.5789
H	-10.7912	5.2699	-6.2103
H	-11.3109	5.0712	-4.4263
H	4.4584	-1.5416	-0.5816
H	5.2715	-1.9267	-2.1033
H	4.4580	0.2185	-3.1077
H	3.5624	0.5916	-1.5842
H	5.7202	0.5914	-0.3101
H	6.6184	0.2235	-1.8345
H	5.7643	2.3591	-2.8457
H	4.8766	2.7254	-1.3163
H	7.0481	2.7356	-0.0588
H	7.9350	2.3679	-1.5888
H	7.0726	4.4998	-2.5974
H	6.1955	4.8689	-1.0626
H	8.3711	4.8865	0.1810
H	9.2522	4.5056	-1.3489
H	8.4009	6.6320	-2.3709
H	7.5202	7.0161	-0.8412
H	9.7020	7.0193	0.4076
H	10.5781	6.6578	-1.1222
H	9.7741	8.8914	-2.1100
H	9.7642	10.6181	-0.4350
H	9.6821	9.3387	0.9246
H	1.1955	-9.8413	-3.7385
H	0.2671	-8.3461	-3.3730
H	1.0007	-8.6085	-5.0293
H	1.5540	-2.5221	9.0946
H	0.7895	-3.9154	8.2607
H	2.0413	-2.8916	7.4038
H	1.4295	1.3802	-5.4168
H	2.3498	1.3257	-3.8720
H	1.0760	0.0713	-4.2350
H	-5.0813	5.5353	3.3357
H	-4.8960	6.7925	2.0594
H	-6.0238	5.3813	1.8091

References

[1] L. Steemers, M. J. Wanner, B. R. C. van Leeuwen, H. Hiemstra, J. H. van Maarseveen, *Eur. J. Org. Chem.* **2018**, *2018*, 874–878.
[2] T. E. Hopkins, K. B. Wagener, *Macromolecules* **2003**, *36*, 2206–2214.
[3] L. Steemers, M. J. Wanner, M. Lutz, H. Hiemstra, J. H. van Maarseveen, *Nat. Commun.* **2017**, *8*, 15392.
